# Regulation of Apoptotic Mediators Reveals Dynamic Responses to Thermal Stress in the Reef Building Coral *Acropora millepora*

**DOI:** 10.1371/journal.pone.0016095

**Published:** 2011-01-24

**Authors:** Mathieu Pernice, Simon R. Dunn, Thomas Miard, Sylvie Dufour, Sophie Dove, Ove Hoegh-Guldberg

**Affiliations:** 1 Coral Reef Ecosystem Laboratory, Global Change Institute, ARC Centre for Excellence in Coral Reef Studies, The University of Queensland, St Lucia, Queensland, Australia; 2 UMR 7208 “Biologie des Organismes et Ecosystèmes Aquatiques” MNHN-CNRS-IRD–UPMC, Paris, France; Northeastern University, United States

## Abstract

**Background:**

Mass coral bleaching is increasing in scale and frequency across the world's coral reefs and is being driven primarily by increased levels of thermal stress arising from global warming. In order to understand the impacts of projected climate change upon corals reefs, it is important to elucidate the underlying cellular mechanisms that operate during coral bleaching and subsequent mortality. In this respect, increased apoptotic cell death activity is an important cellular process that is associated with the breakdown of the mutualistic symbiosis between the cnidarian host and their dinoflagellate symbionts.

**Methodology/Principal Findings:**

The present study reports the impacts of different stressors (colchicine and heat stress) on three phases of apoptosis: (i) the potential initiation by differential expression of Bcl-2 members, (ii) the execution of apoptotic events by activation of caspase 3-like proteases and (iii) and finally, the cell disposal indicated by DNA fragmentation in the reef building coral *Acropora millepora*. In corals incubated with colchicine, an increase in caspase 3-like activity and DNA fragmentation was associated with a relative down-regulation of Bcl-2, suggesting that the initiation of apoptosis may be mediated by the suppression of an anti-apoptotic mechanism. In contrast, in the early steps of heat stress, the induction of caspase-dependent apoptosis was related to a relative up-regulation of Bcl-2 consecutively followed by a delayed decrease in apoptosis activity.

**Conclusions/Significance:**

In the light of these results, we propose a model of heat stress in coral hosts whereby increasing temperatures engage activation of caspase 3-dependent apoptosis in cells designated for termination, but also the onset of a delayed protective response involving overexpression of Bcl-2 in surviving cells. This mitigating response to thermal stress could conceivably be an important regulatory mechanism for cell survival in corals exposed to sudden environmental changes.

## Introduction

Our ability to predict the impacts of climate change on coral reef is dependent on deciphering the cellular and molecular mechanisms that drive sensitivity of corals to thermal stress and bleaching (breakdown of the symbiosis between coral host and its dinoflagellate symbionts, *Symbiodinium*). In this respect, significant progress has been made during the past two decades towards understanding the mechanisms of bleaching [1]. A wide array of cellular mechanisms has been proposed to explain the loss of symbionts from the coral tissue including exocytosis, pinching off, host cell detachment, and cell death pathway activation [2], [3].

The susceptibility to bleaching can vary greatly amongst species and geographical locations, and may be attributed to both differences in thermal tolerance among symbionts clades and sub-clades [4], [5] and/or the coral host and its capacity to tolerate stress by employing a wide variety of protective responses [6], [7], [8], [9]. One of the first sites damaged in response to thermal stress is the symbiont chloroplast, where photosynthetic dysfunction results in the overproduction of reactive oxygen species (ROS), leading to symbiont and host cell damage, through a variety of possible mechanisms [1]. One suite of the molecular mechanisms that results in the removal of highly compromised symbionts from stressed host tissues involves regulation of cell death pathways which play active roles in maintaining tissue homeostasis and immune responses [2], [6], [10], [11].

Apoptosis is one of the main types of programmed cell death and is active in response to various physiological and pathological situations [12]. In the basal phylum Cnidaria, the initiation of cell death has been shown to occur during development [13], hyperthermic stress [2], [6], [10], ultraviolet radiation [14], chemical induction [13], [15] immune response to disease [16] and onset of symbiosis [17]. The apoptotic pathway is dependent upon the activation of proteolytic enzymes named caspases [18]. Caspase activation can be initiated through either the extrinsic (cell surface death receptor) pathway or the intrinsic pathway [19] which involves the permeabilisation of the mitochondrial outer membrane and subsequent release of cytochrome *c* into the cytosol. The regulation of the mitochondria intrinsic pathway is performed by a complex protein network in which the B-cell lymphoma protein-2 (Bcl-2) family members form a central checkpoint that determines whether a cell lives or dies [20]. Bcl-2 proteins are divided between pro-apoptotic (promoting apoptosis) and anti-apoptotic (inhibiting apoptosis) members according to their Bcl-2 Homology (BH) domains and associated function [21]. Among the pro-apoptotic members, Bcl-2-associated X (Bax) and Bcl-2-antagonist/killer-1 (Bak) promote apoptosis through their oligomerization and insertion in the mitochondrial outer membrane where they form large pores [21]. Bax and Bak activation is controlled by the interplay and heterodimer formation with anti-apoptotic members, such as Bcl-2 [22]. Bcl-2 is potentially the most characterised anti-apoptotic member, and functions by regulating transcription, caspase activation, mitochondrial membrane pore formation, intracellular Ca^2+^ homeostasis and by increasing cellular resistance to oxidative stress [23], [24]. Consequently, it has been suggested that the ratios between anti-apoptotic Bcl-2 and pro-apoptotic Bax and Bak may be more important than either promoter alone in determining apoptosis [23], [24]. These ratios can therefore be used as prognostic markers to study apoptosis regulation [25].

Although direct evidence of mitochondrial outer membrane permeabilisation is still lacking in the more basal phyla [21], extensive studies have revealed that the general outline of the apoptotic machinery is conserved among metazoans [18], [26], [27]. In the Cnidaria, a basal metazoan phylum, both anti-apoptotic and pro-apoptotic-like Bcl-2 sequences have been identified in the hydrozoan *Hydra magnipapillata* [28], the non-symbiotic anthozoan, *Nematostella vectensis* [21], [28], [29], the symbiotic anthozoans *Aiptasia pulchella* [30] and the reef building corals *Acropora aspera* [11] and *A. millepora* [11], [31]. Furthermore, there is now increasing evidence that activation of caspase-dependent apoptosis plays a role during cnidarian bleaching [6], [10], [32]. However, a direct link between the molecular regulation of the Bcl-2 family of apoptotic mediators and the activation of cell death in corals is still yet to be shown.

The present study aimed to provide an integrated picture of the regulation of the mitochondrial apoptotic cascade by Bcl-2 family members within the reef building coral *A. millepora*. In this respect, we used (i) quantitative Real-Time PCR to monitor the potential initiation of apoptosis by differential expression of Bcl-2, Bax and Bak, (ii) fluorometric assay of downstream caspase 3-like activity to measure the execution of apoptotic events and (iii) TUNEL labelling to detect the completion of cell death via DNA fragmentation. Regulation of these apoptotic mediators was investigated in complement to indicators of coral bleaching (symbiont cells density and chlorophyll *a* content) in response to defined stressors and different thermal treatments. This work aims to bring further insight into the characterization of the apoptotic pathways and their functional activation in symbiotic Cnidaria during thermal stress.

## Results

### A. Effect of defined stimuli (colchicine and fast heat stress)

#### Apoptosis-like activity in host tissue

**Figure 1:**
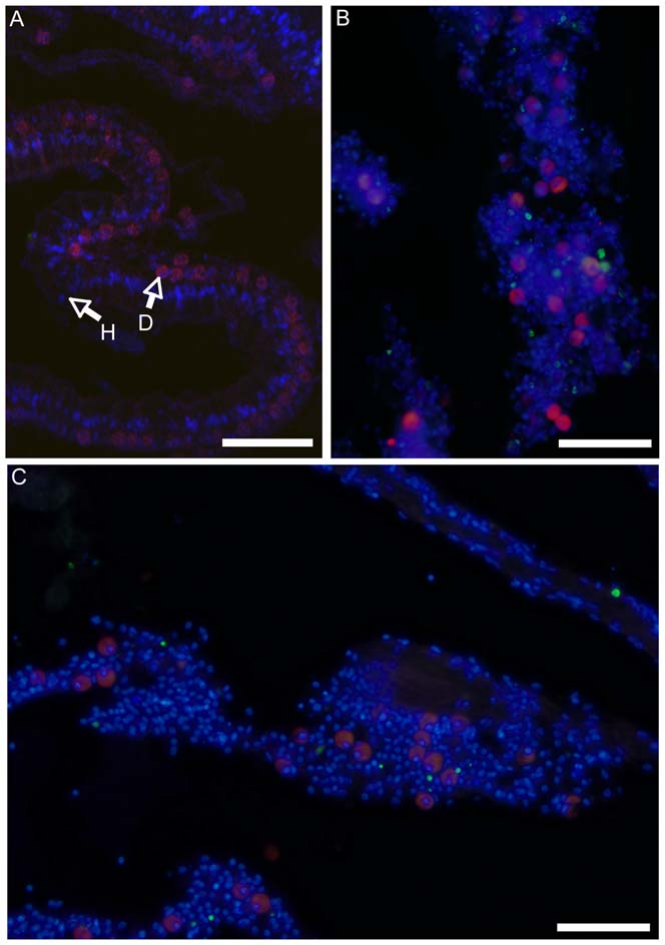
*In situ* detection of apoptosis in *Acropora millepora*. Apoptotic cells (green) stained via TUNEL assay in *A. millepora* incubated in control (A), heat stress (B) and colchicine (C) treatments. In red, the dinoflagellate cells are revealed by the autofluorescence of their chlorophyll content. In blue, the tissue of *A. millepora* is stained with DAPI. H = Host cell; D = Dinoflagellate cell. Scale bar = 50 µm.

**Figure 2:**
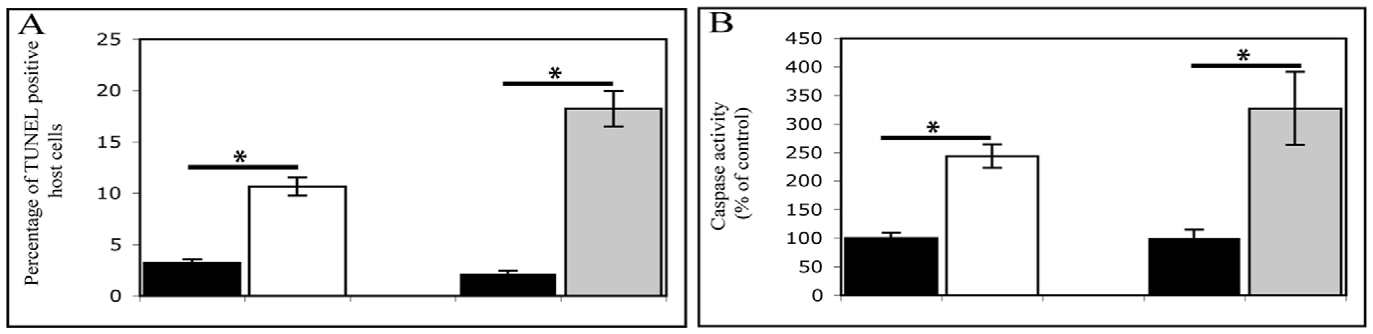
Quantification of apoptosis-like activity in *Acropora millepora*. Apoptosis-like activity measured as TUNEL positive cells and caspase 3-like activity within the host tissue of *A. millepora* incubated in control (black), colchicine (white) and fast heat stress (grey). (A) The percentage of TUNEL-positive host cells was measured in thin sections of coral tissues obtained from 6 coral branches (6 sections were analysed and averaged per coral branch), for each treatment and each time point. TUNEL-labelled host cells were counted in an image field (×40 magnification) and compared with the entire population of host cells in the same image field to derive the percentage of the TUNEL-positive population. A total of three image fields per tissue section was analysed and averaged. (B) The caspase 3-like activity assay was performed on *A. millepora* host tissue extract and is expressed as percentage of control. Error bars represent ± SEM, (N = 3 coral branches for caspase 3-like activity; N = 6 coral branches for TUNEL staining). * t- test in comparison to control, P<0.05.

The induction of apoptosis-like activity in coral tissue was confirmed by analysis of DNA fragmentation in coral tissue using fluorometric TUNEL assay (Figure 1). The percentage of host cells undergoing through cell death was quantified in sections of corals tissue by comparing the number of TUNEL- positive host cells to the total number of host cells in the same area. Only a subset of TUNEL-positive host cells were detected in the control samples (2 to 4%), while both the colchicine and fast heat stress treatments induced significant increase in TUNEL staining (11% of TUNEL positive host cells, 2.7-fold increase; t_10_ = −5.96, p<0.001 for colchicine and 18% of TUNEL positive host cells, 7.5-fold increase, t_10_ = −3.46, p = 0.006 for fast heat stress; Figure 2A). In order to study the effect of colchicine and fast heat stress treatments on caspase 3-like activity, Asp-Glu-Val-Asp (DEVD)-dependent protease activity was measured in the animal extract of *A. millepora*. In both treatments, the DEVD-dependent protease activity in the coral host tissue was significantly higher than in control (2.4-fold increase, t_4_ = 11.97, p<0.001 for colchicine and 3.3-fold increase, t_4_ = −6.16, p = 0.003 for fast heat stress; Figure 2B). To confirm the induction of a specific caspase 3-like activity, all measurements were also performed in the presence of 1 M DEVD-aldehyde. In these conditions, caspase 3-like activity was totally abolished. These observations provide evidence that incubation with either colchicine or fast heat stress induced increased caspase-dependent apoptosis in coral host tissue.

**Figure 3:**
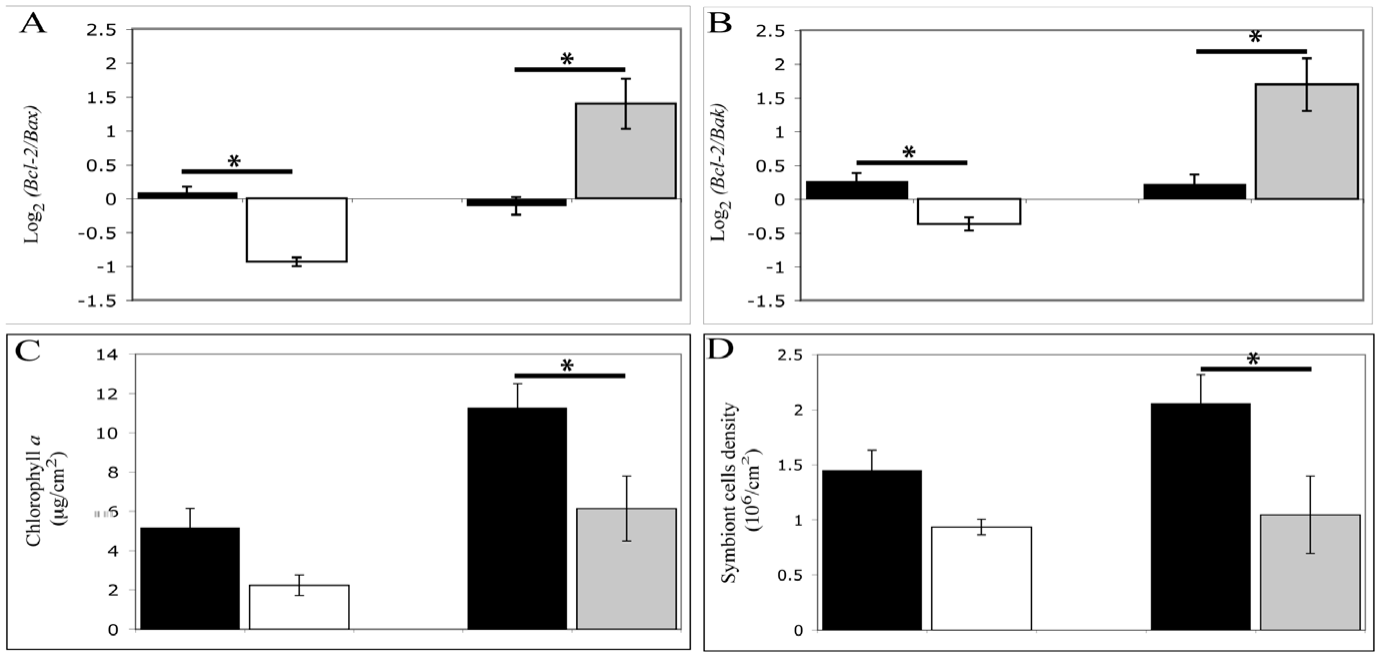
Differential gene expression of Bcl-2 family members and bleaching in *Acropora millepora*. (A) Log_2_ of Bcl-2 vs. Bax genes ratio, (B) log_2_ Bcl-2 vs. Bak genes ratio, (C) areal chlorophyll *a* concentration and (D) areal symbionts cells density in *A. millepora* incubated in control (black), colchicine (white) and fast heat stress (grey) treatments. Error bars represent ± SEM, (N = 6 coral branches). * t- test in comparison to control, P<0.05.

Differential expression of Bcl-2:Bax and Bcl-2:Bak ratios. A significant down-regulation of both Bcl-2:Bax (2-fold; t-test: t_10_ = 5.56, p<0.001) and Bcl-2:Bak ratios (1.5-fold; t_10_ = 2.82, p = 0.018) was observed for corals incubated in colchicine when compare to the controls. In contrast, rapidly heated samples showed a significant increase in Bcl-2:Bax (2.4-fold; t-test: t_10_ = −2.34, p = 0.041) and Bcl-2:Bak (2.9-fold; t_10_ = −4.77, p = <0.001) ratios in comparison to the control samples (Figure 3A and 3B).

#### Indicators of coral bleaching

In both colchicine and fast heat stress treated samples, similar changes in bleaching indicators were observed with a decrease in areal chlorophyll *a* concentration (2.3-fold and 1.8-fold decrease respectively) and areal symbiont density (1.6-fold and 2-fold decrease respectively) when compared to the controls (Figure 3C and 3D). However this decrease, whilst strong in both treatments, was only statistically significant for the stress associated with rapidly heated treatment (chlorophyll *a* concentration, t-test: t_10_ = 2.50, p = 0.031; areal symbiont density t-test: t_10_ = 2.31, p = 0.043).

### B. Effect of different thermal stress (slow and medium treatments)

**Figure 4:**
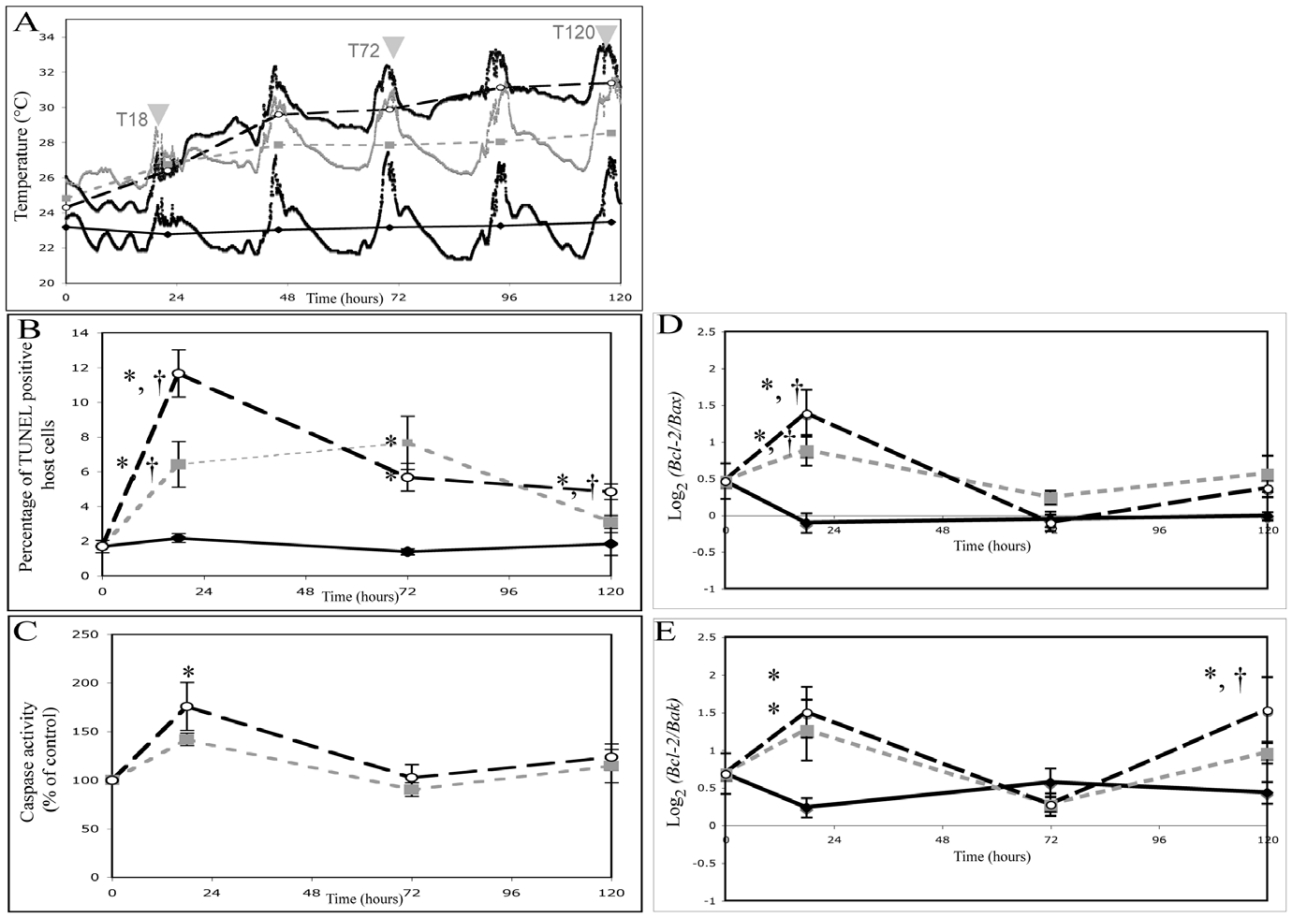
Effect of temperature on apoptotic mediators in *Acropora millepora*. (A) Temperature treatments for the duration of the experimental period (grey arrowheads indicate sampling points; dashed lines indicate daily average temperature; solid line indicate temperature recorded every 2 min). (B) percentage of TUNEL-positive host cells, (C) caspase 3-like activity expressed as percentage of control, (D) Log_2_ of Bcl-2 vs. Bax genes ratio and (E) Log_2_ of Bcl-2 vs. Bak genes ratio in *A. millepora* incubated in control (filled diamond), slow (grey square), and medium treatment (open circle). Error bars represent ± SEM, N = 3 coral branches for caspase 3-like activity and N = 6 coral branches for the other variables. *Significant difference (post-hoc LSD analysis, P<0.05) between treatment and control for the same time point. †Significant difference (post-hoc LSD analysis, P<0.05) between treatments for the same time point.

In the early stages of the experiment (i.e. 0 to 48 h), the slow and medium treatments displayed temperature increases in the range of natural bleaching conditions previously reported on the Great Barrier Reef [33]. For both the slow and medium treatments a plateau followed these temperature increases from 72 h onward (Figure 4A).

#### Apoptosis-like activity in host tissue

The proportion of host cells undergoing through DNA fragmentation was monitored by using TUNEL assay in the different treatments throughout the 120 h incubation. The percentage of TUNEL-positive host cells in the control samples was low (1.3 to 2.3% of the total number of host cells) and did not vary significantly throughout the experiment (one-way ANOVA F_3,24_ = 0.65; p = 0.59; Figure 4B). In contrast, there was a change in the proportion of TUNEL-positive cells among the different experimental treatments with a significant effect of the interaction between time and treatment (two-way ANOVA, time: F_3,54_ = 4.88, p = 0.004, treatment: F_3,54_ = 13.63, p<0.001, interaction: F_1,54_ = 125.88, p<0.001). Corals in both temperature treatments showed similar trends with a significant increase in the relative amount of TUNEL-positive host cells in the early sampling points (18 h sampling time, slow treatment: 6.4% of TUNEL positive host cells, 2.9-fold increase relative to control, Fischer LSD p = 0.015; medium treatment: 11.6% of TUNEL positive host cells, 5.2-fold increase relative to control, Fischer LSD p<0.05 Figure 4B). This increase in DNA fragmentation was restored to a level close to control in later steps of the experiment (i.e. 120 h, slow treatment no significant difference relative to control; medium treatment: 4.8% of TUNEL positive host cells, 2.1-fold increase relative to control, Fischer LSD p<0.05). The fluorometric assay of caspase 3-like activity indicated an increase in activity in the first 18 h for both slow and medium thermal treatments when compared to the control (1.4-fold and 1.7-fold increase, respectively; Figure 4C). However, this increase in DEVD-dependent protease activity, whilst strong in both treatments was only statistically significant for medium treatment compared to the control (Fischer LSD p<0.01 for medium treatment; p = 0.086 for slow treatment). After 72 h of incubation, caspase 3-like activities in both treatments were restored to control levels (Figure 4C).

#### Differential expression of Bcl-2:Bax and Bcl-2:Bak ratios

**Figure 5:**
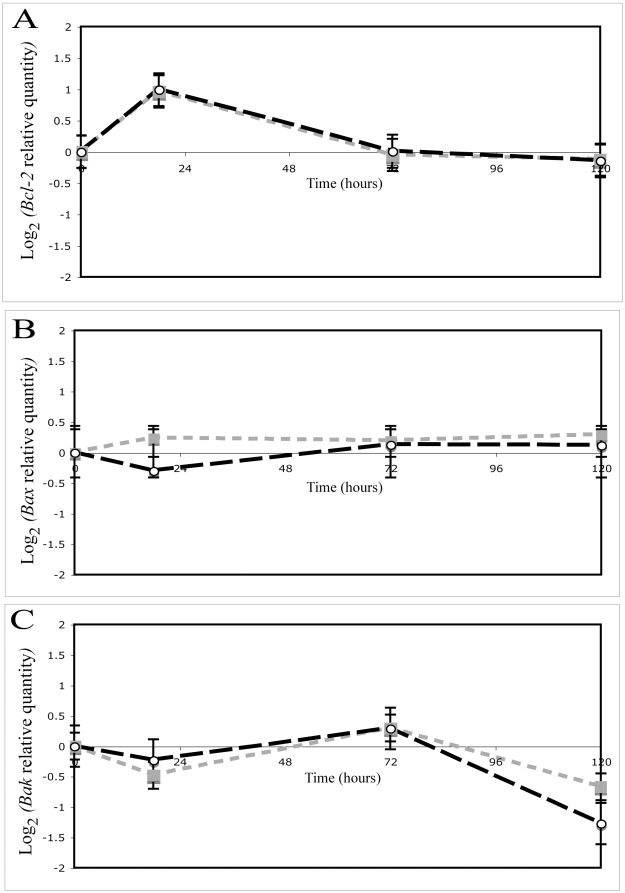
Effect of temperature on single gene expression of Bcl-2 family members in *Acropora millepora*. Differential expression of (A) Bcl-2, (B) Bax and (C) Bak genes in *Acropora millepora* incubated in slow (grey square), and medium treatment (open circle). Data is expressed by log_2_ Relative Quantification versus the control group. Error bars represent ± SEM, N = 6 coral branches.

There were no significant difference between samples taken at different time points from control conditions in both Bcl-2:Bax (one-way ANOVA F_3,24_ = 0.23, p = 0.87) and Bcl-2:Bak ratios (one-way ANOVA F_3,24_ = 0.80, p = 0.50). In contrast, there was a differential regulation of gene ratios with a significant effect of the interaction between time and treatment among the different experimental treatments for both Bcl-2:Bax (two-way ANOVA, time: F_2,54_ = 15.11, p<0.001, treatment: F_2,54_ = 6.08, p = 0.004, interaction: F_1,54_ = 310.42, p<0.001) and Bcl-2:Bak gene ratios (two-way ANOVA time: F_2,54_ = 8.32, p<0.001 treatment: F_2,54_ = 10.67, p<0.001,interaction: F_1,54_ = 422.36, p<0.001; Figure 4D and 4E). Furthermore, a significant effect of thermal treatment was revealed on both Bcl-2:Bax gene ratio (one-way ANOVA: F_2,18_ = 5.01, p = 0.009) and Bcl-2:Bak gene ratio (one-way ANOVA: F_2,18_ = 4.87, p = 0.011) in the first 18 h i.e. when the rate of temperature increase was the greatest (Figure 4A). In comparison to controls, corals removed from the hyperthermic treatments at 18 h had an increased level of expression for both Bcl-2:Bax gene ratio (slow treatment: 1.8 up-regulation, medium treatment: 2.6 up-regulation; Fischer LSD p<0.05 for both treatments Figure 4D) and Bcl-2:Bak gene ratio (slow treatment: 2.4 up-regulation, medium treatment: 2.8 up-regulation; Fischer LSD p<0.05 for both treatments, Figure 4E). At the last sampling point (i.e. 120 h), the Bcl-2:Bak gene ratio significantly increased in only the medium heat treated corals compared to controls (Fischer LSD p<0.05, Figure 4E). These changes observed in Bcl-2:Bax and Bcl-2:Bak ratios were driven by different regulations at a single gene level in the early and later steps of the experiment (Figure 5A, 5B and 5C).

#### Indicators of coral bleaching

**Figure 6:**
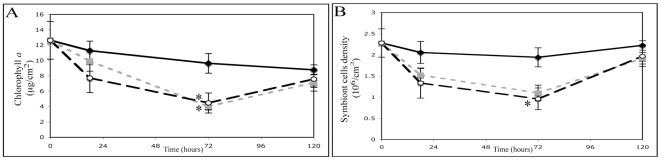
Effect of temperature on bleaching in *Acropora millepora*. Coral bleaching measured as (A) areal chlorophyll a concentration and (B) areal symbionts cells density in *A. millepora* incubated in control (filled diamond), slow (grey square), and medium treatment (open circle). Error bars represent ± SEM, N = 6 coral branches. *Significant difference (post-hoc LSD analysis, P<0.05) between treatment and control for the same time point. †Significant difference (post-hoc LSD analysis, P<0.05) between treatments for the same time point.

The areal concentration in chlorophyll *a* (µg per cm^2^) and the areal symbiont density (cells per cm^2^) remained constant in the control samples with no significant difference across the experiment (chlorophyll *a*: one-way ANOVA F_3,24_ = 1.70, p = 0.20; symbiont density: one-way ANOVA F_3,24_ = 1.26, p = 0.31) indicating that the maintenance of corals in aquaria in stable ambient temperature did not induce bleaching. In contrast, both the areal concentration of chlorophyll *a* and the areal population density of *Symbiodinium* among the different experimental treatments revealed a significant effect of the interaction between time and treatment with a similar decreasing trend within the first 72 h (Figure 6A, and 6B; symbiont density: two-way ANOVA, time: F_2,54_ = 6.25, p = 0.001, treatment: F_2,54_ = 5.19, p = 0.009, Interaction F_1,54_ = 397.3, p<0.001; chlorophyll *a*: two-way ANOVA time: F_2,54_ = 5.60, p = 0.002, treatment: F_2,54_ = 4.07, p = 0.022, Interaction F_1,54_ = 279.8, p<0.001). These results indicated that bleaching was characterised by a reduction in areal symbiont density and photosynthetic pigment that can be attributed to a loss or *in situ* degradation of *Symbiodinium* from the host.

In corals subjected to the slow and medium heat treatments, the onset of bleaching was observed after 18 h, but was only significant in comparison to control after 72 h of incubation with a decrease in the areal concentration of chlorophyll *a* for both treatments (Fischer LSD p<0.01 and p<0.05 respectively) and a decrease in the areal symbiont density (statistically significant for medium treatment only; Fischer LSD p<0.05). Coral branches removed from the treatments at 120 h showed a significant increase in the areal symbiont density in comparison to 72 h (slow treatment: t-test: t_10_ = −3.59, p<0.01; medium treatment: t-test: t_10_ = −2.85, p<0.05). As a result, the areal symbiont densities in the treated samples were not significantly different from the control samples at 120 h (one-way ANOVA: F_2,18_ = 0.89, p = 0.43; Figure 6B).

## Discussion

Deciphering the cellular and molecular mechanisms that determine the sensitivity of corals to thermal stress represents an important step to predicting and understanding the impacts of climate change on coral reefs. In this respect, apoptosis is an important and fundamental component of the response of corals to thermal stress. Indeed, several studies focusing either on functional activation of apoptosis or transcriptomics in Cnidaria have separately demonstrated the involvement of apoptosis in response to rising seawater temperature [2], [6], [9], [11], [34], [35]. Research that integrates these mechanisms into the spectrum of physiological changes involved in the heat stress response corals is limited. In the present study, we conducted such a multi-parameter analysis by monitoring (i) the potential initiation of the apoptotic pathway by differential expression of Bcl-2 members (Bcl-2, Bax and Bak), (ii) the induction of early apoptotic events by activation of caspase 3-like and (iii) the completion of apoptotic cascade by DNA fragmentation in the reef-building coral *Acropora millepora*. We have related these to the overall response of corals to its stress (i.e. coral bleaching) by monitoring a number of physiological parameters.

The application of colchicine or heat shock induced a significant increase in host cell death indicated by DNA fragmentation (2.7-fold more TUNEL-positive host cells for colchicine and 7.5-fold more TUNEL-positive host cells for exposed to rapidly heated conditions; Figure 2A). These data support previous studies in Cnidaria reporting the functional activation of cell death in response to either colchicine or heat shock [6], [15]. Interestingly, the increase in DNA fragmentation was stronger in coral host tissue after heat stress (7.5-fold increase) than after treatment with colchicine (2.7-fold increase) as previously reported by Dunn *et al* [15]. Further, the increase in TUNEL positive host cells was corroborated by an increase in caspase-3 like activity in both treatments (2.4-fold increase for colchicine and 3.3-fold increase for fast heat stress, Figure 2B), supporting that DNA fragmentation observed via TUNEL labelling was a result of rapid activation of caspase-dependent apoptosis. However, colchicine and fast heat stress treatments induced different outcomes regarding the differential expression of Bcl-2 family members. Indeed, Bcl-2:Bax and Bcl-2:Bak gene ratios were selectively down regulated in response to colchicine stimulus while they were selectively up-regulated in response heat shock. These results may be due to the different modes of operation in initiating the apoptotic cascade and need further investigation.

Colchicine induces cytoskeletal alterations that inhibit the completion of mitosis. In response to this stressor, the cell aborts mitosis and initiates the release of cytochrome *c* and the activation of caspase 3 leading to cell death [36]. In the present study, the relative down-regulation of Bcl-2 versus Bax or Bak observed in colchicine treated samples (Figure 3A and 3B) reflected a down-regulation of Bcl-2 at a single gene level (1.8-fold decrease; no significant change for Bax and Bak, data not shown) and was associated with the induction of caspase-dependent apoptosis and some, though no significant, bleaching (Figure 3C and 3D). These data suggest that the initiation of apoptosis by this chemical inducer may be mediated by the suppression of anti-apoptotic mechanism rather than an induction of pro-apoptotic mechanisms.

In contrast to the treatment with colchicine, the up-regulation of Bcl-2:Bax and Bcl-2:Bak gene ratios (Figure 3A and 3B) observed during coral response to heat shock suggests a more complex scenario. Indeed, previous studies with different models have demonstrated thermal induction of Bcl-2 expression [26] and the overexpression of Bcl-2 is generally described to promote resistance against cell death [24]. However, in our study the relative up-regulation of Bcl-2 was associated with increased caspase-dependent apoptosis. This contrasting situation could be interpreted by different explanations. Firstly, the relative quantification of Bcl-2 family members at a transcript level may not reflect their respective protein activity and the upstream initiation of apoptosis. Indeed, the anti-apoptotic Bcl-2 mechanism that has been best characterised during thermal stress involves direct binding to pro-apoptotic Bax and Bak and highly depends on post-translational modifications that may not be followed by differential gene expression [37]. Nevertheless, Bcl-2 also exerts anti-apoptotic effects that are mediated by its influence on mitochondrial redox capacity and are independent of its interaction with pro-apoptotic proteins. Previous studies in other models have shown that Bcl-2 protein up-regulation is accompanied by increased mRNA levels in response to oxidative stress [38], [39]. In this respect, we cannot exclude that the relative up-regulation in Bcl-2 transcript levels observed after heat shock maybe be related to an antioxidant response of coral host cells. A second explanation is that the relative up-regulation of Bcl-2 could take few hours to be active as an antioxidant/anti-apoptotic response because translation and accumulation of protein products naturally takes time. In the light of increased caspase activation under the same treatment, our results could be interpreted in a model where thermal stress engages simultaneously (i) activation of caspase-dependent apoptosis in the cells with irreparable damage and (ii) initiation of a concurrent antioxidant response including a relative overexpression of Bcl-2 that could have a delayed mitigating effect on apoptotic activity in the surviving cell population. Indeed, the regulation of apoptosis in coral tissue in response to heat stress ranges across two different scales. Firstly, the coral tissue is formed by 2 tissue layers: ectoderm and gastroderm (which contains the dinoflagellate symbionts). Whether or not a cell dies as a result of heat stress is dependent on the net balance between damage, protection and the capacity for repair. In symbiotic corals, both partners can mediate the resistance to heat stress. On the one hand, because compromised dinoflagellate symbionts are one of the first sites of ROS production and damages during coral bleaching [1], [4], the intra-population variability and occurrence of different clades or sub-clades of *Symbiodinium* exhibiting different tolerances to heat stress [5] can arguably have variable impact on the damage to coral host cells. On the other hand, different level of expression of antioxidant proteins such as Bcl-2 in coral host cells can lead to differential level of protection against oxidative stress induced by elevated temperature as it has been described in human and murine cell lines by Setroikromo *et al* [40]. Further, a second scale that has to be considered when measuring the regulation of apoptosis in response to heat stress is the time. Indeed, the different components of the apoptotic cascade are regulated in a time-dependent fashion (initiation, activation and completion). Therefore, in order to precise the effect of heat stress on the triggering and the execution of apoptosis in corals it is crucial to integrate the effect of time.

The dynamic of initiation, activation and completion of apoptotic cascade in corals incubated with the different thermal treatments support this model whereby early thermal stress engages activation of apoptosis and the onset of a delayed protective response in corals. Indeed, both thermal treatments indicated consistent activation of apoptosis in *A. millepora* with increase in DNA fragmentation and caspase 3-like activity in the early stages (18 h; Figure 4B and 4C) of the experiment but also relative up-regulation of Bcl-2 versus Bax and Bak (Figure 4D and 4E) corresponding to an overexpression of Bcl-2 at single gene level (1.9-fold increase for slow treatment, 2-fold increase for medium treatment; no significant change for Bax and Bak; Figure 5A, 5B and 5C). Furthermore, the apoptotic activity in the late stages of the experiment (i.e. after 72 h) started to decrease in the host tissue and was restored to level close to control with further treatment duration. These data support recent transcriptomic studies suggesting that heat stress engages different responses related to apoptosis and oxidative stress in corals [9], [34], [35], [41], [42]. The microarrays used by Desalvo *et al* [34] and Voolstra *et al* [35] did not contain any Bcl-2 family members but some of the differentially expressed genes were clearly suggestive of apoptosis (down-regulation of voltage-dependent anion-selective channel 2, calreticulin, and pro-apoptotic caspase adapter protein) as well as of protective response of corals to oxidative stress (up-regulation of catalase, peroxidasin-like protein and catalytic subunit of glutamate-cysteine ligase) [9]. At a functional level, these data also support previous works indicating activation of apoptosis and induction of cytoprotective effects against oxidative stress during heat stress in other Cnidaria [6], [7], [8], [9]. Interestingly, Richier *et al* [6] monitored both caspase and antioxidant activity in the gastroderm of *Anemonia viridis* during thermal stress and showed time-dependent patterns congruent with those observed in our study, i.e. increasing trends during the early steps of thermal treatment followed by significant decrease after 24 h for caspase 3-like activity and after 48 h for antioxidant activity. Although the present study included only 4 time points and did not address the production of ROS/antioxidant activity, the release of cytochrome *c* and mitochondrial integrity, we propose an anti-apoptotic role for Bcl-2 in *A. millepora* because: (i) the results in this study concur with previous studies in different models including a basal metazoan [26] indicating that thermal induction of Bcl-2 expression promotes resistance against cell death; (ii) the relative expression of Bcl-2 was downregulated in response to chemical induction of apoptosis in coral tissue by colchicine (iii) the relative up-regulation of Bcl-2 during heat stress was followed by a decrease in caspase-dependent apoptosis in the host tissue. It is already established that Bcl-2 can act as an antioxidant in other metazoans by increasing the expression of intracellular defences against ROS such as catalase and glutathione, by counteracting lipid peroxidation or by directly inhibiting mitochondrial ROS generation [39]. However, it remains to be determined if the relative up-regulation of Bcl-2 observed in our study is functionally connected to any antioxidant activity in *A. millepora* even slightly delayed in time. Future studies should therefore attempt to modulate the endogenous antioxidant response of the host (by adding exogenous antioxidants for instance) and monitor the differential expression of apoptotic mediators, the antioxidant response and the release of cytochrome *c* in the cytosol.

In the later stages of the experiment, thermal stress treatments also revealed a significant up-regulation of Bcl-2:Bak ratio between 72 h and 120 h (Figure 4E) mostly due to a decrease in the level of Bak transcripts (1.6-fold decrease for slow treatment, 2.4-fold decrease for medium treatment, Figure 5C) while the level of Bcl-2 and Bax remained stable (Figure 5A and 5B). The relative down-regulation of Bak was not directly related to detectable changes in apoptotic activity. However, because this differential expression occurred during the last sampling point, a potential delayed effect on apoptotic activity should not be ignored and requires further studies with even greater duration of treatments.

In symbiotic Cnidaria, thermal stress has been shown to lead to damage and subsequent initiation of cell death resulting in the breakdown of the cnidarian-dinoflagellate symbiosis (bleaching) [43]. Interestingly, we found that colchicine was able to activate caspase-dependent apoptosis in corals and a degree of bleaching. These results support an involvement of apoptosis in coral bleaching [2], [6], [10], although other pathways such as autophagy and necrosis have also been shown to play roles in symbiont release [3], [10]. Thermal stress induced bleaching was also associated with increased apoptotic activity in *A. millepora* samples. Indeed, the breakdown of the *A. millepora-Symbiodinium* symbiosis and the subsequent decrease in symbiont cell density were observed within the early stages of slow and medium treatments (Figure 6A and 6B), occurring when the percentage of TUNEL positive host cells and the caspase 3-like activity were the most important (Figure 4B and 4C) while coral branches removed from the treatments at 120 h showed a significant recovery in symbiont cells density and associated photosynthetic pigments. This recovery might either come from i) an uptake of exogenous stress-tolerant symbionts by corals or ii) a proliferation of endogenous stress-tolerant symbionts. Because this experiment was run in an open water system, it is difficult to exclude the first case. However, a recent study has suggested that the ability of scleractinian corals to acquire and maintain exogenous, stress-tolerant symbionts is limited [44]. Moreover, in our study, the ability of corals to regain dinoflagellate symbionts was fast (1.7-fold and 2-fold increase in symbiont cells density between 72 h and 120 h for slow and medium treatment respectively) and was therefore more probably related to a proliferation of more stress-tolerant symbionts initially present at low level within the host. This recovery of the population of symbiotic dinoflagellate indicates that the treatments used during the 120 h experiment represented non-lethal conditions. In natural conditions, mass bleaching events can lead to high mortality of corals [45]. Further studies on the regulation of apoptotic mediators in corals should therefore include greater duration experiments with more severe bleaching conditions. Interestingly, the late recovery in the symbiosis observed in our experiment was related to a reduction in apoptotic activity supporting a delayed onset of a protective response in the coral host that could require new proteins expression including some anti-apoptotic mediators such as Bcl-2. The variation in susceptibility to bleaching is commonly explained by the occurrence of different clades or sub-clades of *Symbiodinium* and potentially their variable tolerance to stress [4], [5]. There is also increasing evidence that coral host can influence the sensitivity of coral-dinoflagellate assemblage to heat stress by regulating a large arsenal of stress proteins that act to repair damage of oxidative stress [9], [35], [41], [46]. Overexpression of Bcl-2 could conceivably be one of these important antioxidant mechanisms in corals exposed to sudden environmental changes. In this respect, it is interesting to note that the heating rate explained more than 78% of the variation in Bcl2:Bax transcripts in *A. millepora* during our thermal stress experiments (Figure S1). This result suggests that the heating rate could be an important environmental driver of this molecular response in the host rather than duration and intensity of thermal anomalies [47]. However, because the thermal treatments used in this study varied in both the average temperature and the heating rate (Figure 4A), the effects observed on the level of expression of Bcl-2 family members are attributable to some combination of these 2 factors. Future studies should therefore include thermal treatments with tight control of temperature in order to precise the relative contributions of each factor (average temperature or heating rate) on the molecular regulation of apoptosis in corals.

This study provides further evidence that corals can regulate apoptosis in response to thermal stress. Bleaching is nevertheless an extremely dynamic event with many different triggers, responses and consequences to coral-dinoflagellate symbiosis. Understanding the cellular dynamics and the mechanisms driving the breakdown of this symbiosis will require many further studies but this is a critical step to predict future bleaching events and coral mortality under a changing climate.

### Conclusion

In conclusion, our study contributes to the characterization of the apoptotic pathways and their functional activation in symbiotic Cnidaria during thermal stress. Our data show rapid activation of caspase-dependent apoptosis in response to different stimuli (colchicine and heat stress) in the reef-building coral *Acropoa millepora*. In regards to the molecular pathway, the anti-apoptotic function of Bcl-2 is suggested in this model by: (i) Bcl-2 down-regulation in response to chemical induction of apoptosis and (ii) Bcl-2 up-regulation followed by a decrease in caspase-dependent apoptosis during heat stress. The results of this study therefore support a model of heat stress in reef-building corals whereby increasing temperatures activates caspase-dependent apoptosis in irreparable cells and the onset of a delayed protective response involving overexpression of Bcl-2 in surviving cells.

## Materials and Methods

### A. Collection and maintenance of corals

*Acropora millepora* corals used in this study were collected on the reef flat adjacent to Heron Island Research Station (HIRS, 23°33′S, 151°54′E) in May 2008. Four healthy colonies of *A. millepora* were divided into single upward-growing branch tips (6–8 cm long) by using wire cutters and transported immediately to the seawater facility at HIRS. Ten branches of each colony were randomly placed in each of the 12 outdoor aquariums (60 l, 3 tank replicates per temperature treatment) with running sea water (40 coral branches per aquarium).

### B. Experimental treatments

#### Thermal stress

Coral branches were acclimated 48 h at the winter mean local ambient temperature (23–25°C) and at the ambient light levels, which were adjusted using shade cloth to mimic the conditions of the natural reef flat. To explore the influence of temperature, the systems were then subjected to the following experimental temperature regimes: slow (1°C increase per approx 12 h; 23–28±1°C), medium (1°C increase per approx 10 h 23–30±1°C) and a control group (stable at 23–24±1°C; ambient temperature). A substantial hyperthermic stress (fast heat stress; 1°C increase per approx 2.5 h from 24–32±1°C in 18 h) was also used to ensure that bleaching occurred in *A. millepora*. Corals branches were then exposed to their experimental temperature regimes during 18 h for the fast heat stress and during 120 h for the control, slow and medium temperature regimes. Water temperature was recorded every 2· min using StowAway TidbiT Loggers placed in each aquarium (Onset Computer Corporation, Bourne, MA, USA). A subset of coral branches (N = 6) was randomly removed from the treatments and control tanks at 0, 18, 72, and 120 h. To avoid any bias and potential tissue lesions due to fragmentation process, for each sample, a small basal portion of the coral branch (1 cm) was removed using wire cutters. The rest of the coral branch was then divided into 3; one part was directly snap frozen using liquid nitrogen and stored at −80°C for RNA extraction, a second part was also directly snap frozen using liquid nitrogen and stored at −80°C for pigment and symbiont density analysis, and a further part was fixed in 4% paraformaldehyde in sterile seawater for later *in situ* analyses (TUNEL fluorometric assay).

#### Apoptosis induction by colchicines

The mitotic inhibitor, colchicine was used in accordance with previous studies on *C*nidaria to serve as a positive control for induction of apoptosis [13], [15]. Coral branches were individually placed in small containers (200 ml) containing 0.05% colchicine solution in artificial seawater for 18 h. A control group of coral branches was incubated in similar conditions with seawater only. For each treatments (colchicine and control), six coral branches (N = 6) were randomly removed at 0 and 18 h and processed as described above.

### C. Quantitative Real-Time PCR (qPCR)

The present study conforms to the Minimum Information for Publication of Quantitative Real-Time PCR guidelines [48]. In this section, we indicate the essential information, *sensu* [48], required to allow reliable interpretation of the corresponding qPCR results.

#### Primer design and RNA extraction for Acropora millepora apoptotic genes

Homologs of core components of Bcl-2 family have been previously characterized in the reef-building corals *Acropora aspera* and *A. millepora* [11]. An additional in-depth analysis of an annotated database generated by 454 sequencing of *Acropora millepora* larvae transcriptome [31] (http://www.bio.utexas.edu/research/matz_lab/matzlab/454.html), revealed identical transcripts encoding proteins with high similarities to the domains of Bcl-2 proteins including Bcl-2; Bax and Bak. These partial sequences were used as a template to design sequence-specific primers using the software, Primer3 0.4.0 [49] (Source code available at http://fokker.wi.mit.edu/primer3/; Table 1). Primer specificity to host genes was tested and confirmed by using aposymbiotic cDNA from *A. millepora* larvae (given by Mauricio Rodriguez-Lanetty) and sequence identity (*A. millepora* sequences showed no match identity with plant or dinoflagellate when compared against the other sequences found in the databases by using BLAST [50], Figure S2).

**Table 1:** Internal control genes and Bcl-2 family members investigated in *Acropora millepora* by using qRT-PCR.

Name	Accession number	Primer sequence 5′-3′	Primer sequence 3′-5′	Length (bp)	Tm
Beta-Actin	Levy *et al*, 2007	CGGACAGGTC ATCACCATTG	GGAACGATGG CTGGAATAGG	68	61.7
EF1-alpha	DY580404	CCCAAAACTG TGGCTTTTGT	TGCGTCGATA AGTGTCTTGC	141	60
Calmodulin	EZ030237	AGGTTGACCT GCTCGTGAGT	GCTGATGCAC TGATTGGTGT	104	59.7
Adeno-HomoCyase	Levy *et al*, 2007	CCTTGGATGTG CTATGGGTCA	GCCAAGACCT GGTTGGTGAA	66	63.1
L12	EZ024706	CACTGGTGGT GAAGTTGGTG	TCCAGTCTTGTGTTGCCTTG	110	59.9
L13	EZ040625	TTACTGGGCC GTTTAGCATC	GAGCACGGAA ATGAAATGGT	184	59.9
P0	EZ028666	GAAACGTGGG CTTTGTGTTT	TTAGTTGGAATGGCCAAAGC	188	60
Bcl-2	EU161957/EZ011917	AACTGGGGTCGCATTGTTG	AACTGAATAAA CCCTTCCCATCC	179	61.5
Bak	EZ037140	CTGGAATAAA CTGGGGACGA	CCGAAAATCA AGCACTCCTC	193	59.8
Bax	EU161958/EZ034459	TTCAGTGATG GCGTGGTAAA	CCCATCCTCC TTGTTCAAAA	172	59.9

Accession numbers of the sequences used as template for primer design, primers sequences, amplicon length and melting temperature are indicated. Primers for Beta-Actin and AdenoHomoCyase were previously described by Levy *et al* [62].

Corals samples were immediately snap frozen in liquid nitrogen, stored at −80°C for a maximum period of 1 month and ground in liquid nitrogen to a fine powder using sterile pestle and mortar. The resulting powder was transferred to a tube containing 700 ml of RLT buffer with beta-mercaptoethanol (RNeasy kit, Invitrogen), homogenised with a hand homogenizer (tissue tearor, Biospec products, inc) and centrifuged 3 min at 13000 g at 4°C. Total RNA was then extracted from the aqueous phase with RNeasy kit (Invitrogen) according to manufacturer's instructions and checked for quantity and integrity using an Agilent 2100 bioanalyzer. A total of 500 ng of high-quality total RNA (integrity number >7) for each sample in triplicate, was used for cDNA synthesis using the QuantiTect Reverse Transcription Kit (Qiagen) including a DNase step to remove contaminating genomic DNA according to manufacturer's instructions. Sample triplicates were pooled to minimize variation in the reverse transcription efficiency and for further Quantitative Real-Time PCR analysis.

#### Differential gene expression of Bcl-2 family members

Real-Time PCR assays were set up using an Eppendorf epMotion 5075 Robotics System (Eppendorf). Efficiency of target amplification was optimised prior to running samples for each primer pairs by trialling four primer concentrations (400, 200, 100 and 50 nM). Constant CT values for each primer set were observed at a [100 nM] final primer concentration for each of the primer pairs and we verified that the dissociation curve yielded a single peak indicating specific amplification of the target amplicon. Triplicate cDNA aliquots from each sample served as templates for qRT-PCR using SYBR Green PCR Master Mix (Applied Biosystems) on an Applied Biosystems 7900 Real-Time PCR System. Amplification of 10 µl reactions with 20 ng of cDNA from treated and control corals, and 100 nM of each specific primers were placed in 384-well optical plate (Perkin Elmer/Applied Biosystems Divisions) in the following conditions: incubation at 50°C for 2 min, then at 95°C for 10 min, 45 cycles of 95°C for 15 sec and 60°C for 1 min. Real-time PCR efficiency for each gene and each treatment were determined from a cDNA dilution gradient of 243, 81, 27, 9 and 3 ng and a linear regression model [51]. The corresponding real-time PCR efficiencies were calculated according to the equation described by Radonic *et al* [52]:
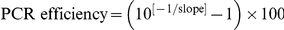



All qRT-PCRs displayed efficiencies between 95% and 102% (r^2^ of calibration curve >0.98). A no template control as well as a no reverse transcription control was generated for each gene and each treatment to ensure that the cDNA samples were free of DNA contamination.

#### Data acquisition

Data from qRT-PCR were analysed using the Sequence Detection Software (SDS 2.2). Expression levels were determined as the number of cycles needed for the amplification to reach a fixed threshold in the exponential phase of PCR reaction [53]. The cycle threshold (CT) was set at 0.01 for all genes, and corresponding CT values were transformed into quantities using PCR efficiency according to Vandesompele *et al* [54] in order to use geNorm software (http://allserv.ugent.be/jvdesomp/genorm/index.html).

#### Selection and normalisation of housekeeping genes (HKG)

The initial pool of potential reference genes used in this study was obtained previous studies [55], [56] (see Table 1). In order to select the best HKGs for the experimental conditions, expression stability was analyzed using GeNorm software [54] (http://allserv.ugent.be/jvdesomp/genorm/index.html). Under our experimental conditions, the most stable expression was revealed for genes coding for ribosomal proteins L12 and L13 in the colchicine treatment (M value = 0.241 Figure S3A) and for ribosomal proteins P0 and L13 in the different thermal treatments (M value = 0.485; Figure S3B). A minimum of 2 reference genes (L12 and L13) was recommended for accurate normalization of gene expression in the samples incubated with colchicine (V2/3 = 0.112; Figure S3C) while a minimum of 3 genes (P0, L13 and L12) were recommended in the samples incubated with thermal treatments (V3/4 = 0.127; Figure S3C). However, RTQPCR protocols cannot be used accurately with symbiotic organisms unless (i) all primer pairs show equal or no affinities for symbiont-derived cDNA and (ii) the relative contribution of each symbiotic compartment to the total nucleic acid pool is similar among the different samples [57]. In the case of bleaching experiment, it is clear that the differential symbiont densities harboured by stressed and control samples would potentially induce bias in the assessment of differential gene expression. Therefore, in order to interpret the gene expression data reliably and robustly, specificity to coral host genes was first confirmed by using aposymbiotic cDNA and sequence identity (BLAST) in order to avoid any affinities of the primers for symbiont derived cDNA. The ratios between Bcl-2:Bax and Bcl-2:Bak were then used as indicator of molecular regulation of cell death. Indeed, by using these gene ratios, a host target gene is directly compared to another host target gene within the same sample removing the potential bias induced by different symbiont densities. Furthermore, Bcl-2:Bax and Bcl-2:Bak ratios are generally known to dictate the relative sensitivity or resistance of cells to a wide variety of apoptotic stimuli [21] and they have been extensively used as prognostic markers to study cell death regulation [24].

### D. Detection of apoptosis *in situ*

#### Tissue preparation

Coral branches were fixed for *in situ* analysis in 4% paraformaldehyde in sterile seawater at 4°C for up to 8 h and embedded in 1.5% agarose prior to decalcification [58]. Coral fragments were decalcified using 20% (w/v) EDTA, followed by two washes in 1X phosphate buffered saline (PBS: 10 mM sodium phosphate and 130 mM NaCl). Samples were then stored at −20°C in a mixture of 0.5X phosphate buffered saline and 50% ethanol. Fixed tissues were dehydrated in an increasing series of ethanol and xylene and embedded in paraffin. Histological sections (10 µm) were collected on gelatin-coated slides. Paraffin was removed from the sections with xylene (three 10 min treatments), and the sections were rehydrated in a decreasing ethanol series.

#### TUNEL Assay

The protocol for identification of fragmented DNA as an indicator for late phase apoptotic cell death using Terminal Deoxynucleotide Transferase (TDT)–mediated dUTP nick end flourometric labelling (TUNEL) was adapted from the DeadEnd cell death detection kit manufactures instructions (DeadEndTM, Promega, Madison, WI, USA) as described previously [15]. Slides were rinsed in two successive PBS rinses for 5 min each. The PBS was removed and replaced by 80 µl of equilibration buffer provided for 15 min. The equilibration buffer was replaced by an rTdT enzyme/labeled nucleotides/buffer in accordance with the manufacturer's instructions and incubated in the dark at 37°C for 1 h. The TUNEL mix was later removed and slides were rinsed in PBS and counterstained with Hoechst 33342 (Molecular Probes, Invitrogen Corporation, Carlsbad, CA, USA) for 1 h. As a positive control, a selection of slides were treated with DNase I (not shown), and as a negative control (not shown), corals samples were treated with the TUNEL mix without the rTdT enzyme as suggested by the manufacturer. The slides were then rinsed in PBS. Coverslips were placed over tissue sections following addition of a 70% glycerol/PBS, pH 8, mountant solution. Slides were viewed and images taken on an LSM 510 metahead laser confocal microscope (Carl Zeiss AG,Oberkochen, Germany).

#### Computer assisted cell death count

The cell death quantification was determined on images of tissue sections using UTHSCSA Image Tool program (developed at the University of Texas Health Science Center, San Antonio, Tex., USA and available from the Internet by anonymous FTP from maxrad6.uthscsa.edu). DAPI and TUNEL-stained sections were visually inspected for overlapping nuclei within the gastrodermal coral tissue in different areas containing similar number of symbiotic dinoflagellates. Images were taken on laser confocal microscope and binary threshold images were produced that displayed the nuclei in white and the rest of the image in black. The find objects feature within the analysis tool was then used to automatically count the number of host cells in the DAPI and TUNEL stained section. For each treatment and each time point, TUNEL-labelled nuclei were counted in coral sections obtained from 6 coral branches (6 sections were analysed and averaged per coral branch), using the same magnification (x40) and compared to the total number of host cells in the same image field to obtain a percentage of TUNEL-positive host cells.

#### Caspase-like activity assay

Caspase 3-like activities were assayed fluorometrically (ApoAlert Caspase 3 fluorescent assay kit, BD Bioscience Clontech) using the specific substrate Asp-Glu-Val-Asp-7-amino-4-trifluomethylcoumarin (DEVD-AFC) following a protocol adapted from Dunn et al, [32]. Briefly, coral tissue from frozen coral branches was removed using an airbrush and filtered (0.45 µm) seawater solution. After separation of *Symbiodinium* sp. cells by centrifugation at 4500·g (4°C) for 5·min, 50 µl of supernatant containing coral homogenate was directly used according to the manufacturer's instructions. Caspase activity was measured in units of fluorescence (FU) per hour, using a SpectraMAX Gemini XS fluorometer and SOFTMax software (ver. 2.34, Molecular Devices) according to a standard curve obtained from AFC and the following equation:
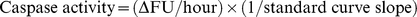



Where ΔFU/hour is the difference in FU between a coral homogenate and a negative control generated by adding caspase-3 inhibitor (DEVD-aldehyde) to coral homogenate. The total protein concentration of each sample was measured using the Bradford colorimetric method [59], a Bovine Serum Albumin protein standard (Sigma), and a Vmax Kinetic spectrophotometer microplate reader and SOFTMax software (ver. 2.34, Molecular Devices). Caspase activity was expressed as FU per hour per mg of coral protein.

### E. Indicators of coral bleaching

#### Cell densities of *Symbiodinium*

In order to assess dinoflagellate density in *A. millepora*, 6 coral branches were used for each treatment and each time point. Coral tissue from frozen coral branches was removed using an airbrush and filtered (0.45 µm) seawater solution. *Symbiodinium* sp. cells were separated from coral tissue by centrifugation at 4500·g (4°C); the pellet containing *Symbiodinium* sp. cells was then resuspended in filtered seawater solution (dilution 1:100) and used for direct symbionts cell counts. Symbionts cells density was determined using a SEDGEWICK rafter cell 550 haemocytometer (ProSciTech S8050, Kirwin, Queensland, Australia). For each coral branch, eight subsamples were counted and averaged. A total of twenty-five 1 µl cells were counted per subsample. Dinoflagellate density was then normalized to the surface area of each coral branch by using the melted paraffin technique [60].

#### Extraction of non water-soluble pigment

For each treatment and each time point, 6 coral branches were used to assess chlorophyll *a* areal concentration in *A. millepora*. Coral tissue from frozen coral branches was removed using an airbrush and *Symbiodinium* sp. cells were separated as described above. chlorophyll *a* was extracted and analysed in accordance with the methods reported by Middlebrook *et al* [61] using a SHIMADZU (Tokyo, Japan) SCL-10 HPLC attached to a SHIMADZU SPD-M10A photodiode array detector. Chlorophyll *a* content was then normalized to the surface area of each coral branch by using the melted paraffin technique [60].

### F. Statistical analysis

Statistical analyses were done using the software Statistica 7.0 (Statsoft Inc., Tulsa, OK, USA). Kolmogorov-Smirnov and Levene'test first tested the data for normality and homoscedasticity respectively. When the data deviated from normality and/or were not homogenous, then non-parametric tests (Kruskall–Wallis one way analysis) were applied instead of the parametric test (ANOVA and paired two-tailed t-test). Significant effects were analyzed using post hoc Fisher's Least Significant Difference (LSD) tests. Throughout the paper, values given are the mean of 3 coral branches ± Standard Error of the Mean (SEM) for caspase 3-like activity and the mean of 6 coral branches ± Standard Error of the Mean (SEM) for the other variables. Results were considered significant at the 5%. RT-PCR data are presented on a log2 scale in order to produce similar visual appearance for up and down-regulations.

## Supporting Information

Figure S1 **Relationship between Bcl-2:Bax gene ratio and the rate of temperature increase.** Effect of the rate of temperature increase on Bcl-2:Bax gene ratio at the different time points of the experiment (T18: 18 h; T72: 72 h; T120: 120 h) for Control (filled diamond), Slow (grey square), and Medium treatment (open circle). The rate of temperature increase was measured within the 24 hours preceding the other sampling times. The linear model provided a good fit, the rate of temperature increase explaining 78% of the variation in Bcl-2:Bax gene ratio for Acropora millepora (R-squared value = 0.783; P-value = 0.0015). Statistical significance was checked by an F-test of the overall fit. The dashed lines indicated 95% confidence interval.
(TIF)

Figure S2 **Taxonomy BLAST reports of Acropora millepora sequences.** Classification of the organisms found in the BLAST hitlist [50] for the different A. millepora sequences used for RT-PCR in this study: L12 (EZ024706); L13 (EZ040625); P0 (EZ028666); Bcl-2 (EZ011917); Bak (EZ037140), and Bax (EZ034459).
(PDF)

Figure S3 **Selection and normalisation of housekeeping genes in Acropora millepora.** Average expression stability values of reference genes in: (A) Heat stress and (B) Colchicine treatments. (C) Determination of the optimal number of reference genes for normalization by geNorm analysis [54].
(TIF)
