# Supplementary figures and images for: Regulation of Apoptotic Mediators Reveals Dynamic Responses to Thermal Stress in the Reef Building Coral Acropora millepora
Source: PLoS One. 2011 Jan 24;6(1):e16095. doi: 10.1371/journal.pone.0016095 (PMC3025915; doi:10.1371/journal.pone.0016095)

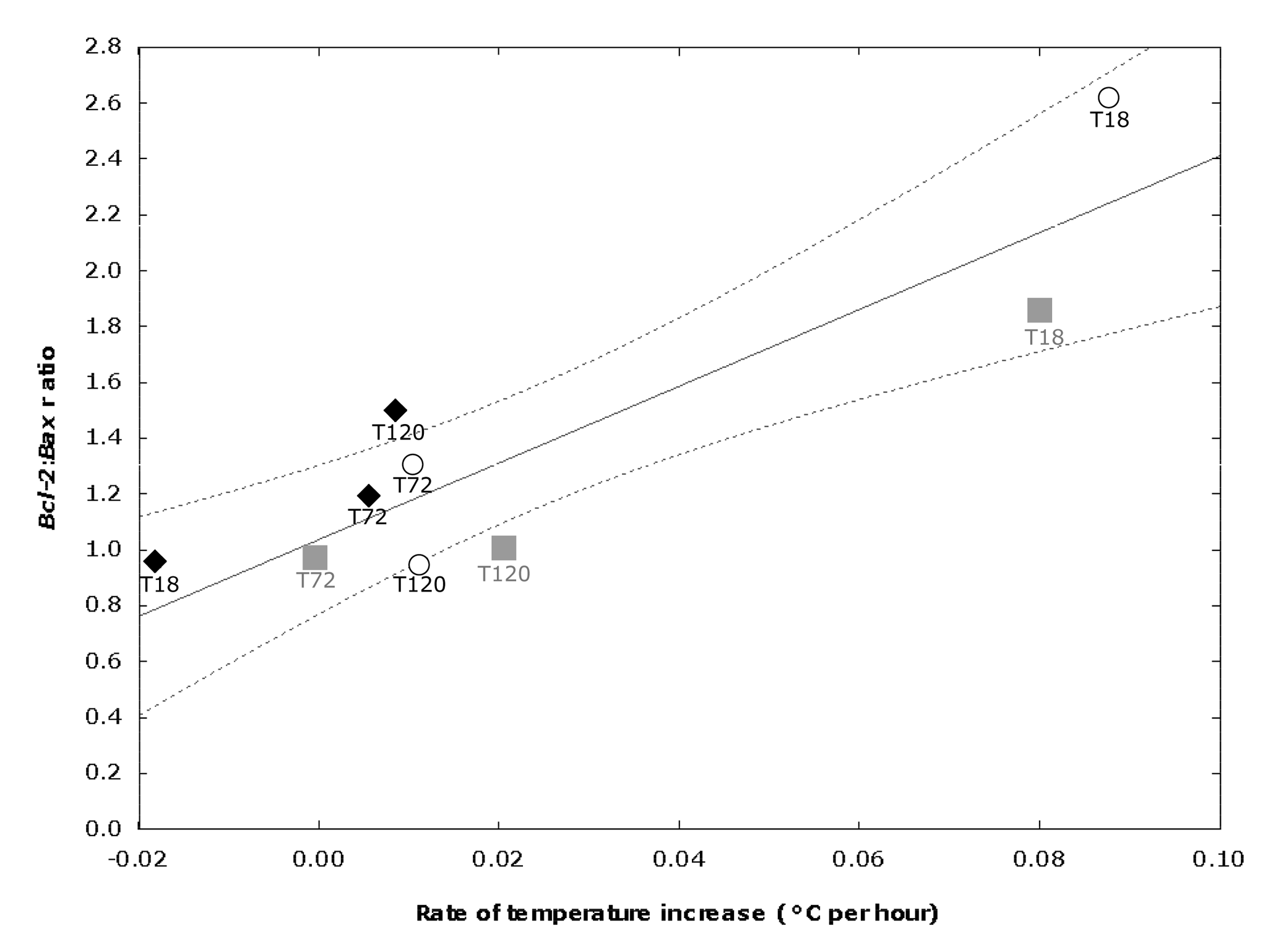

Supplement: Figure S1 — Relationship between Bcl-2:Bax gene ratio and the rate of temperature increase. Effect of the rate of temperature increase on Bcl-2:Bax gene ratio at the different time points of the experiment (T18: 18 h; T72: 72 h; T120: 120 h) for Control (filled diamond), Slow (grey square), and Medium treatment (open circle). The rate of temperature increase was measured within the 24 hours preceding the other sampling times. The linear model provided a good fit, the rate of temperature increase explaining 78% of the variation in Bcl-2:Bax gene ratio for Acropora millepora (R-squared value = 0.783; P-value = 0.0015). Statistical significance was checked by an F-test of the overall fit. The dashed lines indicated 95% confidence interval. (TIF) [file pone.0016095.s001.tif]

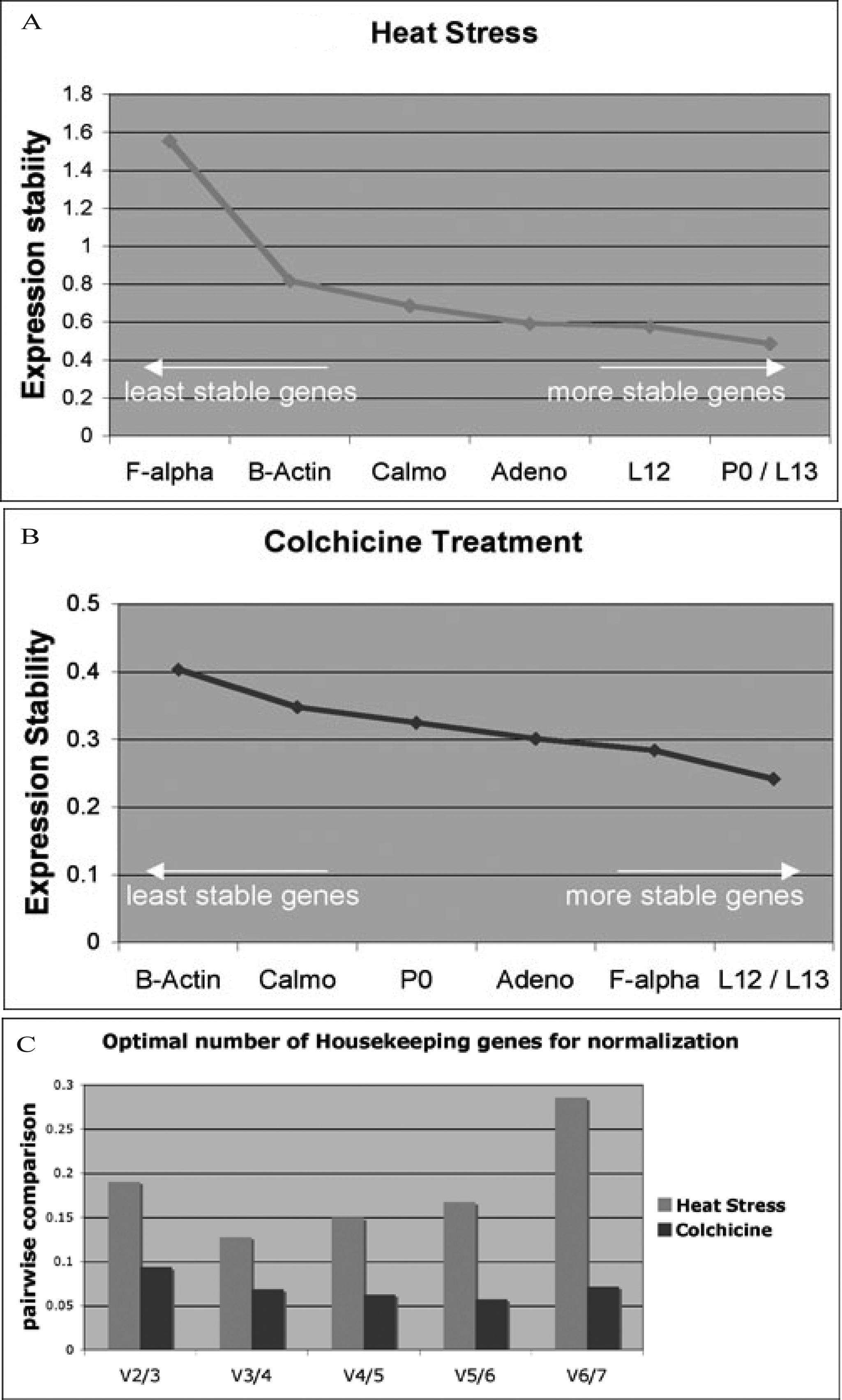

Supplement: Figure S3 — Selection and normalisation of housekeeping genes in Acropora millepora. Average expression stability values of reference genes in: (A) Heat stress and (B) Colchicine treatments. (C) Determination of the optimal number of reference genes for normalization by geNorm analysis [54]. (TIF) [file pone.0016095.s003.tif]
