# Supplementary material for: Regulation of Apoptotic Mediators Reveals Dynamic Responses to Thermal Stress in the Reef Building Coral Acropora millepora
Source: PLoS One. 2011 Jan 24;6(1):e16095. doi: 10.1371/journal.pone.0016095 (PMC3025915; doi:10.1371/journal.pone.0016095)
Supplement: Figure S2 — Taxonomy BLAST reports of Acropora millepora sequences. Classification of the organisms found in the BLAST hitlist [50] for the different A. millepora sequences used for RT-PCR in this study: L12 (EZ024706); L13 (EZ040625); P0 (EZ028666); Bcl-2 (EZ011917); Bak (EZ037140), and Bax (EZ034459). (PDF) [file pone.0016095.s002.pdf]

**BLAST**

# Basic Local Alignment Search Tool

Jump to Page Content

**Job Title: EZ024706**

---

- 

## Tax BLAST Report

**Index**

- Lineage Report
- Organism Report
- Taxonomy Report
- Help

### Lineage Report

```
root
. Fungi/Metazoa group [eukaryotes]
. . Metazoa              [animals]
. . . Eumetazoa              [animals]
. . . . Hexacorallia             [anthozoans]
. . . . . Scleractinia               [stony corals]
. . . . . . Acropora millepora ----------------------------        1 hit  [stony corals]    TSA: Acropora millepora SeqIndex4149, mRNA sequence
. . . . . . Montastraea franksi ...........................        1 hit  [stony corals]    Montastraea franksi ribosomal protein L12 (RibpL12) mRN
. . . . . Anthopleura elegantissima -----------------------        1 hit  [sea anemones]    Anthopleura elegantissima ribosomal protein L12 mRNA, c
. . . . . Nematostella vectensis ..........................        1 hit  [sea anemones]    Nematostella vectensis predicted protein (NEMVEDRAFT_v1
. . . . Microcosmus squamiger -----------------------------        1 hit  [tunicates]       Microcosmus squamiger partial mRNA for ribosomal protei
. . . . Branchiostoma floridae ............................        1 hit  [lancelets]       Branchiostoma floridae hypothetical protein, mRNA
. . . . Pediculus humanus corporis (human body lice) ......        1 hit  [lice]            Pediculus humanus corporis 60S ribosomal protein L12, p
. . . . Patiria miniata ...................................        1 hit  [starfish]        TSA: Patiria miniata isotig10042.Pminbgast mRNA sequenc
. . . . Phragmatopoma lapidosa ............................        1 hit  [segmented worms] Phragmatopoma lapidosa ribosomal-like protein mRNA, com
. . . . Maconellicoccus hirsutus (pink hibiscus mealybug) .        1 hit  [scale insects]   Maconellicoccus hirsutus clone WHMH4004 putative riboso
. . . . Nasonia vitripennis ...............................        1 hit  [wasps &c.]       PREDICTED: Nasonia vitripennis similar to ribosomal pro
. . . . Solea senegalensis ................................        1 hit  [bony fishes]     Solea senegalensis RPL12 mRNA for ribosomal protein L12
. . . . Ornithodoros parkeri ..............................        1 hit  [mites & ticks]   Ornithodoros parkeri clone OP-85 40S ribosomal protein
. . . . Ixodes scapularis (shoulder tick) .................        2 hits [mites & ticks]   Ixodes scapularis ribosomal protein L12, putative, mRNA
. . . . Dermacentor variabilis ............................        1 hit  [mites & ticks]   Dermacentor variabilis isolate DvM 40 ribosomal protein
. . . . Acyrthosiphon pisum ...............................        5 hits [aphids]          TSA: Acyrthosiphon pisum strain LSR1 Contig 60495.Acpii
. . . . Hypophthalmichthys nobilis (noble carp) ...........        1 hit  [bony fishes]     Hypophthalmichthys nobilis ribosomal protein L12 mRNA,
. . . . Salmo salar .......................................        3 hits [bony fishes]     Salmo salar clone ssal-plnb-020-145 60S ribosomal prote
. . . . Rhipicephalus sanguineus ..........................        1 hit  [mites & ticks]   TSA: Rhipicephalus sanguineus RS-401 mRNA sequence
. . . . Tribolium castaneum (rust-red flour beetle) .......        1 hit  [beetles]         PREDICTED: Tribolium castaneum similar to ribosomal pro
. . . . Eurythoe complanata ...............................        1 hit  [segmented worms] Eurythoe complanata ribosomal protein rpl12 mRNA, compl
. . . . Phoronis muelleri .................................        1 hit  [phoronid worms]  Phoronis muelleri putative 60S ribosomal protein RPL12
. . . . Anopheles gambiae str. PEST .......................        1 hit  [flies]           Anopheles gambiae str. PEST AGAP010065-PA (AgaP_AGAP010
. . . . Anopheles gambiae .................................        2 hits [flies]           Single read from an extremity of a full-length cDNA clo
. . . . Timarcha balearica ................................        1 hit  [beetles]         Timarcha balearica mRNA for ribosomal protein L12e (rpL
. . . . Rhagoletis pomonella ..............................        1 hit  [flies]           TSA: Rhagoletis pomonella contig09541, mRNA sequence
. . . . Saccoglossus kowalevskii ..........................        1 hit  [hemichordates]   PREDICTED: Saccoglossus kowalevskii ribosomal protein L
. . . . Ciona intestinalis ................................        2 hits [tunicates]       PREDICTED: Ciona intestinalis similar to predicted prot
. . . . Macaca mulatta (rhesus macaque) ...................        5 hits [primates]        PREDICTED: Macaca mulatta 60S ribosomal protein L12-lik
. . . . Scarabaeus laticollis .............................        1 hit  [beetles]         Scarabaeus laticollis mRNA for ribosomal protein L12e (
. . . . Biphyllus lunatus .................................        1 hit  [beetles]         Biphyllus lunatus mRNA for ribosomal protein L12e (rpL1
. . . . Drosophila pseudoobscura pseudoobscura ............        1 hit  [flies]           Drosophila pseudoobscura pseudoobscura GA16582 (Dpse\GA
. . . . Drosophila persimilis .............................        1 hit  [flies]           Drosophila persimilis GL11659 (Dper\GL11659), mRNA
. . . . Sarcophaga crassipalpis ...........................        1 hit  [flies]           TSA: Sarcophaga crassipalpis HAHN.FLY.99.C1 mRNA sequen
. . . . Argopecten irradians ..............................        1 hit  [bivalves]        Argopecten irradians isolate iolsaicl413ct453cn476 ribo
. . . . Schistosoma japonicum .............................        8 hits [flatworms]       Schistosoma japonicum isolate Anhui full length mRNA cl
. . . . Drosophila mojavensis .............................        1 hit  [flies]           Drosophila mojavensis GI18857 (Dmoj\GI18857), mRNA
. . . . Anopheles funestus ................................        1 hit  [flies]           Anopheles funestus clone AF-44 ribosomal protein L12 mR
. . . . Rattus norvegicus (brown rat) .....................        4 hits [rodents]         PREDICTED: Rattus norvegicus similar to 60S ribosomal p
. . . . Drosophila virilis ................................        1 hit  [flies]           Drosophila virilis GJ20445 (Dvir\GJ20445), mRNA
. . . . Flustra foliacea ..................................        1 hit  [bryozoans]       Flustra foliacea putative 60S ribosomal protein RPL12 m
. . . . Rattus rattus (roof rat) ..........................        1 hit  [rodents]         Rat mRNA for ribosomal protein L12
. . . . Mustela putorius furo (ferret) ....................        1 hit  [carnivores]      TSA: Mustela putorius furo Ferret c1444, complete seque
. . . . Mus musculus (mouse) ..............................       14 hits [rodents]         Mus musculus predicted pseudogene 10275 (Gm10275) on ch
. . . . Pongo abelii (Orang-utan) .........................        4 hits [primates]        PREDICTED: Pongo abelii 60S ribosomal protein L12-like,
. . . . Callithrix jacchus (common marmoset) ..............        1 hit  [primates]        PREDICTED: Callithrix jacchus 60S ribosomal protein L12
. . . . Pan troglodytes ...................................        3 hits [primates]        PREDICTED: Pan troglodytes similar to 60S ribosomal pro
. . . . Homo sapiens (man) ................................        5 hits [primates]        Homo sapiens cDNA clone IMAGE:6597277, **** WARNING: ch
. . . Trichoplax adhaerens --------------------------------        1 hit  [placozoans]      Trichoplax adhaerens expressed hypothetical protein, mR
. . . Suberites domuncula .................................        1 hit  [sponges]         Suberites domuncula L12 mRNA, complete cds
. . Coprinopsis cinerea okayama7#130 ----------------------        1 hit  [basidiomycetes]  Coprinopsis cinerea okayama7#130 SeqLit ribosomal prote
. . Schizophyllum commune H4-8 ............................        1 hit  [basidiomycetes]  Schizophyllum commune H4-8 hypothetical protein, mRNA
. . Laccaria bicolor S238N-H82 ............................        1 hit  [basidiomycetes]  Laccaria bicolor S238N-H82 hypothetical protein partial
. synthetic construct -------------------------------------        2 hits [other sequences] Synthetic construct Homo sapiens clone FLH029540.01L ri
```

---

### Organism Report

```
 Acropora millepora [stony corals] taxid 45264
gb|EZ024706.1| TSA: Acropora millepora SeqIndex4149, mRNA ...     ____   0.0

 Anthopleura elegantissima [sea anemones] taxid 6110
gb|DQ314616.1| Anthopleura elegantissima ribosomal protein...     ____   0.0

 Microcosmus squamiger [tunicates] taxid 439822
emb|FN984766.1| Microcosmus squamiger partial mRNA for rib...     ____   0.0

 Branchiostoma floridae [lancelets] taxid 7739
ref|XM_002591796.1| Branchiostoma floridae hypothetical pr...     ____   0.0

 Pediculus humanus corporis (human body lice) [lice] taxid 121224
ref|XM_002425415.1| Pediculus humanus corporis 60S ribosom...     ____   0.0

 Patiria miniata [starfish] taxid 46514
gb|HP092325.1| TSA: Patiria miniata isotig10042.Pminbgast ...     ____   0.0

 Nematostella vectensis [sea anemones] taxid 45351
ref|XM_001641997.1| Nematostella vectensis predicted prote...     ____   0.0

 Phragmatopoma lapidosa [segmented worms] taxid 341668
gb|GQ455407.1| Phragmatopoma lapidosa ribosomal-like prote...     ____   0.0

 Maconellicoccus hirsutus (pink hibiscus mealybug, ...) [scale insects] taxid 177089
```

gb|EF070571.1| Maconellicoccus hirsutus clone WHMH4004 put... 0.0

**Nasonia vitripennis** [wasps &c.] taxid 7425
ref|XM_001605592.1| PREDICTED: Nasonia vitripennis similar... 0.0

**Solea senegalensis** [bony fishes] taxid 28829
dbj|AB374947.1| Solea senegalensis RPL12 mRNA for ribosoma... 0.0

**Trichoplax adhaerens** [placozoans] taxid 10228
ref|XM_002108586.1| Trichoplax adhaerens expressed hypothe... 0.0

**Ornithodoros parkeri** [mites & ticks] taxid 140564
gb|EF633964.1| Ornithodoros parkeri clone OP-85 40S riboso... 0.0

**Ixodes scapularis** (shoulder tick, ...) [mites & ticks] taxid 6945
ref|XM_002433985.1| Ixodes scapularis ribosomal protein L1... 0.0
gb|DQ066203.1| Ixodes scapularis isolate ISUFL56 ribosomal... 0.0

**Dermacentor variabilis** [mites & ticks] taxid 34621
gb|EU551645.1| Dermacentor variabilis isolate DvM 40 ribos... 0.0

**Coprinopsis cinerea okayama7#130** [basidiomycetes] taxid 240176
ref|XM_001830397.2| Coprinopsis cinerea okayama7#130 SeqLi... 0.0

**Acyrthosiphon pisum** [aphids] taxid 7029
gb|HP395106.1| TSA: Acyrthosiphon pisum strain LSR1 Contig... 0.0
gb|HP301142.1| TSA: Acyrthosiphon pisum strain LSR1 Contig... 0.0
dbj|AK340119.1| Acyrthosiphon pisum ACYPI000059 mRNA, clon... 0.0
ref|NM_001126171.2| Acyrthosiphon pisum ribosomal protein ... 0.0
gb|DQ416025.1| Acyrthosiphon pisum putative ribosomal prot... 0.0

**Hypophthalmichthys nobilis** (noble carp, ...) [bony fishes] taxid 7965
gb|HM146124.1| Hypophthalmichthys nobilis ribosomal protei... 0.0

**Salmo salar** [bony fishes] taxid 8030
gb|BT048569.2| Salmo salar clone ssal-plnb-020-145 60S rib... 0.0
gb|BT056663.1| Salmo salar clone ssal-rgb2-526-129 60S rib... 0.0
gb|BT043744.1| Salmo salar clone HM6_0646 ribosomal protei... 0.0

**Rhipicephalus sanguineus** [mites & ticks] taxid 34632
gb|EZ406119.1| TSA: Rhipicephalus sanguineus RS-401 mRNA s... 0.0

**Tribolium castaneum** (rust-red flour beetle) [beetles] taxid 7070
ref|XM_970437.2| PREDICTED: Tribolium castaneum similar to... 0.0

**Eurythoe complanata** [segmented worms] taxid 167815
gb|EU125012.1| Eurythoe complanata ribosomal protein rpl12... 0.0

**Schizophyllum commune H4-8** [basidiomycetes] taxid 578458
ref|XM_003029870.1| Schizophyllum commune H4-8 hypothetica... 0.0

**Phoronis muelleri** [phoronid worms] taxid 478209
gb|EU558362.1| Phoronis muelleri putative 60S ribosomal pr... 0.0

**Anopheles gambiae str. PEST** [flies] taxid 180454
ref|XM_319222.4| Anopheles gambiae str. PEST AGAP010065-PA... 0.0

**Anopheles gambiae** [flies] taxid 7165
emb|BX063995.1| Single read from an extremity of a full-le... 0.0
emb|BX009066.1| Single read from an extremity of a full-le... 0.0

**Suberites domuncula** [sponges] taxid 55567
gb|AY857425.1| Suberites domuncula L12 mRNA, complete cds 0.0

**Timarcha balearica** [beetles] taxid 79517
emb|AM049032.1| Timarcha balearica mRNA for ribosomal prot... 0.0

**Rhagoletis pomonella** [flies] taxid 28610
gb|EZ125760.1| TSA: Rhagoletis pomonella contig09541, mRNA... 0.0

**Saccoglossus kowalevskii** [hemichordates] taxid 10224
ref|XM_002741431.1| PREDICTED: Saccoglossus kowalevskii ri... 0.0

**Ciona intestinalis** [tunicates] taxid 7719
ref|XM_002131525.1| PREDICTED: Ciona intestinalis similar ... 0.0
dbj|AK113209.1| Ciona intestinalis cDNA, clone:ciad015o19,... 0.0

**Macaca mulatta** (rhesus macaque, ...) [primates] taxid 9544
ref|XR_014217.2| PREDICTED: Macaca mulatta 60S ribosomal p... 0.0
ref|NM_001193560.1| Macaca mulatta ribosomal protein L12 (... 0.0
ref|XM_001114984.2| PREDICTED: Macaca mulatta 60S ribosoma... 0.0
ref|XM_002801377.1| PREDICTED: Macaca mulatta 60S ribosoma... 0.0
ref|XM_002801376.1| PREDICTED: Macaca mulatta 60S ribosoma... 0.0

**Laccaria bicolor S238N-H82** [basidiomycetes] taxid 486041
ref|XM_001880189.1| Laccaria bicolor S238N-H82 hypothetica... 0.0

**Scarabaeus laticollis** [beetles] taxid 292456
emb|AM049030.1| Scarabaeus laticollis mRNA for ribosomal p... 0.0

**Biphyllus lunatus** [beetles] taxid 197003
emb|AM049028.1| Biphyllus lunatus mRNA for ribosomal prote... 0.0

**Drosophila pseudoobscura pseudoobscura** [flies] taxid 46245
ref|XM_001360854.2| Drosophila pseudoobscura pseudoobscura... 0.0

**Drosophila persimilis** [flies] taxid 7234
ref|XM_002016543.1| Drosophila persimilis GL11659 (Dper\GL... 0.0

**Sarcophaga crassipalpis** [flies] taxid 59312
gb|EZ597013.1| TSA: Sarcophaga crassipalpis HAHN.FLY.99.C1... 0.0

**Argopecten irradians** [bivalves] taxid 31199
gb|AF526229.1| Argopecten irradians isolate iolsaicl413ct4... 0.0

**Schistosoma japonicum** [flatworms] taxid 6182
emb|FN315220.1| Schistosoma japonicum isolate Anhui full l... 0.0
emb|FN315219.1| Schistosoma japonicum isolate Anhui full l... 0.0

```
emb|FN320441.1| Schistosoma japonicum isolate Anhui full l...      0.0
emb|FN320440.1| Schistosoma japonicum isolate Anhui full l...      0.0
emb|FN320439.1| Schistosoma japonicum isolate Anhui full l...      0.0
emb|FN320438.1| Schistosoma japonicum isolate Anhui full l...      0.0
emb|FN320436.1| Schistosoma japonicum isolate Anhui full l...      0.0
gb|AY814172.1| Schistosoma japonicum SJCHGC06254 protein m...      0.0

 Montastraea franksi [stony corals] taxid 48499
gb|FJ790233.1| Montastraea franksi ribosomal protein L12 (...      0.0

 Drosophila mojavensis [flies] taxid 7230
ref|XM_002005813.1| Drosophila mojavensis GI18857 (Dmoj\GI...      0.0

 Anopheles funestus [flies] taxid 62324
gb|DQ910352.1| Anopheles funestus clone AF-44 ribosomal pr...      0.0

 Rattus norvegicus (brown rat, ...) [rodents] taxid 10116
ref|XM_001056336.2| PREDICTED: Rattus norvegicus similar t...      0.0
ref|XM_002727523.1| PREDICTED: Rattus norvegicus similar t...      0.0
gb|BC166903.1| Rattus norvegicus similar to 60S ribosomal ...      0.0
ref|NM_001109198.1| Rattus norvegicus similar to 60S ribos...      0.0

 Drosophila virilis [flies] taxid 7244
ref|XM_002049947.1| Drosophila virilis GJ20445 (Dvir\GJ204...      0.0

 Flustra foliacea [bryozoans] taxid 478208
gb|EU139209.1| Flustra foliacea putative 60S ribosomal pro...      0.0

 Rattus rattus (roof rat, ...) [rodents] taxid 10117
emb|X53504.1| Rat mRNA for ribosomal protein L12                   0.0

 Mustela putorius furo (ferret, ...) [carnivores] taxid 9669
gb|EZ457883.1| TSA: Mustela putorius furo Ferret_c1444, co...      0.0

 Mus musculus (mouse) [rodents] taxid 10090
ref|NG_004759.6| Mus musculus predicted pseudogene 10275 (...      0.0
ref|NG_007061.2| Mus musculus predicted gene 9396 (Gm9396)...      0.0
ref|NM_009076.3| Mus musculus ribosomal protein L12 (Rpl12...      0.0
gb|AC121149.12| Mus musculus chromosome 3, clone RP24-383A...      0.0
gb|BC081469.1| Mus musculus ribosomal protein L12, mRNA (c...      0.0
gb|AC164078.4| Mus musculus chromosome 3, clone RP23-123C1...      0.0
gb|AC166262.3| Mus musculus  BAC RP23-256D10 (Roswell Park...      0.0
dbj|AK150318.1| Mus musculus bone marrow macrophage cDNA, ...      0.0
dbj|AK168121.1| Mus musculus CRL-1722 L5178Y-R cDNA, RIKEN...      0.0
dbj|AK144905.1| Mus musculus lung RCB-0558 LLC cDNA, RIKEN...      0.0
dbj|AK151592.1| Mus musculus bone marrow macrophage cDNA, ...      0.0
dbj|AK151532.1| Mus musculus bone marrow macrophage cDNA, ...      0.0
gb|BC090393.1| Mus musculus ribosomal protein L12, mRNA (c...      0.0
gb|AC118620.10| Mus musculus chromosome 3, clone RP24-571H...      0.0

 Pongo abelii (Orang-utan, ...) [primates] taxid 9601
ref|XM_002821249.1| PREDICTED: Pongo abelii 60S ribosomal ...      0.0
ref|XM_002821248.1| PREDICTED: Pongo abelii 60S ribosomal ...      0.0
ref|XM_002821247.1| PREDICTED: Pongo abelii 60S ribosomal ...      0.0
ref|XM_002820230.1| PREDICTED: Pongo abelii 60S ribosomal ...      0.0

 Callithrix jacchus (common marmoset, ...) [primates] taxid 9483
ref|XM_002754372.1| PREDICTED: Callithrix jacchus 60S ribo...      0.0

 Pan troglodytes [primates] taxid 9598
ref|XM_528436.2| PREDICTED: Pan troglodytes similar to 60S...      0.0
ref|XM_001137362.1| PREDICTED: Pan troglodytes similar to ...      0.0
ref|XM_516562.2| PREDICTED: Pan troglodytes similar to rib...      0.0

 Homo sapiens (man) [primates] taxid 9606
gb|BC085006.1| Homo sapiens cDNA clone IMAGE:6597277, ****...      0.0
gb|BC071921.1| Homo sapiens cDNA clone IMAGE:6423605, ****...      0.0
gb|BC001802.1| Homo sapiens cDNA clone IMAGE:3355546, ****...      0.0
emb|CR625201.1| full-length cDNA clone CS0DC011YI15 of Neu...      0.0
emb|CR622190.1| full-length cDNA clone CS0DL009YN18 of B c...      0.0

 synthetic construct [other sequences] taxid 32630
gb|AY891709.1| Synthetic construct Homo sapiens clone FLH0...      0.0
gb|AY889175.1| Synthetic construct Homo sapiens clone FLH0...      0.0
```

---

**Taxonomy Report**

```
root .............................................................. 100 hits  54 orgs
. Fungi/Metazoa group ............................................  98 hits  53 orgs [cellular organisms; Eukaryota]
. . Metazoa ......................................................  95 hits  50 orgs
. . . Eumetazoa ..................................................  93 hits  48 orgs
. . . . Hexacorallia .............................................   4 hits   4 orgs [Cnidaria; Anthozoa]
. . . . . Scleractinia ...........................................   2 hits   2 orgs
. . . . . . Acropora millepora ...................................   1 hits   1 orgs [Astrocoeniina; Acroporidae; Acropora]
. . . . . . Montastraea franksi ..................................   1 hits   1 orgs [Faviina; Faviidae; Montastraea]
. . . . . Actiniaria .............................................   2 hits   2 orgs
. . . . . . Anthopleura elegantissima ............................   1 hits   1 orgs [Nynantheae; Actiniidae; Anthopleura]
. . . . . . Nematostella vectensis ...............................   1 hits   1 orgs [Edwardsiidae; Nematostella]
. . . . Bilateria ................................................  89 hits  44 orgs
. . . . . Coelomata ..............................................  81 hits  43 orgs
. . . . . . Deuterostomia ........................................  49 hits  17 orgs
. . . . . . . Chordata ...........................................  47 hits  15 orgs
. . . . . . . . Ascidiacea .......................................   3 hits   2 orgs [Tunicata]
. . . . . . . . . Microcosmus squamiger ..........................   1 hits   1 orgs [Stolidobranchia; Pyuridae; Microcosmus]
. . . . . . . . . Ciona intestinalis .............................   2 hits   1 orgs [Enterogona; Phlebobranchia; Cionidae; Ciona]
. . . . . . . . Branchiostoma floridae ...........................   1 hits   1 orgs [Cephalochordata; Branchiostomidae; Branchiostoma]
. . . . . . . . Euteleostomi .....................................  43 hits  12 orgs [Craniata; Vertebrata; Gnathostomata; Teleostomi]
. . . . . . . . . Clupeocephala ..................................   5 hits   3 orgs [Actinopterygii; Actinopteri; Neopterygii; Teleostei; E
. . . . . . . . . . Euteleostei ..................................   4 hits   2 orgs
. . . . . . . . . . . Solea senegalensis .........................   1 hits   1 orgs [Neognathi; Neoteleostei; Eurypterygii; Ctenosquamata; 
. . . . . . . . . . . Salmo salar ................................   3 hits   1 orgs [Protacanthopterygii; Salmoniformes; Salmonoidei; Salmo
. . . . . . . . . . Hypophthalmichthys nobilis ...................   1 hits   1 orgs [Otocephala; Ostariophysi; Otophysi; Cypriniphysi; Cypr
. . . . . . . . . Eutheria .......................................  38 hits   9 orgs [Sarcopterygii; Tetrapoda; Amniota; Mammalia; Theria]
. . . . . . . . . . Euarchontoglires .............................  37 hits   8 orgs
. . . . . . . . . . . Simiiformes ................................  18 hits   5 orgs [Primates; Haplorrhini]
. . . . . . . . . . . . Catarrhini ...............................  17 hits   4 orgs
. . . . . . . . . . . . . Macaca mulatta .........................   5 hits   1 orgs [Cercopithecoidea; Cercopithecidae; Cercopithecinae; Ma
. . . . . . . . . . . . . Hominidae ..............................  12 hits   3 orgs [Hominoidea]
. . . . . . . . . . . . . . Pongo abelii .........................   4 hits   1 orgs [Ponginae; Pongo]
```

```
. . . . . . . . . . . . . . Homininae ...............................    8 hits    2 orgs
. . . . . . . . . . . . . . . Pan troglodytes .......................    3 hits    1 orgs [Pan]
. . . . . . . . . . . . . . . Homo sapiens ..........................    5 hits    1 orgs [Homo]
. . . . . . . . . . . . Callithrix jacchus ..........................    1 hits    1 orgs [Platyrrhini; Cebidae; Callitrichinae; Callithrix]
. . . . . . . . . . . Murinae .......................................   19 hits    3 orgs [Glires; Rodentia; Sciurognathi; Muroidea; Muridae]
. . . . . . . . . . . . Rattus ......................................    5 hits    2 orgs
. . . . . . . . . . . . . Rattus norvegicus .........................    4 hits    1 orgs
. . . . . . . . . . . . . Rattus rattus .............................    1 hits    1 orgs
. . . . . . . . . . . . Mus musculus ................................   14 hits    1 orgs [Mus; Mus]
. . . . . . . . . . Mustela putorius furo ...........................    1 hits    1 orgs [Laurasiatheria; Carnivora; Caniformia; Mustelidae; Mus
. . . . . . . Patiria miniata .......................................    1 hits    1 orgs [Echinodermata; Eleutherozoa; Asterozoa; Asteroidea; Va
. . . . . . . Saccoglossus kowalevskii ..............................    1 hits    1 orgs [Hemichordata; Enteropneusta; Harrimaniidae; Saccogloss
. . . . . . Protostomia .............................................   32 hits   26 orgs
. . . . . . . Arthropoda ............................................   27 hits   21 orgs [Panarthropoda]
. . . . . . . . Neoptera ............................................   22 hits   17 orgs [Mandibulata; Pancrustacea; Hexapoda; Insecta; Dicondyl
. . . . . . . . . Paraneoptera ......................................    7 hits    3 orgs
. . . . . . . . . . Pediculus humanus corporis ......................    1 hits    1 orgs [Phthiraptera; Anoplura; Pediculidae; Pediculus; Pedicu
. . . . . . . . . . Aphidiformes ....................................    6 hits    2 orgs [Hemiptera; Sternorrhyncha]
. . . . . . . . . . . Maconellicoccus hirsutus ......................    1 hits    1 orgs [Coccoidea; Pseudococcidae; Maconellicoccus]
. . . . . . . . . . . Acyrthosiphon pisum ...........................    5 hits    1 orgs [Aphidomorpha; Aphidoidea; Aphididae; Aphidinae; Macros
. . . . . . . . . Endopterygota .....................................   15 hits   14 orgs
. . . . . . . . . . Nasonia vitripennis .............................    1 hits    1 orgs [Hymenoptera; Apocrita; Chaldicoidea group; Chalcidoide
. . . . . . . . . . Polyphaga .......................................    4 hits    4 orgs [Coleoptera]
. . . . . . . . . . . Cucujiformia ..................................    3 hits    3 orgs
. . . . . . . . . . . . Tribolium castaneum .........................    1 hits    1 orgs [Tenebrionoidea; Tenebrionidae; Tribolium]
. . . . . . . . . . . . Timarcha balearica ..........................    1 hits    1 orgs [Chrysomeloidea; Chrysomelidae; Chrysomelinae; Timarchi
. . . . . . . . . . . . Biphyllus lunatus ...........................    1 hits    1 orgs [Cucujoidea; Biphyllidae; Biphyllus]
. . . . . . . . . . . Scarabaeus laticollis .........................    1 hits    1 orgs [Scarabaeiformia; Scarabaeoidea; Scarabaeidae; Scarabae
. . . . . . . . . . Diptera .........................................   10 hits    9 orgs
. . . . . . . . . . . Cellia ........................................    4 hits    3 orgs [Nematocera; Culicimorpha; Culicoidea; Culicidae; Anoph
. . . . . . . . . . . . gambiae species complex .....................    3 hits    2 orgs [Pyretophorus]
. . . . . . . . . . . . . Anopheles gambiae .........................    3 hits    2 orgs
. . . . . . . . . . . . . . Anopheles gambiae str. PEST .............    1 hits    1 orgs
. . . . . . . . . . . . Anopheles funestus ..........................    1 hits    1 orgs [Myzomyia; funestus group; funestus subgroup]
. . . . . . . . . . . Schizophora ...................................    6 hits    6 orgs [Brachycera; Muscomorpha; Eremoneura; Cyclorrhapha]
. . . . . . . . . . . . Acalyptratae ................................    5 hits    5 orgs
. . . . . . . . . . . . . Rhagoletis pomonella ......................    1 hits    1 orgs [Tephritoidea; Tephritidae; Trypetinae; Carpomyini; Car
. . . . . . . . . . . . . Drosophila ................................    4 hits    4 orgs [Ephydroidea; Drosophilidae; Drosophilinae; Drosophilin
. . . . . . . . . . . . . . pseudoobscura subgroup ..................    2 hits    2 orgs [Sophophora; obscura group]
. . . . . . . . . . . . . . . Drosophila pseudoobscura pseudoobscura .    1 hits    1 orgs [Drosophila pseudoobscura]
. . . . . . . . . . . . . . . Drosophila persimilis .................    1 hits    1 orgs
. . . . . . . . . . . . . . Drosophila ..............................    2 hits    2 orgs
. . . . . . . . . . . . . . . Drosophila mojavensis .................    1 hits    1 orgs [repleta group; mulleri subgroup; mojavensis species co
. . . . . . . . . . . . . . . Drosophila virilis ....................    1 hits    1 orgs [virilis group]
. . . . . . . . . . . . Sarcophaga crassipalpis .....................    1 hits    1 orgs [Calyptratae; Oestroidea; Sarcophagidae; Sarcophaginae;
. . . . . . . . Ixodoidea ...........................................    5 hits    4 orgs [Chelicerata; Arachnida; Acari; Parasitiformes; Ixodida
. . . . . . . . . Ornithodoros parkeri ..............................    1 hits    1 orgs [Argasidae; Ornithodorinae; Ornithodoros]
. . . . . . . . . Ixodidae ..........................................    4 hits    3 orgs
. . . . . . . . . . Ixodes scapularis ...............................    2 hits    1 orgs [Ixodinae; Ixodes]
. . . . . . . . . . Rhipicephalinae .................................    2 hits    2 orgs
. . . . . . . . . . . Dermacentor variabilis ........................    1 hits    1 orgs [Dermacentor]
. . . . . . . . . . . Rhipicephalus sanguineus ......................    1 hits    1 orgs [Rhipicephalus; Rhipicephalus; Rhipicephalus sanguineus
. . . . . . . Palpata ...............................................    2 hits    2 orgs [Annelida/Echiura/Pogonophora group; Annelida; Polychae
. . . . . . . . Phragmatopoma lapidosa ..............................    1 hits    1 orgs [Canalipalpata; Sabellida; Sabellariidae; Phragmatopoma
. . . . . . . . Eurythoe complanata .................................    1 hits    1 orgs [Aciculata; Eunicida; Amphinomidae; Eurythoe]
. . . . . . . Phoronis muelleri .....................................    1 hits    1 orgs [Brachiopoda; Phoroniformea; Phoronis]
. . . . . . . Argopecten irradians ..................................    1 hits    1 orgs [Mollusca; Bivalvia; Pteriomorphia; Pectinoida; Pectino
. . . . . . . Flustra foliacea ......................................    1 hits    1 orgs [Bryozoa; Gymnolaemata; Cheilostomatida; Anasca; Flustr
. . . . . Schistosoma japonicum .....................................    8 hits    1 orgs [Acoelomata; Platyhelminthes; Trematoda; Digenea; Strig
. . . Trichoplax adhaerens ..........................................    1 hits    1 orgs [Placozoa; Trichoplax]
. . . Suberites domuncula ...........................................    1 hits    1 orgs [Porifera; Demospongiae; Tetractinomorpha; Hadromerida;
. . Agaricales ......................................................    3 hits    3 orgs [Fungi; Dikarya; Basidiomycota; Agaricomycotina; Agaric
. . . Coprinopsis cinerea okayama7#130 ..............................    1 hits    1 orgs [Psathyrellaceae; Coprinopsis; Coprinopsis cinerea]
. . . Schizophyllum commune H4-8 ....................................    1 hits    1 orgs [Schizophyllaceae; Schizophyllum; Schizophyllum commune
. . . Laccaria bicolor S238N-H82 ....................................    1 hits    1 orgs [Tricholomataceae; Laccaria; Laccaria bicolor]
. synthetic construct ...............................................    2 hits    1 orgs [other sequences; artificial sequences]
```

**BLAST**

## Basic Local Alignment Search Tool

Jump to Page Content

**Job Title: (2) - EZ040625**

---

- 

### Tax BLAST Report

**Index**


- Lineage Report
- Organism Report
- Taxonomy Report
- Help


**Lineage Report**

```
root
. Metazoa      [animals]
. . Eumetazoa    [animals]
. . . Cnidaria     [cnidarians]
. . . . Hexacorallia [anthozoans]
. . . . . Acropora millepora ---------------------------       1 hit  [stony corals]        TSA: Acropora millepora SeqIndex8015, mRNA sequence
. . . . . Nematostella vectensis .......................       1 hit  [sea anemones]        Nematostella vectensis predicted protein (NEMVEDRAFT
. . . . Hydra magnipapillata ---------------------------       1 hit  [hydrozoans]          PREDICTED: Hydra magnipapillata similar to predicted
. . . Eurythoe complanata ------------------------------       1 hit  [segmented worms]     Eurythoe complanata ribosomal protein rpl13a mRNA, co
. . . Taeniopygia guttata (zebra finch) ................       7 hits [birds]               Taeniopygia guttata clone 0058P0011D10 putative ribos
. . . Schistosoma mansoni ..............................       1 hit  [flatworms]           Schistosoma mansoni 60S ribosomal protein L13a, putat
. . . Strongylocentrotus purpuratus (purple urchin) ....       2 hits [sea urchins]         PREDICTED: Strongylocentrotus purpuratus similar to R
. . . Tribolium castaneum (rust-red flour beetle) ......       1 hit  [beetles]             PREDICTED: Tribolium castaneum similar to ribosomal p
. . . Ciona intestinalis ...............................       1 hit  [tunicates]           Ciona intestinalis cDNA, clone:cicl013b04, full inser
. . . Schistosoma japonicum ............................       1 hit  [flatworms]           Schistosoma japonicum SJCHGC04249 protein mRNA, compl
. . . Nasonia vitripennis ..............................       1 hit  [wasps &c.]           PREDICTED: Nasonia vitripennis similar to ribosomal p
. . . Oncorhynchus masou formosanus (Taiwan salmon) ....       1 hit  [bony fishes]         Oncorhynchus masou formosanus ribosomal protein L13A
. . . Gallus gallus (bantam) ...........................       1 hit  [birds]               Gallus gallus finished cDNA, clone ChEST790c21
. . . Oncorhynchus mykiss ..............................       1 hit  [bony fishes]         TSA: Oncorhynchus mykiss 142569.Onmycontig mRNA seque
. . . Salmo salar ......................................       9 hits [bony fishes]         Salmo salar clone ssal-rgh-518-022 60S ribosomal prot
. . . Anoplopoma fimbria ...............................       1 hit  [bony fishes]         Anoplopoma fimbria clone afim-evh-508-275 60S ribosom
. . . Xenopus laevis (common platanna) .................       2 hits [frogs & toads]       Xenopus laevis ribosomal protein L13a (rpl13a), mRNA
. . . Solea senegalensis ...............................       2 hits [bony fishes]         Solea senegalensis RPL13a mRNA for ribosomal protein
. . . Homo sapiens (man) ...............................      15 hits [primates]            Homo sapiens cDNA clone IMAGE:6423605, **** WARNING:
. . . Branchiostoma floridae ...........................       1 hit  [lancelets]           Branchiostoma floridae hypothetical protein, mRNA
. . . Ornithoctonus huwena (Chinese golden earth t...) .       1 hit  [spiders]             Ornithoctonus huwena clone HWE499 60S ribosomal prote
. . . Gasterosteus aculeatus (three spined stickleback) .      1 hit  [bony fishes]         Gasterosteus aculeatus clone CEC39-G07 mRNA sequence
. . . Pan troglodytes ..................................      13 hits [primates]            PREDICTED: Pan troglodytes similar to ribosomal prote
. . . Xenopus (Silurana) tropicalis ....................       6 hits [frogs & toads]       Xenopus tropicalis hypothetical protein LOC549240, mR
. . . Bos taurus (cow) .................................       3 hits [even-toed ungulates] Bos taurus ribosomal protein L13a (RPL13A), mRNA >gi|
. . . Ictalurus furcatus ...............................       1 hit  [bony fishes]         TSA: Ictalurus furcatus Contig11132.Icfu mRNA sequenc
. . . Monodelphis domestica ............................       1 hit  [marsupials]          PREDICTED: Monodelphis domestica hypothetical protein
. . . Platichthys flesus ...............................       1 hit  [bony fishes]         Platichthys flesus mRNA for 60S ribosomal protein L13
. . . Sus scrofa (wild boar) ...........................       2 hits [even-toed ungulates] PREDICTED: Sus scrofa bcl-2-like protein 12-like (LOC
. . . Callithrix jacchus (common marmoset) .............       2 hits [primates]            PREDICTED: Callithrix jacchus 60S ribosomal protein L
. . . Lycosa singoriensis ..............................       1 hit  [spiders]             Lycosa singoriensis 60S ribosomal protein L13A mRNA,
. . . Macaca fascicularis (cynomolgus monkey) ..........       2 hits [primates]            Macaca fascicularis brain cDNA clone: QbsB-11018, sim
. . . Macaca mulatta (rhesus macaque) ..................       1 hit  [primates]            Macaca mulatta ribosomal protein L13a (RPL13A), mRNA
. . . Ailuropoda melanoleuca ...........................       1 hit  [carnivores]          PREDICTED: Ailuropoda melanoleuca 60S ribosomal prote
. . . Pongo abelii (Orang-utan) ........................       9 hits [primates]            PREDICTED: Pongo abelii 60S ribosomal protein L13a-li
. . . Equus caballus (equine) ..........................       1 hit  [odd-toed ungulates]  PREDICTED: Equus caballus similar to ribosomal protei
. . Trichoplax adhaerens -------------------------------       1 hit  [placozoans]          Trichoplax adhaerens expressed hypothetical protein,
. . Suberites domuncula ................................       1 hit  [sponges]             Suberites domuncula L13a mRNA, complete cds
. synthetic construct ----------------------------------       4 hits [other sequences]     Synthetic construct Homo sapiens clone HAIB:100066484
```

---

**Organism Report**

```
 Acropora millepora [stony corals] taxid 45264
gb|EZ040625.1| TSA: Acropora millepora SeqIndex8015, mRNA ...        0.0

 Nematostella vectensis [sea anemones] taxid 45351
ref|XM_001630762.1| Nematostella vectensis predicted prote...        0.0

 Eurythoe complanata [segmented worms] taxid 167815
gb|EU125014.1| Eurythoe complanata ribosomal protein rpl13...        0.0

 Taeniopygia guttata (zebra finch) [birds] taxid 59729
gb|DQ216005.1| Taeniopygia guttata clone 0058P0011D10 puta...        0.0
gb|DQ216004.1| Taeniopygia guttata clone 0058P0006F05 puta...        0.0
gb|DQ216003.1| Taeniopygia guttata clone 0058P0045E08 ribo...        0.0
gb|DQ216000.1| Taeniopygia guttata clone 0058P0016A02 puta...        0.0
gb|DQ216006.1| Taeniopygia guttata clone 0061P0024G09 puta...        0.0
gb|DQ216002.1| Taeniopygia guttata clone 0058P0010A09 ribo...        0.0
gb|DQ216001.1| Taeniopygia guttata clone 0058P0049C02 puta...        0.0

 Trichoplax adhaerens [placozoans] taxid 10228
ref|XM_002112857.1| Trichoplax adhaerens expressed hypothe...        0.0

 Schistosoma mansoni [flatworms] taxid 6183
ref|XM_002580005.1| Schistosoma mansoni 60S ribosomal prot...        0.0

 Suberites domuncula [sponges] taxid 55567
gb|AY857427.1| Suberites domuncula L13a mRNA, complete cds           0.0

 Strongylocentrotus purpuratus (purple urchin, ...) [sea urchins] taxid 7668
ref|XM_001177209.1| PREDICTED: Strongylocentrotus purpurat...        0.0
ref|XM_779422.2| PREDICTED: Strongylocentrotus purpuratus ...        0.0

 Tribolium castaneum (rust-red flour beetle) [beetles] taxid 7070
ref|XM_969211.2| PREDICTED: Tribolium castaneum similar to...        0.0

 Ciona intestinalis [tunicates] taxid 7719
dbj|AK174254.1| Ciona intestinalis cDNA, clone:cicl013b04,...        0.0

 Schistosoma japonicum [flatworms] taxid 6182
gb|AY815942.1| Schistosoma japonicum SJCHGC04249 protein m...        0.0
```

**Nasonia vitripennis** [wasps &c.] taxid 7425
ref|XM_001607808.1| PREDICTED: Nasonia vitripennis similar... ____ 0.0

**Oncorhynchus masou formosanus** (Taiwan salmon) [bony fishes] taxid 173242
gb|EU325865.1| Oncorhynchus masou formosanus ribosomal pro... ____ 0.0

**Hydra magnipapillata** [hydrozoans] taxid 6085
ref|XM_002162001.1| PREDICTED: Hydra magnipapillata simila... ____ 0.0

**Gallus gallus** (bantam, ...) [birds] taxid 9031
emb|BX931917.1| Gallus gallus finished cDNA, clone ChEST79... ____ 0.0

**Oncorhynchus mykiss** [bony fishes] taxid 8022
gb|EZ905903.1| TSA: Oncorhynchus mykiss 142569.Onmycontig ... ____ 0.0

**Salmo salar** [bony fishes] taxid 8030
gb|BT060382.1| Salmo salar clone ssal-rgh-518-022 60S ribo... ____ 0.0
gb|BT060180.1| Salmo salar clone ssal-rgh-507-303 60S ribo... ____ 0.0
gb|BT059935.1| Salmo salar clone ssal-rgg-510-349 60S ribo... ____ 0.0
gb|BT058667.1| Salmo salar clone Contig3117 60S ribosomal ... ____ 0.0
gb|BT058473.1| Salmo salar clone Contig1152 60S ribosomal ... ____ 0.0
gb|BT058235.1| Salmo salar clone Contig2288 60S ribosomal ... ____ 0.0
gb|BT048067.1| Salmo salar clone ssal-eve-575-212 60S ribo... ____ 0.0
gb|BT044039.1| Salmo salar clone HM6_0617 ribosomal protei... ____ 0.0
gb|BT044023.1| Salmo salar clone HM6_0789 ribosomal protei... ____ 0.0

**Anoplopoma fimbria** [bony fishes] taxid 229290
gb|BT082277.1| Anoplopoma fimbria clone afim-evh-508-275 6... ____ 0.0

**Xenopus laevis** (common platanna, ...) [frogs & toads] taxid 8355
ref|NM_001086661.1| Xenopus laevis ribosomal protein L13a ... ____ 0.0
gb|BC043976.1| Xenopus laevis ribosomal protein L13a, mRNA... ____ 0.0

**Solea senegalensis** [bony fishes] taxid 28829
dbj|AB360597.1| Solea senegalensis RPL13a mRNA for ribosom... ____ 0.0
dbj|AB374949.1| Solea senegalensis RPL13a-like1 mRNA for r... ____ 0.0

**Homo sapiens** (man) [primates] taxid 9606
gb|BC071921.1| Homo sapiens cDNA clone IMAGE:6423605, ****... ____ 0.0
gb|BC062537.1| Homo sapiens ribosomal protein L13a, mRNA (... ____ 0.0
dbj|AK291120.1| Homo sapiens cDNA FLJ75551 complete cds, h... ____ 0.0
emb|CR625138.1| full-length cDNA clone CS0DI071YK05 of Pla... ____ 0.0
emb|CR623949.1| full-length cDNA clone CS0DE014YH06 of Pla... ____ 0.0
emb|CR622930.1| full-length cDNA clone CS0DE004YD03 of Pla... ____ 0.0
emb|CR622697.1| full-length cDNA clone CS0DL002YH12 of B c... ____ 0.0
emb|CR622347.1| full-length cDNA clone CS0DI069YJ19 of Pla... ____ 0.0
emb|CR622131.1| full-length cDNA clone CS0DI075YJ21 of Pla... ____ 0.0
emb|CR620187.1| full-length cDNA clone CS0DE001YC16 of Pla... ____ 0.0
emb|CR619845.1| full-length cDNA clone CS0DI058YC16 of Pla... ____ 0.0
emb|CR618372.1| full-length cDNA clone CS0DD008YF03 of Neu... ____ 0.0
emb|CR618336.1| full-length cDNA clone CS0DC008YA18 of Neu... ____ 0.0
emb|CR618182.1| full-length cDNA clone CS0DG002YE04 of B c... ____ 0.0
emb|CR616905.1| full-length cDNA clone CS0DI065YB06 of Pla... ____ 0.0

**Branchiostoma floridae** [lancelets] taxid 7739
ref|XM_002590712.1| Branchiostoma floridae hypothetical pr... ____ 0.0

**Ornithoctonus huwena** (Chinese golden earth tiger, ...) [spiders] taxid 29017
gb|EU979484.1| Ornithoctonus huwena clone HWE499 60S ribos... ____ 0.0

**Gasterosteus aculeatus** (three spined stickleback) [bony fishes] taxid 69293
gb|BT026683.1| Gasterosteus aculeatus clone CEC39-G07 mRNA... ____ 0.0

**synthetic construct** [other sequences] taxid 32630
gb|EU831455.1| Synthetic construct Homo sapiens clone HAIB... ____ 0.0
gb|EU831542.1| Synthetic construct Homo sapiens clone HAIB... ____ 0.0
gb|HQ447707.1| Synthetic construct Homo sapiens clone IMAG... ____ 0.0
dbj|AB529025.1| Synthetic construct DNA, clone: pF1KB3388,... ____ 0.0

**Pan troglodytes** [primates] taxid 9598
ref|XM_001172807.1| PREDICTED: Pan troglodytes similar to ... ____ 0.0
ref|XM_001167468.1| PREDICTED: Pan troglodytes similar to ... ____ 0.0
ref|XM_001172844.1| PREDICTED: Pan troglodytes similar to ... ____ 0.0
gb|AC191221.3| Pan troglodytes BAC clone CH251-653E5 from ... ____ 0.0
ref|XM_001172799.1| PREDICTED: Pan troglodytes similar to ... ____ 0.0
ref|XM_001172778.1| PREDICTED: Pan troglodytes similar to ... ____ 0.0
ref|XM_001172856.1| PREDICTED: Pan troglodytes similar to ... ____ 0.0
ref|XM_512821.2| PREDICTED: Pan troglodytes similar to rib... ____ 0.0
ref|XM_001172865.1| PREDICTED: Pan troglodytes similar to ... ____ 0.0
ref|XM_511050.2| PREDICTED: Pan troglodytes similar to 23 ... ____ 0.0
ref|XM_001167494.1| PREDICTED: Pan troglodytes similar to ... ____ 0.0
ref|XM_001154037.1| PREDICTED: Pan troglodytes similar to ... ____ 0.0
ref|XM_001154104.1| PREDICTED: Pan troglodytes similar to ... ____ 0.0

**Xenopus (Silurana) tropicalis** [frogs & toads] taxid 8364
gb|BC168117.1| Xenopus tropicalis hypothetical protein LOC... ____ 0.0
gb|BC168111.1| Xenopus tropicalis hypothetical protein LOC... ____ 0.0
gb|BC160997.1| Xenopus tropicalis hypothetical protein LOC... ____ 0.0
gb|BC135476.1| Xenopus tropicalis hypothetical protein LOC... ____ 0.0
ref|NM_001016486.2| Xenopus (Silurana) tropicalis ribosoma... ____ 0.0
emb|CR760846.2| Xenopus tropicalis finished cDNA, clone TT... ____ 0.0

**Bos taurus** (cow, ...) [even-toed ungulates] taxid 9913
ref|NM_001076998.1| Bos taurus ribosomal protein L13a (RPL... ____ 0.0
gb|BC103039.1| Bos taurus ribosomal protein L13a, mRNA (cD... ____ 0.0
emb|X56933.1| B.taurus mRNA, alternative polyadenylation s... ____ 0.0

**Ictalurus furcatus** [bony fishes] taxid 66913
gb|HP440486.1| TSA: Ictalurus furcatus Contig11132.Icfu mR... ____ 0.0

**Monodelphis domestica** [marsupials] taxid 13616
ref|XM_001363184.1| PREDICTED: Monodelphis domestica hypot... ____ 0.0

**Platichthys flesus** [bony fishes] taxid 8260
emb|AJ843089.1| Platichthys flesus mRNA for 60S ribosomal ... ____ 0.0

**Sus scrofa** (wild boar, ...) [even-toed ungulates] taxid 9823

```
ref|XM_003127345.1| PREDICTED: Sus scrofa bcl-2-like prote...     ____   0.0
ref|XM_003127305.1| PREDICTED: Sus scrofa ribosomal protei...     ____   0.0

 Callithrix jacchus (common marmoset, ...) [primates] taxid 9483
ref|XM_002762346.1| PREDICTED: Callithrix jacchus 60S ribo...     ____   0.0
ref|XM_002762345.1| PREDICTED: Callithrix jacchus 60S ribo...     ____   0.0

 Lycosa singoriensis [spiders] taxid 434756
gb|EU247111.1| Lycosa singoriensis 60S ribosomal protein L...     ____   0.0

 Macaca fascicularis (cynomolgus monkey, ...) [primates] taxid 9541
dbj|AB170257.1| Macaca fascicularis brain cDNA clone: QbsB...     ____   0.0
dbj|AB168305.1| Macaca fascicularis testis cDNA clone: Qts...     ____   0.0

 Macaca mulatta (rhesus macaque, ...) [primates] taxid 9544
ref|NM_001195419.1| Macaca mulatta ribosomal protein L13a ...     ____   0.0

 Ailuropoda melanoleuca [carnivores] taxid 9646
ref|XM_002917840.1| PREDICTED: Ailuropoda melanoleuca 60S ...     ____   0.0

 Pongo abelii (Orang-utan, ...) [primates] taxid 9601
ref|XM_002825864.1| PREDICTED: Pongo abelii 60S ribosomal ...     ____   0.0
ref|XM_002825863.1| PREDICTED: Pongo abelii 60S ribosomal ...     ____   0.0
ref|XM_002825862.1| PREDICTED: Pongo abelii 60S ribosomal ...     ____   0.0
ref|XM_002825861.1| PREDICTED: Pongo abelii 60S ribosomal ...     ____   0.0
ref|XM_002825860.1| PREDICTED: Pongo abelii 60S ribosomal ...     ____   0.0
ref|XM_002825859.1| PREDICTED: Pongo abelii 60S ribosomal ...     ____   0.0
ref|XM_002825858.1| PREDICTED: Pongo abelii 60S ribosomal ...     ____   0.0
ref|XM_002825857.1| PREDICTED: Pongo abelii 60S ribosomal ...     ____   0.0
ref|XM_002825856.1| PREDICTED: Pongo abelii 60S ribosomal ...     ____   0.0

 Equus caballus (equine, ...) [odd-toed ungulates] taxid 9796
ref|XM_001491876.2| PREDICTED: Equus caballus similar to r...     ____   0.0
```

---

**Taxonomy Report**

```
root ..................................................   103 hits   39 orgs
. Metazoa .............................................    99 hits   38 orgs [cellular organisms; Eukaryota; Fungi/Metazoa group]
. . Eumetazoa .........................................    97 hits   36 orgs
. . . Cnidaria ........................................     3 hits    3 orgs
. . . . Hexacorallia ..................................     2 hits    2 orgs [Anthozoa]
. . . . . Acropora millepora ..........................     1 hits    1 orgs [Scleractinia; Astrocoeniina; Acroporidae; Acropora]
. . . . . Nematostella vectensis ......................     1 hits    1 orgs [Actiniaria; Edwardsiidae; Nematostella]
. . . . Hydra magnipapillata ..........................     1 hits    1 orgs [Hydrozoa; Hydroida; Anthomedusae; Hydridae; Hydra]
. . . Bilateria .......................................    94 hits   33 orgs
. . . . Coelomata .....................................    92 hits   31 orgs
. . . . . Protostomia .................................     5 hits    5 orgs
. . . . . . Eurythoe complanata .......................     1 hits    1 orgs [Annelida/Echiura/Pogonophora group; Annelida; Polychaeta; Palpata; Ac
. . . . . . Arthropoda ................................     4 hits    4 orgs [Panarthropoda]
. . . . . . . Endopterygota ...........................     2 hits    2 orgs [Mandibulata; Pancrustacea; Hexapoda; Insecta; Dicondylia; Pterygota; 
. . . . . . . . Tribolium castaneum ...................     1 hits    1 orgs [Coleoptera; Polyphaga; Cucujiformia; Tenebrionoidea; Tenebrionidae; T
. . . . . . . . Nasonia vitripennis ...................     1 hits    1 orgs [Hymenoptera; Apocrita; Chaldicoidea group; Chalcidoidea; Pteromalidae
. . . . . . . Araneae .................................     2 hits    2 orgs [Chelicerata; Arachnida]
. . . . . . . . Ornithoctonus huwena ..................     1 hits    1 orgs [Mygalomorphae; Theraphosidae; Ornithoctonus]
. . . . . . . . Lycosa singoriensis ...................     1 hits    1 orgs [Araneomorphae; Entelegynae; RTA clade; Lycosoidea; Lycosidae; Lycosa]
. . . . . Deuterostomia ...............................    87 hits   26 orgs
. . . . . . Chordata ..................................    85 hits   25 orgs
. . . . . . . Euteleostomi ............................    83 hits   23 orgs [Craniata; Vertebrata; Gnathostomata; Teleostomi]
. . . . . . . . Tetrapoda .............................    66 hits   15 orgs [Sarcopterygii]
. . . . . . . . . Amniota .............................    58 hits   13 orgs
. . . . . . . . . . Neognathae ........................     8 hits    2 orgs [Sauropsida; Sauria; Archosauria; Dinosauria; Saurischia; Theropoda; C
. . . . . . . . . . . Taeniopygia guttata .............     7 hits    1 orgs [Passeriformes; Passeroidea; Estrildidae; Estrildinae; Taeniopygia]
. . . . . . . . . . . Gallus gallus ...................     1 hits    1 orgs [Galliformes; Phasianidae; Phasianinae; Gallus]
. . . . . . . . . . Theria ............................    50 hits   11 orgs [Mammalia]
. . . . . . . . . . . Eutheria ........................    49 hits   10 orgs
. . . . . . . . . . . . Simiiformes ...................    42 hits    6 orgs [Euarchontoglires; Primates; Haplorrhini]
. . . . . . . . . . . . . Catarrhini ..................    40 hits    5 orgs
. . . . . . . . . . . . . . Hominidae .................    37 hits    3 orgs [Hominoidea]
. . . . . . . . . . . . . . . Homininae ...............    28 hits    2 orgs
. . . . . . . . . . . . . . . . Homo sapiens ..........    15 hits    1 orgs [Homo]
. . . . . . . . . . . . . . . . Pan troglodytes .......    13 hits    1 orgs [Pan]
. . . . . . . . . . . . . . . Pongo abelii ............     9 hits    1 orgs [Ponginae; Pongo]
. . . . . . . . . . . . . . Macaca ....................     3 hits    2 orgs [Cercopithecoidea; Cercopithecidae; Cercopithecinae]
. . . . . . . . . . . . . . . Macaca fascicularis .....     2 hits    1 orgs
. . . . . . . . . . . . . . . Macaca mulatta ..........     1 hits    1 orgs
. . . . . . . . . . . . . Callithrix jacchus ..........     2 hits    1 orgs [Platyrrhini; Cebidae; Callitrichinae; Callithrix]
. . . . . . . . . . . . Laurasiatheria ................     7 hits    4 orgs
. . . . . . . . . . . . . Cetartiodactyla .............     5 hits    2 orgs
. . . . . . . . . . . . . . Bos taurus ................     3 hits    1 orgs [Ruminantia; Pecora; Bovidae; Bovinae; Bos]
. . . . . . . . . . . . . . Sus scrofa ................     2 hits    1 orgs [Suina; Suidae; Sus]
. . . . . . . . . . . . . Ailuropoda melanoleuca ......     1 hits    1 orgs [Carnivora; Caniformia; Ursidae; Ailuropoda]
. . . . . . . . . . . . . Equus caballus ..............     1 hits    1 orgs [Perissodactyla; Equidae; Equus; Equus subg. Equus]
. . . . . . . . . . . Monodelphis domestica ...........     1 hits    1 orgs [Metatheria; Didelphimorphia; Didelphidae; Didelphinae; Monodelphis]
. . . . . . . . . Xenopus .............................     8 hits    2 orgs [Amphibia; Batrachia; Anura; Mesobatrachia; Pipoidea; Pipidae; Xenopod
. . . . . . . . . . Xenopus laevis ....................     2 hits    1 orgs [Xenopus]
. . . . . . . . . . Xenopus (Silurana) tropicalis .....     6 hits    1 orgs [Silurana]
. . . . . . . . Clupeocephala .........................    17 hits    8 orgs [Actinopterygii; Actinopteri; Neopterygii; Teleostei; Elopocephala]
. . . . . . . . . Euteleostei .........................    16 hits    7 orgs
. . . . . . . . . . Salmoninae ........................    11 hits    3 orgs [Protacanthopterygii; Salmoniformes; Salmonoidei; Salmonidae]
. . . . . . . . . . . Oncorhynchus ....................     2 hits    2 orgs
. . . . . . . . . . . . Oncorhynchus masou formosanus .     1 hits    1 orgs [Oncorhynchus masou]
. . . . . . . . . . . . Oncorhynchus mykiss ...........     1 hits    1 orgs
. . . . . . . . . . . Salmo salar .....................     9 hits    1 orgs [Salmo]
. . . . . . . . . . Percomorpha .......................     5 hits    4 orgs [Neognathi; Neoteleostei; Eurypterygii; Ctenosquamata; Acanthomorpha; 
. . . . . . . . . . . Anoplopoma fimbria ..............     1 hits    1 orgs [Scorpaeniformes; Anoplopomatoidei; Anoplopomatidae; Anoplopoma]
. . . . . . . . . . . Pleuronectiformes ...............     3 hits    2 orgs
. . . . . . . . . . . . Solea senegalensis ............     2 hits    1 orgs [Soleoidei; Soleidae; Solea]
. . . . . . . . . . . . Platichthys flesus ............     1 hits    1 orgs [Pleuronectoidei; Pleuronectidae; Pleuronectinae; Platichthys]
. . . . . . . . . . . Gasterosteus aculeatus ..........     1 hits    1 orgs [Smegmamorpha; Gasterosteiformes/Syngnathiformes group; Gasterosteifor
. . . . . . . . . Ictalurus furcatus ..................     1 hits    1 orgs [Otocephala; Ostariophysi; Otophysi; Siluriphysi; Siluriformes; Ictalu
. . . . . . . Ciona intestinalis ......................     1 hits    1 orgs [Tunicata; Ascidiacea; Enterogona; Phlebobranchia; Cionidae; Ciona]
. . . . . . . Branchiostoma floridae ..................     1 hits    1 orgs [Cephalochordata; Branchiostomidae; Branchiostoma]
. . . . . . Strongylocentrotus purpuratus .............     2 hits    1 orgs [Echinodermata; Eleutherozoa; Echinozoa; Echinoidea; Euechinoidea; Ech
. . . . Schistosoma ...................................     2 hits    2 orgs [Acoelomata; Platyhelminthes; Trematoda; Digenea; Strigeidida; Schisto
. . . . . Schistosoma mansoni .........................     1 hits    1 orgs
. . . . . Schistosoma japonicum .......................     1 hits    1 orgs
. . Trichoplax adhaerens ..............................     1 hits    1 orgs [Placozoa; Trichoplax]
. . Suberites domuncula ...............................     1 hits    1 orgs [Porifera; Demospongiae; Tetractinomorpha; Hadromerida; Suberitidae; S
. synthetic construct .................................     4 hits    1 orgs [other sequences; artificial sequences]
```

**BLAST**

# Basic Local Alignment Search Tool

Jump to Page Content

**Job Title: EZ028666**

- 

## Tax BLAST Report

**Index**


- Lineage Report
- Organism Report
- Taxonomy Report
- Help


### Lineage Report

```
root
. Metazoa        [animals]
. . Eumetazoa      [animals]
. . . Hexacorallia  [anthozoans]
. . . . Astrocoeniina [stony corals]
. . . . . Acropora millepora ------------------       1 hit  [stony corals]        TSA: Acropora millepora SeqIndex5112, mRNA sequence
. . . . . Stylophora pistillata ...............       1 hit  [stony corals]        Stylophora pistillata 60S acidic ribosomal phosphoprotein P
. . . . Nematostella vectensis ----------------       1 hit  [sea anemones]        Nematostella vectensis predicted protein (NEMVEDRAFT_v1g237
. . . Gallus gallus (bantam) ------------------       2 hits [birds]               Gallus gallus ribosomal protein, large, P0 (RPLP0), mRNA >g
. . . Taeniopygia guttata (zebra finch) .......       1 hit  [birds]               PREDICTED: Taeniopygia guttata similar to RPLP0 protein (LO
. . . Monodelphis domestica ...................       1 hit  [marsupials]          PREDICTED: Monodelphis domestica similar to 60S acidic ribo
. . . Dermacentor variabilis ..................       2 hits [mites & ticks]       TSA: Dermacentor variabilis contig00110.Dvb, mRNA sequence
. . . Solea senegalensis ......................       1 hit  [bony fishes]         Solea senegalensis RPLP0 mRNA for ribosomal protein LP0, co
. . . Ixodes scapularis (shoulder tick) .......       2 hits [mites & ticks]       Ixodes scapularis isolate ISUFL22 60S acidic ribosomal prot
. . . Microcosmus squamiger ...................       1 hit  [tunicates]           Microcosmus squamiger mRNA for ribosomal protein, large, P0
. . . Hypophthalmichthys molitrix .............       1 hit  [bony fishes]         Hypophthalmichthys molitrix large ribosomal protein P0 mRNA
. . . Osmerus mordax ..........................       1 hit  [bony fishes]         Osmerus mordax clone omor-rgc-511-099 60S acidic ribosomal
. . . Bos taurus (cow) ........................       4 hits [even-toed ungulates] Bos taurus ribosomal protein, large, P0, mRNA (cDNA clone M
. . . Danio rerio (zebra fish) ................       2 hits [bony fishes]         Danio rerio ribosomal protein, large, P0, mRNA (cDNA clone
. . . Xenopus laevis (common platanna) ........       2 hits [frogs & toads]       Xenopus laevis ribosomal protein, large, P0 (rplp0), mRNA >
. . . Homo sapiens (man) ......................      32 hits [primates]            Homo sapiens cDNA clone IMAGE:3506537, **** WARNING: chimer
. . . Pan troglodytes .........................       1 hit  [primates]            PREDICTED: Pan troglodytes similar to 60S acidic ribosomal
. . . Pongo abelii (Orang-utan) ...............       1 hit  [primates]            PREDICTED: Pongo abelii 60S acidic ribosomal protein P0-lik
. . . Callithrix jacchus (common marmoset) ....       2 hits [primates]            PREDICTED: Callithrix jacchus 60S acidic ribosomal protein
. . . Macaca mulatta (rhesus macaque) .........       1 hit  [primates]            Macaca mulatta ribosomal protein, large, P0 (RPLP0), mRNA
. . . Macaca fascicularis (cynomolgus monkey) .       1 hit  [primates]            Macaca fascicularis brain cDNA clone: QbsB-10454, similar t
. . . Equus caballus (equine) .................       2 hits [odd-toed ungulates]  PREDICTED: Equus caballus similar to ribosomal phosphoprote
. . . Xenopus (Silurana) tropicalis ...........       2 hits [frogs & toads]       Xenopus (Silurana) tropicalis ribosomal protein, large, P0
. . . Canis lupus familiaris (dogs) ...........      10 hits [carnivores]          PREDICTED: Canis familiaris similar to acidic ribosomal pho
. . . Sus scrofa (wild boar) ..................       6 hits [even-toed ungulates] Sus scrofa mRNA, clone:THY010103A05, expressed in thymus
. . . Ictalurus furcatus ......................       1 hit  [bony fishes]         TSA: Ictalurus furcatus Contig10804.Icfu mRNA sequence
. . . Rattus norvegicus (brown rat) ...........      18 hits [rodents]             Rattus norvegicus TL0AEA68YG13 mRNA sequence
. . . Rana sylvatica ..........................       1 hit  [frogs & toads]       Rana sylvatica brain acidic ribosomal phosphoprotein P0 mRN
. . Trichoplax adhaerens ----------------------       1 hit  [placozoans]          Trichoplax adhaerens expressed hypothetical protein, mRNA
. synthetic construct -------------------------       3 hits [other sequences]     Synthetic construct Homo sapiens clone IMAGE:100009456; FLH
```

---

### Organism Report

```
 Acropora millepora [stony corals] taxid 45264
gb|EZ028666.1| TSA: Acropora millepora SeqIndex5112, mRNA ...       0.0

 Stylophora pistillata [stony corals] taxid 50429
gb|EU069460.1| Stylophora pistillata 60S acidic ribosomal ...       0.0

 Nematostella vectensis [sea anemones] taxid 45351
ref|XM_001626244.1| Nematostella vectensis predicted prote...       0.0

 Gallus gallus (bantam, ...) [birds] taxid 9031
ref|NM_204987.1| Gallus gallus ribosomal protein, large, P...       0.0
gb|L28704.1|CHKACRIPHO Chicken acidic ribosomal phosphopro...       0.0

 Taeniopygia guttata (zebra finch) [birds] taxid 59729
ref|XM_002199525.1| PREDICTED: Taeniopygia guttata similar...       0.0

 Monodelphis domestica [marsupials] taxid 13616
ref|XM_001363500.1| PREDICTED: Monodelphis domestica simil...       0.0

 Dermacentor variabilis [mites & ticks] taxid 34621
gb|EZ525048.1| TSA: Dermacentor variabilis contig00110.Dvb...       0.0
gb|EZ532706.1| TSA: Dermacentor variabilis contig00067.Dvc...       0.0

 Solea senegalensis [bony fishes] taxid 28829
dbj|AB374983.1| Solea senegalensis RPLP0 mRNA for ribosoma...       0.0

 Ixodes scapularis (shoulder tick, ...) [mites & ticks] taxid 6945
gb|DQ066213.1| Ixodes scapularis isolate ISUFL22 60S acidi...       0.0
ref|XM_002399569.1| Ixodes scapularis 60S acidic ribosomal...       0.0

 Microcosmus squamiger [tunicates] taxid 439822
emb|FN984781.1| Microcosmus squamiger mRNA for ribosomal p...       0.0

 Hypophthalmichthys molitrix [bony fishes] taxid 13095
gb|HM124745.1| Hypophthalmichthys molitrix large ribosomal...       0.0

 Osmerus mordax [bony fishes] taxid 8014
gb|BT074646.1| Osmerus mordax clone omor-rgc-511-099 60S a...       0.0

 Bos taurus (cow, ...) [even-toed ungulates] taxid 9913
gb|BC151695.1| Bos taurus ribosomal protein, large, P0, mR...       0.0
gb|BC102074.1| Bos taurus ribosomal protein, large, P0, mR...       0.0
ref|NM_001012682.1| Bos taurus ribosomal protein, large, P...       0.0
gb|BT021080.1| Bos taurus ribosomal protein, large, P0 (RP...       0.0

 Danio rerio (zebra fish, ...) [bony fishes] taxid 7955
gb|BC062854.1| Danio rerio ribosomal protein, large, P0, m...       0.0
```

| | | | |
|---|---|---|---|
| gb\|BC146738.1\| | Danio rerio ribosomal protein, large, P0, m... | | 0.0 |

**Xenopus laevis** (common platanna, ...) [frogs & toads] taxid 8355

| | | | |
|---|---|---|---|
| ref\|NM_001086665.1\| | Xenopus laevis ribosomal protein, larg... | | 0.0 |
| gb\|BC042268.1\| | Xenopus laevis acidic ribosomal protein P0,... | | 0.0 |

**Homo sapiens** (man) [primates] taxid 9606

| | | | |
|---|---|---|---|
| gb\|BC003075.1\| | Homo sapiens cDNA clone IMAGE:3506537, ****... | | 0.0 |
| gb\|BC071877.1\| | Homo sapiens cDNA clone IMAGE:5087581, ****... | | 0.0 |
| dbj\|AK001313.1\| | Homo sapiens cDNA FLJ10451 fis, clone NT2R... | | 0.0 |
| gb\|BC021049.1\| | Homo sapiens cDNA clone IMAGE:3505381, ****... | | 0.0 |
| gb\|BC033190.1\| | Homo sapiens ribosomal protein, large, P0, ... | | 0.0 |
| gb\|BC071911.1\| | Homo sapiens cDNA clone IMAGE:6569344, ****... | | 0.0 |
| ref\|NM_053275.3\| | Homo sapiens ribosomal protein, large, P0... | | 0.0 |
| ref\|NM_001002.3\| | Homo sapiens ribosomal protein, large, P0... | | 0.0 |
| gb\|BC019014.2\| | Homo sapiens ribosomal protein, large, P0, ... | | 0.0 |
| gb\|BC003655.2\| | Homo sapiens ribosomal protein, large, P0, ... | | 0.0 |
| emb\|CR625036.1\| | full-length cDNA clone CL0BB022ZB08 of Neu... | | 0.0 |
| emb\|CR597824.1\| | full-length cDNA clone CL0BA004ZA12 of Pla... | | 0.0 |
| emb\|CR619416.1\| | full-length cDNA clone CS0DH004YL14 of T c... | | 0.0 |
| emb\|CR613873.1\| | full-length cDNA clone CS0DH004YP23 of T c... | | 0.0 |
| emb\|CR625189.1\| | full-length cDNA clone CS0DF026YD05 of Fet... | | 0.0 |
| gb\|BC015690.1\| | Homo sapiens ribosomal protein, large, P0, ... | | 0.0 |
| emb\|CR600402.1\| | full-length cDNA clone CS0DN002YE06 of Adu... | | 0.0 |
| dbj\|AK129823.1\| | Homo sapiens cDNA FLJ26313 fis, clone DMC0... | | 0.0 |
| gb\|BC000087.2\| | Homo sapiens ribosomal protein, large, P0, ... | | 0.0 |
| gb\|BC001834.2\| | Homo sapiens ribosomal protein, large, P0, ... | | 0.0 |
| gb\|BC000752.2\| | Homo sapiens ribosomal protein, large, P0, ... | | 0.0 |
| gb\|M17885.1\|HUMPPARP0 | Human acidic ribosomal phosphoprotei... | | 0.0 |
| dbj\|AK129754.1\| | Homo sapiens cDNA FLJ26243 fis, clone DMC0... | | 0.0 |
| gb\|BC000345.2\| | Homo sapiens ribosomal protein, large, P0, ... | | 0.0 |
| gb\|BC005863.2\| | Homo sapiens ribosomal protein, large, P0, ... | | 0.0 |
| gb\|BC009867.2\| | Homo sapiens ribosomal protein, large, P0, ... | | 0.0 |
| gb\|BC008092.1\| | Homo sapiens ribosomal protein, large, P0, ... | | 0.0 |
| gb\|BC008594.1\| | Homo sapiens ribosomal protein, large, P0, ... | | 0.0 |
| emb\|CR602521.1\| | full-length cDNA clone CS0DA002YE14 of Neu... | | 0.0 |
| gb\|BC015173.1\| | Homo sapiens ribosomal protein, large, P0, ... | | 0.0 |
| emb\|CR599043.1\| | full-length cDNA clone CS0DH006YP10 of T c... | | 0.0 |
| dbj\|AK222468.1\| | Homo sapiens mRNA for ribosomal protein P0... | | 0.0 |

**Pan troglodytes** [primates] taxid 9598

| | | | |
|---|---|---|---|
| ref\|XM_509423.2\| | PREDICTED: Pan troglodytes similar to 60S... | | 0.0 |

**Pongo abelii** (Orang-utan, ...) [primates] taxid 9601

| | | | |
|---|---|---|---|
| ref\|XM_002823848.1\| | PREDICTED: Pongo abelii 60S acidic rib... | | 0.0 |

**Callithrix jacchus** (common marmoset, ...) [primates] taxid 9483

| | | | |
|---|---|---|---|
| ref\|XM_002753072.1\| | PREDICTED: Callithrix jacchus 60S acid... | | 0.0 |
| ref\|XM_002753073.1\| | PREDICTED: Callithrix jacchus 60S acid... | | 0.0 |

**Macaca mulatta** (rhesus macaque, ...) [primates] taxid 9544

| | | | |
|---|---|---|---|
| ref\|NM_001195428.1\| | Macaca mulatta ribosomal protein, larg... | | 0.0 |

**Macaca fascicularis** (cynomolgus monkey, ...) [primates] taxid 9541

| | | | |
|---|---|---|---|
| dbj\|AB170149.1\| | Macaca fascicularis brain cDNA clone: QbsB... | | 0.0 |

**synthetic construct** [other sequences] taxid 32630

| | | | |
|---|---|---|---|
| gb\|DQ894996.2\| | Synthetic construct Homo sapiens clone IMAG... | | 0.0 |
| gb\|DQ891813.2\| | Synthetic construct clone IMAGE:100004443; ... | | 0.0 |
| dbj\|AB464171.1\| | Synthetic construct DNA, clone: pF1KB8219,... | | 0.0 |

**Equus caballus** (equine, ...) [odd-toed ungulates] taxid 9796

| | | | |
|---|---|---|---|
| ref\|XM_001489353.1\| | PREDICTED: Equus caballus similar to r... | | 0.0 |
| ref\|XM_001489330.1\| | PREDICTED: Equus caballus similar to r... | | 0.0 |

**Xenopus (Silurana) tropicalis** [frogs & toads] taxid 8364

| | | | |
|---|---|---|---|
| ref\|NM_203736.1\| | Xenopus (Silurana) tropicalis ribosomal p... | | 0.0 |
| gb\|BC061299.1\| | Xenopus tropicalis acidic ribosomal phospho... | | 0.0 |

**Canis lupus familiaris** (dogs) [carnivores] taxid 9615

| | | | |
|---|---|---|---|
| ref\|XM_857926.1\| | PREDICTED: Canis familiaris similar to ac... | | 0.0 |
| ref\|XM_857980.1\| | PREDICTED: Canis familiaris similar to ac... | | 0.0 |
| ref\|XM_844092.1\| | PREDICTED: Canis familiaris ribosomal pro... | | 0.0 |
| ref\|XM_858027.1\| | PREDICTED: Canis familiaris similar to ac... | | 0.0 |
| ref\|XM_858052.1\| | PREDICTED: Canis familiaris similar to ac... | | 0.0 |
| ref\|XM_858004.1\| | PREDICTED: Canis familiaris similar to ac... | | 0.0 |
| ref\|XM_535894.2\| | PREDICTED: Canis familiaris ribosomal pro... | | 0.0 |
| ref\|XM_853158.1\| | PREDICTED: Canis familiaris ribosomal pro... | | 0.0 |
| ref\|XM_853112.1\| | PREDICTED: Canis familiaris ribosomal pro... | | 0.0 |
| ref\|XM_845901.1\| | PREDICTED: Canis familiaris similar to ac... | | 0.0 |

**Sus scrofa** (wild boar, ...) [even-toed ungulates] taxid 9823

| | | | |
|---|---|---|---|
| dbj\|AK239428.1\| | Sus scrofa mRNA, clone:THY010103A05, expre... | | 0.0 |
| ref\|NM_001098598.1\| | Sus scrofa ribosomal phosphoprotein la... | | 0.0 |
| gb\|DQ316319.1\| | Sus scrofa ribosomal phosphoprotein large P... | | 0.0 |
| dbj\|AK347722.1\| | Sus scrofa mRNA, clone:OVR010082B07, expre... | | 0.0 |
| dbj\|AK231505.1\| | Sus scrofa mRNA, clone:ITT010083C10, expre... | | 0.0 |
| dbj\|AK234557.1\| | Sus scrofa mRNA, clone:OVR010084G10, expre... | | 0.0 |

**Ictalurus furcatus** [bony fishes] taxid 66913

| | | | |
|---|---|---|---|
| gb\|HP440171.1\| | TSA: Ictalurus furcatus Contig10804.Icfu mR... | | 0.0 |

**Rattus norvegicus** (brown rat, ...) [rodents] taxid 10116

| | | | |
|---|---|---|---|
| emb\|FQ231733.1\| | Rattus norvegicus TL0AEA68YG13 mRNA sequence | | 0.0 |
| emb\|FQ231525.1\| | Rattus norvegicus TL0AEA6YC21 mRNA sequence | | 0.0 |
| emb\|FQ229397.1\| | Rattus norvegicus TL0ADA48YB05 mRNA sequence | | 0.0 |
| emb\|FQ220533.1\| | Rattus norvegicus TL0ADA42YI10 mRNA sequence | | 0.0 |
| emb\|FQ225860.1\| | Rattus norvegicus TL0AEA3YO11 mRNA sequence | | 0.0 |
| emb\|FQ231591.1\| | Rattus norvegicus TL0AEA68YP07 mRNA sequence | | 0.0 |
| emb\|FQ220542.1\| | Rattus norvegicus TL0ADA42YH23 mRNA sequence | | 0.0 |
| emb\|FQ220527.1\| | Rattus norvegicus TL0ADA42YI17 mRNA sequence | | 0.0 |
| emb\|FQ220282.1\| | Rattus norvegicus TL0ADA43YH18 mRNA sequence | | 0.0 |
| emb\|FQ220008.1\| | Rattus norvegicus TL0ADA44YI12 mRNA sequence | | 0.0 |
| emb\|FQ212927.1\| | Rattus norvegicus TL0AAA50YK12 mRNA sequence | | 0.0 |
| emb\|FQ229408.1\| | Rattus norvegicus TL0ADA48YA13 mRNA sequence | | 0.0 |
| emb\|FQ229273.1\| | Rattus norvegicus TL0ADA48YH23 mRNA sequence | | 0.0 |
| emb\|FQ228995.1\| | Rattus norvegicus TL0ADA4YM19 mRNA sequence | | 0.0 |
| emb\|FQ231967.1\| | Rattus norvegicus TL0AEA67YI08 mRNA sequence | | 0.0 |

```
emb|FQ224846.1| Rattus norvegicus TL0ACA52YN20 mRNA sequence       ____  0.0
emb|FQ227941.1| Rattus norvegicus TL0AEA11YC10 mRNA sequence       ____  0.0
emb|FQ233377.1| Rattus norvegicus TL0AEA62YC06 mRNA sequence       ____  0.0

 Rana sylvatica [frogs & toads] taxid 45438
gb|AF176302.1| Rana sylvatica brain acidic ribosomal phosp...      ____  0.0

 Trichoplax adhaerens [placozoans] taxid 10228
ref|XM_002114895.1| Trichoplax adhaerens expressed hypothe...      ____  0.0
```

---

**Taxonomy Report**

```
root .........................................   105 hits   30 orgs
. Metazoa ....................................   102 hits   29 orgs [cellular organisms; Eukaryota; Fungi/Metazoa group]
. . Eumetazoa ................................   101 hits   28 orgs
. . . Hexacorallia ...........................     3 hits    3 orgs [Cnidaria; Anthozoa]
. . . . Astrocoeniina ........................     2 hits    2 orgs [Scleractinia]
. . . . . Acropora millepora .................     1 hits    1 orgs [Acroporidae; Acropora]
. . . . . Stylophora pistillata ..............     1 hits    1 orgs [Pocilloporidae; Stylophora]
. . . . Nematostella vectensis ...............     1 hits    1 orgs [Actiniaria; Edwardsiidae; Nematostella]
. . . Coelomata ..............................    98 hits   25 orgs [Bilateria]
. . . . Chordata .............................    94 hits   23 orgs [Deuterostomia]
. . . . . Euteleostomi .......................    93 hits   22 orgs [Craniata; Vertebrata; Gnathostomata; Teleostomi]
. . . . . . Tetrapoda ........................    87 hits   17 orgs [Sarcopterygii]
. . . . . . . Amniota ........................    82 hits   14 orgs
. . . . . . . . Neognathae ...................     3 hits    2 orgs [Sauropsida; Sauria; Archosauria; Dinosauria; Saurischia; Theropoda; Coeluro
. . . . . . . . . Gallus gallus ..............     2 hits    1 orgs [Galliformes; Phasianidae; Phasianinae; Gallus]
. . . . . . . . . Taeniopygia guttata ........     1 hits    1 orgs [Passeriformes; Passeroidea; Estrildidae; Estrildinae; Taeniopygia]
. . . . . . . . Theria .......................    79 hits   12 orgs [Mammalia]
. . . . . . . . . Monodelphis domestica ......     1 hits    1 orgs [Metatheria; Didelphimorphia; Didelphidae; Didelphinae; Monodelphis]
. . . . . . . . . Eutheria ...................    78 hits   11 orgs
. . . . . . . . . . Laurasiatheria ...........    22 hits    4 orgs
. . . . . . . . . . . Cetartiodactyla ........    10 hits    2 orgs
. . . . . . . . . . . . Bos taurus ...........     4 hits    1 orgs [Ruminantia; Pecora; Bovidae; Bovinae; Bos]
. . . . . . . . . . . . Sus scrofa ...........     6 hits    1 orgs [Suina; Suidae; Sus]
. . . . . . . . . . . Equus caballus .........     2 hits    1 orgs [Perissodactyla; Equidae; Equus; Equus subg. Equus]
. . . . . . . . . . . Canis lupus familiaris .    10 hits    1 orgs [Carnivora; Caniformia; Canidae; Canis; Canis lupus]
. . . . . . . . . . Euarchontoglires .........    56 hits    7 orgs
. . . . . . . . . . . Simiiformes ............    38 hits    6 orgs [Primates; Haplorrhini]
. . . . . . . . . . . . Catarrhini ...........    36 hits    5 orgs
. . . . . . . . . . . . . Hominidae ..........    34 hits    3 orgs [Hominoidea]
. . . . . . . . . . . . . . Homininae ........    33 hits    2 orgs
. . . . . . . . . . . . . . . Homo sapiens ...    32 hits    1 orgs [Homo]
. . . . . . . . . . . . . . . Pan troglodytes     1 hits    1 orgs [Pan]
. . . . . . . . . . . . . . Pongo abelii .....     1 hits    1 orgs [Ponginae; Pongo]
. . . . . . . . . . . . . Macaca .............     2 hits    2 orgs [Cercopithecoidea; Cercopithecidae; Cercopithecinae]
. . . . . . . . . . . . . . Macaca mulatta ...     1 hits    1 orgs
. . . . . . . . . . . . . . Macaca fascicularis     1 hits    1 orgs
. . . . . . . . . . . . Callithrix jacchus ...     2 hits    1 orgs [Platyrrhini; Cebidae; Callitrichinae; Callithrix]
. . . . . . . . . . . Rattus norvegicus ......    18 hits    1 orgs [Glires; Rodentia; Sciurognathi; Muroidea; Muridae; Murinae; Rattus]
. . . . . . . Anura ..........................     5 hits    3 orgs [Amphibia; Batrachia]
. . . . . . . . Xenopus ......................     4 hits    2 orgs [Mesobatrachia; Pipoidea; Pipidae; Xenopodinae]
. . . . . . . . . Xenopus laevis .............     2 hits    1 orgs [Xenopus]
. . . . . . . . . Xenopus (Silurana) tropicalis    2 hits    1 orgs [Silurana]
. . . . . . . . Rana sylvatica ...............     1 hits    1 orgs [Neobatrachia; Ranoidea; Ranidae; Raninae; Rana; Novirana; Aquarana/sylvatic
. . . . . . Clupeocephala ....................     6 hits    5 orgs [Actinopterygii; Actinopteri; Neopterygii; Teleostei; Elopocephala]
. . . . . . . Euteleostei ....................     2 hits    2 orgs
. . . . . . . . Solea senegalensis ...........     1 hits    1 orgs [Neognathi; Neoteleostei; Eurypterygii; Ctenosquamata; Acanthomorpha; Euacan
. . . . . . . . Osmerus mordax ...............     1 hits    1 orgs [Protacanthopterygii; Osmeriformes; Osmeroidei; Osmeroidea; Osmeridae; Osmer
. . . . . . . Otophysi .......................     4 hits    3 orgs [Otocephala; Ostariophysi]
. . . . . . . . Cyprinidae ...................     3 hits    2 orgs [Cypriniphysi; Cypriniformes; Cyprinoidea]
. . . . . . . . . Hypophthalmichthys molitrix      1 hits    1 orgs [Hypophthalmichthys]
. . . . . . . . . Danio rerio ................     2 hits    1 orgs [Danio]
. . . . . . . . Ictalurus furcatus ...........     1 hits    1 orgs [Siluriphysi; Siluriformes; Ictaluridae; Ictalurus]
. . . . . Microcosmus squamiger ..............     1 hits    1 orgs [Tunicata; Ascidiacea; Stolidobranchia; Pyuridae; Microcosmus]
. . . . Ixodidae .............................     4 hits    2 orgs [Protostomia; Panarthropoda; Arthropoda; Chelicerata; Arachnida; Acari; Para
. . . . . Dermacentor variabilis .............     2 hits    1 orgs [Rhipicephalinae; Dermacentor]
. . . . . Ixodes scapularis ..................     2 hits    1 orgs [Ixodinae; Ixodes]
. . Trichoplax adhaerens .....................     1 hits    1 orgs [Placozoa; Trichoplax]
. synthetic construct ........................     3 hits    1 orgs [other sequences; artificial sequences]
```

## BLAST

# Basic Local Alignment Search Tool

Jump to Page Content

**Job Title: EZ011917**

---

- 

### Tax BLAST Report

**Index**

- Lineage Report
- Organism Report
- Taxonomy Report
- Help

**Lineage Report**

```
root
. Eumetazoa    [animals]
. . Cnidaria     [cnidarians]
. . . Hexacorallia [anthozoans]
. . . . Acropora millepora ------------------------------     6 hits [stony corals]        TSA: Acropora millepora SeqIndex13789, mRNA sequence
. . . . Nematostella vectensis ..........................     1 hit  [sea anemones]        Nematostella vectensis predicted protein (NEMVEDRAFT
. . . Hydra magnipapillata ------------------------------     2 hits [hydrozoans]          PREDICTED: Hydra magnipapillata similar to bcl-2-lik
. . . Hydra vulgaris ....................................     1 hit  [hydrozoans]          Hydra vulgaris bcl-2-like 4 mRNA, complete cds
. . Saccoglossus kowalevskii ----------------------------     1 hit  [hemichordates]       PREDICTED: Saccoglossus kowalevskii predicted Bcl-2
. . Xenopus (Silurana) tropicalis .......................     2 hits [frogs & toads]       Xenopus (Silurana) tropicalis BCL2-like 2 (bcl2l2),
. . Xenopus laevis (common platanna) ....................     4 hits [frogs & toads]       Xenopus laevis BCL2-like 2 (bcl2l2-a), mRNA >gi|4911
. . Oryctolagus cuniculus (domestic rabbit) .............     1 hit  [rabbits & hares]     PREDICTED: Oryctolagus cuniculus BCL-WEL-like (LOC10
. . Odontesthes bonariensis .............................     1 hit  [bony fishes]         Odontesthes bonariensis bcl2 mRNA, complete cds
. . Canis lupus familiaris (dogs) .......................     5 hits [carnivores]          PREDICTED: Canis familiaris BCL2-like 2, transcript
. . Sus scrofa (wild boar) ..............................     2 hits [even-toed ungulates] Sus scrofa mRNA, clone:PCT010006D06, expressed in pl
. . Homo sapiens (man) ..................................    13 hits [primates]            Homo sapiens mRNA similar to BCL2-like 2 (cDNA clone
. . Danio rerio (zebra fish) ............................     7 hits [bony fishes]         Danio rerio Bcl-xL-like protein 1 (blp1) mRNA, compl
. . Pongo abelii (Orang-utan) ...........................     2 hits [primates]            PREDICTED: Pongo abelii bcl-2-like protein 2-like, t
. . Callithrix jacchus (common marmoset) ................     3 hits [primates]            PREDICTED: Callithrix jacchus bcl-2-like protein 2-l
. . Bos taurus (cow) ....................................     7 hits [even-toed ungulates] Bos taurus BCL2-like 2 (BCL2L2), mRNA >gi|92096661|g
. . Equus caballus (equine) .............................     1 hit  [odd-toed ungulates]  PREDICTED: Equus caballus similar to Apoptosis regul
. . Ailuropoda melanoleuca ..............................     1 hit  [carnivores]          PREDICTED: Ailuropoda melanoleuca bcl-2-like protein
. . Felis catus (cat) ...................................     1 hit  [carnivores]          Felis catus BCL2L2 (BCL2L2) mRNA, partial cds
. . Gobio gobio .........................................     1 hit  [bony fishes]         Gobio gobio bcl-2 protein mRNA, partial cds
. . Monodelphis domestica ...............................     4 hits [marsupials]          PREDICTED: Monodelphis domestica similar to ENSANGP0
. . Rattus norvegicus (brown rat) .......................     7 hits [rodents]             Rattus norvegicus TL0AAA72YG18 mRNA sequence
. . Mus musculus (mouse) ................................    14 hits [rodents]             Mus musculus adult male lung cDNA, RIKEN full-length
. . Macaca mulatta (rhesus macaque) .....................     6 hits [primates]            PREDICTED: Macaca mulatta polyadenylate-binding prot
. . Ornithorhynchus anatinus (duck-billed platypus) .....     1 hit  [monotremes]          PREDICTED: Ornithorhynchus anatinus similar to Bcl-w
. . Gadus morhua ........................................     1 hit  [bony fishes]         Gadus morhua Bcl-X2 (Bcl-X2) mRNA, partial cds
. . Lonchura striata (white-backed munia) ...............     1 hit  [birds]               Lonchura striata Bcl-xL (Bcl-xL) mRNA, complete cds
. . Cricetulus longicaudatus (lesser long-tailed ham...) .    2 hits [rodents]             Cricetulus longicaudatus mRNA for B-cell lymphoma 2
. . Taeniopygia guttata (zebra finch) ...................     1 hit  [birds]               PREDICTED: Taeniopygia guttata BCL2-like 1 (LOC10022
. . Gallus gallus (bantam) ..............................     2 hits [birds]               Gallus gallus BCL2-like 1 (BCL2L1), nuclear gene enc
. . Pan troglodytes .....................................     1 hit  [primates]            PREDICTED: Pan troglodytes BCL2-like 1, transcript v
. . Macaca fascicularis (cynomolgus monkey) .............     1 hit  [primates]            Macaca fascicularis mRNA, clone QnpA-16956: similar
. synthetic construct -----------------------------------     7 hits [other sequences]     Synthetic construct DNA, clone: pF1KSDA0271, Homo sa
```

---

**Organism Report**

```
 Acropora millepora [stony corals] taxid 45264
gb|EZ011917.1| TSA: Acropora millepora SeqIndex13789, mRNA...       0.0
gb|EU161957.1| Acropora millepora Bcl-like protein mRNA, c...       0.0
gb|EZ004016.1| TSA: Acropora millepora SeqIndex13791, mRNA...       0.0
gb|EZ004015.1| TSA: Acropora millepora SeqIndex13790, mRNA...       0.0
gb|EU161958.1| Acropora millepora Bax-like protein mRNA, c...       0.0
gb|EZ011916.1| TSA: Acropora millepora SeqIndex13788, mRNA...       0.0

 Nematostella vectensis [sea anemones] taxid 45351
ref|XM_001634647.1| Nematostella vectensis predicted prote...       0.0

 Hydra magnipapillata [hydrozoans] taxid 6085
ref|XM_002167542.1| PREDICTED: Hydra magnipapillata simila...       0.0
ref|XM_002158467.1| PREDICTED: Hydra magnipapillata simila...       0.0

 Hydra vulgaris [hydrozoans] taxid 6087
gb|EU035764.1| Hydra vulgaris bcl-2-like 4 mRNA, complete cds       0.0

 Saccoglossus kowalevskii [hemichordates] taxid 10224
ref|XM_002740743.1| PREDICTED: Saccoglossus kowalevskii pr...       0.0

 Xenopus (Silurana) tropicalis [frogs & toads] taxid 8364
ref|NM_001011065.1| Xenopus (Silurana) tropicalis BCL2-lik...       0.0
gb|BC084445.1| Xenopus tropicalis Bcl2-like 2, mRNA (cDNA ...       0.0

 Xenopus laevis (common platanna, ...) [frogs & toads] taxid 8355
ref|NM_001092260.1| Xenopus laevis BCL2-like 2 (bcl2l2-a),...       0.0
gb|BC073259.1| Xenopus laevis MGC80617 protein, mRNA (cDNA...       0.0
ref|NM_001088104.1| Xenopus laevis BCL2-like 2 (bcl2l2-b),...       0.0
emb|X82462.1| X.laevis R1 mRNA                                      0.0

 Oryctolagus cuniculus (domestic rabbit, ...) [rabbits & hares] taxid 9986
ref|XM_002717903.1| PREDICTED: Oryctolagus cuniculus BCL-W...       0.0

 Odontesthes bonariensis [bony fishes] taxid 219752
gb|FJ868843.1| Odontesthes bonariensis bcl2 mRNA, complete...       0.0

 Canis lupus familiaris (dogs) [carnivores] taxid 9615
ref|XM_853671.1| PREDICTED: Canis familiaris BCL2-like 2, ...       0.0
ref|XM_853783.1| PREDICTED: Canis familiaris BCL2-like 2, ...       0.0
ref|NM_001031635.2| Canis lupus familiaris poly(A) binding...       0.0
gb|DQ116955.1| Canis familiaris Bcl-w protein (BCL2L2) mRN...       0.0
gb|AY509563.1| Canis familiaris Bcl-2 mRNA, complete cds            0.0

 Sus scrofa (wild boar, ...) [even-toed ungulates] taxid 9823
dbj|AK349051.1| Sus scrofa mRNA, clone:PCT010006D06, expre...       0.0
```

ref|XM_001928880.2| PREDICTED: Sus scrofa bcl-2-like prote... 0.0

**Homo sapiens** (man) [primates] taxid 9606
gb|BC011637.1| Homo sapiens mRNA similar to BCL2-like 2 (c... 0.0
ref|NM_004050.3| Homo sapiens BCL2-like 2 (BCL2L2), mRNA 0.0
dbj|D87461.1| Homo sapiens mRNA for KIAA0271 gene, partial... 0.0
gb|BC113522.1| Homo sapiens BCL2-like 2, mRNA (cDNA clone ... 0.0
gb|BC104789.1| Homo sapiens BCL2-like 2, mRNA (cDNA clone ... 0.0
gb|BC021198.2| Homo sapiens BCL2-like 2, mRNA (cDNA clone ... 0.0
dbj|AK289519.1| Homo sapiens cDNA FLJ75568 complete cds, h... 0.0
gb|BT019549.1| Homo sapiens BCL2-like 2 mRNA, complete cds 0.0
gb|U59747.1|HSU59747 Human Bcl-w (bcl-w) mRNA, complete cds 0.0
emb|AL049829.4| Human chromosome 14 DNA sequence BAC R-124... 0.0
gb|AY927500.1| Homo sapiens mRNA sequence 0.0
ref|NM_138578.1| Homo sapiens BCL2-like 1 (BCL2L1), nuclea... 0.0
gb|BC019307.1| Homo sapiens BCL2-like 1, mRNA (cDNA clone ... 0.0

**Danio rerio** (zebra fish, ...) [bony fishes] taxid 7955
gb|DQ022560.1| Danio rerio Bcl-xL-like protein 1 (blp1) mR... 0.0
gb|BC071291.1| Danio rerio bcl2-like, mRNA (cDNA clone MGC... 0.0
ref|NM_131807.1| Danio rerio bcl2-like 1 (bcl2l1), nuclear... 0.0
gb|AF317837.1|AF317837 Danio rerio Bcl-XL-like protein 1 (... 0.0
gb|BC044130.1| Danio rerio bcl2-like, mRNA (cDNA clone MGC... 0.0
gb|BC165156.1| Danio rerio bcl2-like, mRNA (cDNA clone MGC... 0.0
ref|XM_001341178.2| PREDICTED: Danio rerio Bcl2-like (LOC1... 0.0

**Pongo abelii** (Orang-utan, ...) [primates] taxid 9601
ref|XM_002824582.1| PREDICTED: Pongo abelii bcl-2-like pro... 0.0
ref|XM_002824583.1| PREDICTED: Pongo abelii bcl-2-like pro... 0.0

**Callithrix jacchus** (common marmoset, ...) [primates] taxid 9483
ref|XM_002753638.1| PREDICTED: Callithrix jacchus bcl-2-li... 0.0
ref|XM_002747332.1| PREDICTED: Callithrix jacchus bcl-2-li... 0.0
ref|XM_002747333.1| PREDICTED: Callithrix jacchus bcl-2-li... 0.0

**Bos taurus** (cow, ...) [even-toed ungulates] taxid 9913
ref|NM_001076533.1| Bos taurus BCL2-like 2 (BCL2L2), mRNA 0.0
gb|BC114652.1| Bos taurus BCL2-like 2, mRNA (cDNA clone MG... 0.0
dbj|AB238938.1| Bos taurus BCL2L2 mRNA for BCL2-like 2, co... 0.0
gb|DQ001758.1| Bos taurus BCL2-like protein 2 (BCL2L2) gen... 0.0
gb|BC147863.1| Bos taurus BCL2-like 1, mRNA (cDNA clone MG... 0.0
ref|NM_001077486.2| Bos taurus BCL2-like 1 (BCL2L1), nucle... 0.0
gb|BC133281.1| Bos taurus BCL2-like 1, mRNA (cDNA clone MG... 0.0

**Equus caballus** (equine, ...) [odd-toed ungulates] taxid 9796
ref|XM_001493694.2| PREDICTED: Equus caballus similar to A... 0.0

**Ailuropoda melanoleuca** [carnivores] taxid 9646
ref|XM_002926013.1| PREDICTED: Ailuropoda melanoleuca bcl-... 0.0

**Felis catus** (cat, ...) [carnivores] taxid 9685
gb|DQ926874.1| Felis catus BCL2L2 (BCL2L2) mRNA, partial cds 0.0

**synthetic construct** [other sequences] taxid 32630
dbj|AB383828.1| Synthetic construct DNA, clone: pF1KSDA027... 0.0
gb|BT019548.1| Synthetic construct Homo sapiens BCL2-like ... 0.0
gb|AY890819.1| Synthetic construct Homo sapiens clone FLH0... 0.0
gb|AY890433.1| Synthetic construct Homo sapiens clone FLH1... 0.0
gb|AY888229.1| Synthetic construct Homo sapiens clone FLH0... 0.0
gb|AY892894.1| Synthetic construct Homo sapiens clone FLH1... 0.0
gb|AY888228.1| Synthetic construct Homo sapiens clone FLH0... 0.0

**Gobio gobio** [bony fishes] taxid 27704
gb|FJ612582.1| Gobio gobio bcl-2 protein mRNA, partial cds 0.0

**Monodelphis domestica** [marsupials] taxid 13616
ref|XM_001369257.1| PREDICTED: Monodelphis domestica simil... 0.0
ref|XM_001365223.1| PREDICTED: Monodelphis domestica simil... 0.0
ref|XM_001365293.1| PREDICTED: Monodelphis domestica simil... 0.0
ref|XM_001362813.1| PREDICTED: Monodelphis domestica simil... 0.0

**Rattus norvegicus** (brown rat, ...) [rodents] taxid 10116
emb|FQ212018.1| Rattus norvegicus TL0AAA72YG18 mRNA sequence 0.0
ref|NM_021850.2| Rattus norvegicus Bcl2-like 2 (Bcl2l2), mRNA 0.0
gb|BC074021.1| Rattus norvegicus Bcl2-like 2, mRNA (cDNA c... 0.0
gb|AY185098.1| Rattus norvegicus BCL-W mRNA, complete cds 0.0
gb|AY185100.1| Rattus norvegicus BCL-WEL mRNA, complete cds 0.0
gb|AF096291.1|AF096291 Rattus norvegicus Bcl-w (bcl-w) mRN... 0.0
gb|AF512835.1| Rattus norvegicus Bcl2-like protein mRNA, c... 0.0

**Mus musculus** (mouse) [rodents] taxid 10090
dbj|AK004680.1| Mus musculus adult male lung cDNA, RIKEN f... 0.0
ref|NM_007537.1| Mus musculus BCL2-like 2 (Bcl2l2), mRNA 0.0
gb|AF030769.1|AF030769 Mus musculus BCL-W (Bcl-w) mRNA, co... 0.0
dbj|AK172925.1| Mus musculus mRNA for mKIAA0271 protein 0.0
dbj|AK015644.1| Mus musculus adult male testis cDNA, RIKEN... 0.0
gb|U59746.1|MMU59746 Mus musculus Bcl-w (bcl-w) mRNA, comp... 0.0
gb|AC116591.4| Mus musculus BAC clone RP24-90K1 from chrom... 0.0
emb|CT025533.11| Mouse DNA sequence from clone RP23-93K3 o... 0.0
gb|BC040369.1| Mus musculus BCL2-like 2, mRNA (cDNA clone ... 0.0
dbj|AK013244.1| Mus musculus 10, 11 days embryo whole body... 0.0
ref|NM_009741.3| Mus musculus B-cell leukemia/lymphoma 2 (... 0.0
gb|BC095964.1| Mus musculus B-cell leukemia/lymphoma 2, mR... 0.0
dbj|AK049473.1| Mus musculus 7 days embryo whole body cDNA... 0.0
dbj|AK172250.1| Mus musculus activated spleen cDNA, RIKEN ... 0.0

**Macaca mulatta** (rhesus macaque, ...) [primates] taxid 9544
ref|XM_001107287.2| PREDICTED: Macaca mulatta polyadenylat... 0.0
ref|XM_001110326.1| PREDICTED: Macaca mulatta bcl-2-like p... 0.0
ref|XM_001110285.1| PREDICTED: Macaca mulatta bcl-2-like p... 0.0
ref|XM_001110113.2| PREDICTED: Macaca mulatta bcl-2-like p... 0.0
ref|XM_001110205.2| PREDICTED: Macaca mulatta bcl-2-like p... 0.0
ref|XM_001110062.2| PREDICTED: Macaca mulatta bcl-2-like p... 0.0

**Ornithorhynchus anatinus** (duck-billed platypus, ...) [monotremes] taxid 9258
ref|XM_001512417.1| PREDICTED: Ornithorhynchus anatinus si... 0.0

**Gadus morhua** [bony fishes] taxid 8049

```
gb|GQ387053.1| Gadus morhua Bcl-X2 (Bcl-X2) mRNA, partial cds        0.0

 Lonchura striata (white-backed munia) [birds] taxid 40157
gb|DQ017953.1| Lonchura striata Bcl-xL (Bcl-xL) mRNA, comp...        0.0

 Cricetulus longicaudatus (lesser long-tailed hamster, ...) [rodents] taxid 10030
emb|AJ271720.1| Cricetulus longicaudatus mRNA for B-cell l...        0.0
gb|AF404339.1|AF404339 Cricetulus longicaudatus B-cell lym...        0.0

 Taeniopygia guttata (zebra finch) [birds] taxid 59729
ref|XM_002193086.1| PREDICTED: Taeniopygia guttata BCL2-li...        0.0

 Gallus gallus (bantam, ...) [birds] taxid 9031
ref|NM_001025304.1| Gallus gallus BCL2-like 1 (BCL2L1), nu...        0.0
gb|U26645.1|GGU26645 Gallus gallus bcl-x (bcl-x) mRNA, com...        0.0

 Pan troglodytes [primates] taxid 9598
ref|XM_514565.2| PREDICTED: Pan troglodytes BCL2-like 1, t...        0.0

 Macaca fascicularis (cynomolgus monkey, ...) [primates] taxid 9541
dbj|AB220511.1| Macaca fascicularis mRNA, clone QnpA-16956...        0.0
```

---

**Taxonomy Report**

```
root .............................................  110 hits   33 orgs
. Eumetazoa ......................................  103 hits   32 orgs [cellular organisms; Eukaryota; Fungi/Metazoa group; Metazoa]
. . Cnidaria .....................................   10 hits    4 orgs
. . . Hexacorallia ...............................    7 hits    2 orgs [Anthozoa]
. . . . Acropora millepora .......................    6 hits    1 orgs [Scleractinia; Astrocoeniina; Acroporidae; Acropora]
. . . . Nematostella vectensis ...................    1 hits    1 orgs [Actiniaria; Edwardsiidae; Nematostella]
. . . Hydra ......................................    3 hits    2 orgs [Hydrozoa; Hydroida; Anthomedusae; Hydridae]
. . . . Hydra magnipapillata .....................    2 hits    1 orgs
. . . . Hydra vulgaris ...........................    1 hits    1 orgs
. . Deuterostomia ................................   93 hits   28 orgs [Bilateria; Coelomata]
. . . Saccoglossus kowalevskii ...................    1 hits    1 orgs [Hemichordata; Enteropneusta; Harrimaniidae; Saccoglossus]
. . . Euteleostomi ...............................   92 hits   27 orgs [Chordata; Craniata; Vertebrata; Gnathostomata; Teleostomi]
. . . . Tetrapoda ................................   82 hits   23 orgs [Sarcopterygii]
. . . . . Xenopus ................................    6 hits    2 orgs [Amphibia; Batrachia; Anura; Mesobatrachia; Pipoidea; Pipidae; Xenopodinae]
. . . . . . Xenopus (Silurana) tropicalis ........    2 hits    1 orgs [Silurana]
. . . . . . Xenopus laevis .......................    4 hits    1 orgs [Xenopus]
. . . . . Amniota ................................   76 hits   21 orgs
. . . . . . Mammalia .............................   72 hits   18 orgs
. . . . . . . Theria .............................   71 hits   17 orgs
. . . . . . . . Eutheria .........................   67 hits   16 orgs
. . . . . . . . . Euarchontoglires ...............   50 hits   10 orgs
. . . . . . . . . . Glires .......................   24 hits    4 orgs
. . . . . . . . . . . Oryctolagus cuniculus ......    1 hits    1 orgs [Lagomorpha; Leporidae; Oryctolagus]
. . . . . . . . . . . Muroidea ...................   23 hits    3 orgs [Rodentia; Sciurognathi]
. . . . . . . . . . . . Murinae ..................   21 hits    2 orgs [Muridae]
. . . . . . . . . . . . . Rattus norvegicus ......    7 hits    1 orgs [Rattus]
. . . . . . . . . . . . . Mus musculus ...........   14 hits    1 orgs [Mus; Mus]
. . . . . . . . . . . . Cricetulus longicaudatus .    2 hits    1 orgs [Cricetidae; Cricetinae; Cricetulus]
. . . . . . . . . . Simiiformes ..................   26 hits    6 orgs [Primates; Haplorrhini]
. . . . . . . . . . . Catarrhini .................   23 hits    5 orgs
. . . . . . . . . . . . Hominidae ................   16 hits    3 orgs [Hominoidea]
. . . . . . . . . . . . . Homininae ..............   14 hits    2 orgs
. . . . . . . . . . . . . . Homo sapiens .........   13 hits    1 orgs [Homo]
. . . . . . . . . . . . . . Pan troglodytes ......    1 hits    1 orgs [Pan]
. . . . . . . . . . . . . Pongo abelii ...........    2 hits    1 orgs [Ponginae; Pongo]
. . . . . . . . . . . . Macaca ...................    7 hits    2 orgs [Cercopithecoidea; Cercopithecidae; Cercopithecinae]
. . . . . . . . . . . . . Macaca mulatta .........    6 hits    1 orgs
. . . . . . . . . . . . . Macaca fascicularis ....    1 hits    1 orgs
. . . . . . . . . . . Callithrix jacchus .........    3 hits    1 orgs [Platyrrhini; Cebidae; Callitrichinae; Callithrix]
. . . . . . . . . Laurasiatheria .................   17 hits    6 orgs
. . . . . . . . . . Carnivora ....................    7 hits    3 orgs
. . . . . . . . . . . Caniformia .................    6 hits    2 orgs
. . . . . . . . . . . . Canis lupus familiaris ...    5 hits    1 orgs [Canidae; Canis; Canis lupus]
. . . . . . . . . . . . Ailuropoda melanoleuca ...    1 hits    1 orgs [Ursidae; Ailuropoda]
. . . . . . . . . . . Felis catus ................    1 hits    1 orgs [Feliformia; Felidae; Felinae; Felis]
. . . . . . . . . . Cetartiodactyla ..............    9 hits    2 orgs
. . . . . . . . . . . Sus scrofa .................    2 hits    1 orgs [Suina; Suidae; Sus]
. . . . . . . . . . . Bos taurus .................    7 hits    1 orgs [Ruminantia; Pecora; Bovidae; Bovinae; Bos]
. . . . . . . . . . Equus caballus ...............    1 hits    1 orgs [Perissodactyla; Equidae; Equus; Equus subg. Equus]
. . . . . . . . Monodelphis domestica ............    4 hits    1 orgs [Metatheria; Didelphimorphia; Didelphidae; Didelphinae; Monodelphis]
. . . . . . . Ornithorhynchus anatinus ...........    1 hits    1 orgs [Prototheria; Monotremata; Ornithorhynchidae; Ornithorhynchus]
. . . . . . Neognathae ...........................    4 hits    3 orgs [Sauropsida; Sauria; Archosauria; Dinosauria; Saurischia; Theropoda; Coelur
. . . . . . . Estrildinae ........................    2 hits    2 orgs [Passeriformes; Passeroidea; Estrildidae]
. . . . . . . . Lonchura striata .................    1 hits    1 orgs [Lonchura]
. . . . . . . . Taeniopygia guttata ..............    1 hits    1 orgs [Taeniopygia]
. . . . . . . Gallus gallus ......................    2 hits    1 orgs [Galliformes; Phasianidae; Phasianinae; Gallus]
. . . . Clupeocephala ............................   10 hits    4 orgs [Actinopterygii; Actinopteri; Neopterygii; Teleostei; Elopocephala]
. . . . . Holacanthopterygii .....................    2 hits    2 orgs [Euteleostei; Neognathi; Neoteleostei; Eurypterygii; Ctenosquamata; Acanthor
. . . . . . Odontesthes bonariensis ..............    1 hits    1 orgs [Acanthopterygii; Euacanthopterygii; Percomorpha; Smegmamorpha; Atherinomor
. . . . . . Gadus morhua .........................    1 hits    1 orgs [Paracanthopterygii; Gadiformes; Gadidae; Gadus]
. . . . . Cyprinidae .............................    8 hits    2 orgs [Otocephala; Ostariophysi; Otophysi; Cypriniphysi; Cypriniformes; Cyprinoid
. . . . . . Danio rerio ..........................    7 hits    1 orgs [Danio]
. . . . . . Gobio gobio ..........................    1 hits    1 orgs [Gobio]
. synthetic construct ............................    7 hits    1 orgs [other sequences; artificial sequences]
```

# BLAST

## Basic Local Alignment Search Tool

Jump to Page Content

**Job Title: (2) - EZ037140**

- 

### Tax BLAST Report

**Index**


- Lineage Report
- Organism Report
- Taxonomy Report
- Help


**Lineage Report**

```
root
. Metazoa       [animals]
. . Eumetazoa     [animals]
. . . Cnidaria      [cnidarians]
. . . . Hexacorallia [anthozoans]
. . . . . Acropora millepora ------------------------      1 hit  [stony corals]        TSA: Acropora millepora SeqIndex7167, mRNA sequence
. . . . . Nematostella vectensis ....................      1 hit  [sea anemones]        Nematostella vectensis predicted protein (NEMVEDRAFT v1g1
. . . . Hydra magnipapillata ------------------------      2 hits [hydrozoans]          PREDICTED: Hydra magnipapillata similar to bak (LOC100208
. . . . Hydra vulgaris ..............................      2 hits [hydrozoans]          Hydra vulgaris bak mRNA, complete cds
. . . Branchiostoma floridae ------------------------      1 hit  [lancelets]           Branchiostoma floridae hypothetical protein, mRNA
. . . Strongylocentrotus purpuratus (purple urchin) .      2 hits [sea urchins]         PREDICTED: Strongylocentrotus purpuratus similar to Bak p
. . . Monodelphis domestica .........................      1 hit  [marsupials]          PREDICTED: Monodelphis domestica similar to Bcl-2 antagon
. . . Gallus gallus (bantam) ........................      3 hits [birds]               Gallus gallus BCL2-antagonist/killer 1 (BAK1), mRNA >gi|6
. . . Ailuropoda melanoleuca ........................      2 hits [carnivores]          PREDICTED: Ailuropoda melanoleuca bcl-2 homologous antago
. . . Callithrix jacchus (common marmoset) ..........      4 hits [primates]            PREDICTED: Callithrix jacchus bcl-2 homologous antagonist
. . . Canis lupus familiaris (dogs) .................      2 hits [carnivores]          Canis lupus familiaris BCL2-antagonist/killer 1 (BAK1), m
. . . Oryctolagus cuniculus (domestic rabbit) .......      1 hit  [rabbits & hares]     PREDICTED: Oryctolagus cuniculus BCL2-antagonist/killer 1
. . . Ovis aries (domestic sheep) ...................      1 hit  [even-toed ungulates] Ovis aries Bak protein mRNA, partial cds
. . . Homo sapiens (man) ............................     22 hits [primates]            Homo sapiens BCL2-antagonist/killer 1 (BAK1), mRNA
. . . Pan troglodytes ...............................      4 hits [primates]            PREDICTED: Pan troglodytes BCL2-antagonist/killer 1, tran
. . . Macaca mulatta (rhesus macaque) ...............      8 hits [primates]            PREDICTED: Macaca mulatta BCL2-antagonist/killer 1, trans
. . . Bos taurus (cow) ..............................      9 hits [even-toed ungulates] Bos taurus BCL2-antagonist/killer 1 (BAK1), mRNA, complet
. . . Sus scrofa (wild boar) ........................      2 hits [even-toed ungulates] PREDICTED: Sus scrofa Bak protein (BAK), mRNA
. . . Patiria miniata ...............................      1 hit  [starfish]            TSA: Patiria miniata isotig20647.Pminagast mRNA sequence
. . . Equus caballus (equine) .......................      2 hits [odd-toed ungulates]  PREDICTED: Equus caballus similar to Bcl-2 antagonist/kil
. . . Pongo abelii (Orang-utan) .....................      3 hits [primates]            PREDICTED: Pongo abelii bcl-2 homologous antagonist/kille
. . . Mus musculus (mouse) ..........................      6 hits [rodents]             Mus musculus BCL2-antagonist/killer 1 (Bak1), mRNA
. . . Rattus norvegicus (brown rat) .................      3 hits [rodents]             Rattus norvegicus BCL2-antagonist/killer 1 (Bak1), mRNA >
. . . Cricetulus griseus (Chinese hamsters) .........      2 hits [rodents]             Cricetulus griseus Bak mRNA, complete cds
. . . Xenopus (Silurana) tropicalis .................      1 hit  [frogs & toads]       PREDICTED: Xenopus (Silurana) tropicalis bcl-2 homologous
. . . Xenopus laevis (common platanna) ..............      2 hits [frogs & toads]       Xenopus laevis BCL2-antagonist/killer 1 (bak1), mRNA >gi|
. . . Saccoglossus kowalevskii ......................      1 hit  [hemichordates]       PREDICTED: Saccoglossus kowalevskii predicted Bcl-2 prote
. . . Saimiri boliviensis ...........................      1 hit  [primates]            Saimiri boliviensis BCL2-like protein 1 (Bcl2L1) mRNA, co
. . . Aotus vociferans (Spix's night monkey) ........      1 hit  [primates]            Aotus vociferans BCL2-like protein 1 (Bcl2L1) mRNA, compl
. . . Taeniopygia guttata (zebra finch) .............      1 hit  [birds]               PREDICTED: Taeniopygia guttata BCL2-like 1 (LOC100224900)
. . . Felis catus (cat) .............................      1 hit  [carnivores]          Felis catus BCL2L1 (BCL2L1) mRNA, partial cds
. . . Spermophilus tridecemlineatus .................      1 hit  [rodents]             Spermophilus tridecemlineatus Bcl-XL (Bcl-XL) mRNA, parti
. . Trichoplax adhaerens ----------------------------      1 hit  [placozoans]          Trichoplax adhaerens expressed hypothetical protein, mRNA
. synthetic construct -------------------------------     11 hits [other sequences]     Synthetic construct Homo sapiens clone IMAGE:100010199; F
```

---

**Organism Report**

```
 Acropora millepora [stony corals] taxid 45264
gb|EZ037140.1| TSA: Acropora millepora SeqIndex7167, mRNA ...          0.0

 Nematostella vectensis [sea anemones] taxid 45351
ref|XM_001641905.1| Nematostella vectensis predicted prote...          0.0

 Branchiostoma floridae [lancelets] taxid 7739
ref|XM_002589394.1| Branchiostoma floridae hypothetical pr...          0.0

 Hydra magnipapillata [hydrozoans] taxid 6085
ref|XM_002160789.1| PREDICTED: Hydra magnipapillata simila...          0.0
ref|XM_002167542.1| PREDICTED: Hydra magnipapillata simila...          0.0

 Hydra vulgaris [hydrozoans] taxid 6087
gb|EF104645.1| Hydra vulgaris bak mRNA, complete cds                   0.0
gb|EU035764.1| Hydra vulgaris bcl-2-like 4 mRNA, complete cds          0.0

 Strongylocentrotus purpuratus (purple urchin, ...) [sea urchins] taxid 7668
ref|XM_001204408.1| PREDICTED: Strongylocentrotus purpurat...          0.0
ref|XM_788104.1| PREDICTED: Strongylocentrotus purpuratus ...          0.0

 Monodelphis domestica [marsupials] taxid 13616
ref|XM_001377739.1| PREDICTED: Monodelphis domestica simil...          0.0

 Gallus gallus (bantam, ...) [birds] taxid 9031
ref|NM_001030920.1| Gallus gallus BCL2-antagonist/killer 1...          0.0
emb|AJ851496.1| Gallus gallus mRNA for hypothetical protei...          0.0
gb|AC161468.2| Gallus gallus BAC clone CH261-94J19 from ch...          0.0

 Ailuropoda melanoleuca [carnivores] taxid 9646
ref|XM_002914323.1| PREDICTED: Ailuropoda melanoleuca bcl-...          0.0
ref|XM_002918283.1| PREDICTED: Ailuropoda melanoleuca bcl-...          0.0

 Callithrix jacchus (common marmoset, ...) [primates] taxid 9483
ref|XM_002746436.1| PREDICTED: Callithrix jacchus bcl-2 ho...          0.0
ref|XM_002747334.1| PREDICTED: Callithrix jacchus bcl-2-li...          0.0
ref|XM_002747333.1| PREDICTED: Callithrix jacchus bcl-2-li...          0.0
ref|XM_002747332.1| PREDICTED: Callithrix jacchus bcl-2-li...          0.0

 Canis lupus familiaris (dogs) [carnivores] taxid 9615
ref|NM_001020808.1| Canis lupus familiaris BCL2-antagonist...          0.0
gb|DQ002813.1| Canis familiaris Bcl-2 antagonist/killer pr...          0.0

 Oryctolagus cuniculus (domestic rabbit, ...) [rabbits & hares] taxid 9986
```

ref|XM_002714580.1| PREDICTED: Oryctolagus cuniculus BCL2-... 0.0

**Ovis aries** (domestic sheep, ...) [even-toed ungulates] taxid 9940
gb|AF164518.1|AF164518 Ovis aries Bak protein mRNA, partia... 0.0

**Homo sapiens** (man) [primates] taxid 9606
ref|NM_001188.3| Homo sapiens BCL2-antagonist/killer 1 (BA... 0.0
gb|BC004431.1| Homo sapiens BCL2-antagonist/killer 1, mRNA... 0.0
gb|BC032789.1| Homo sapiens cDNA clone IMAGE:5213984, cont... 0.0
gb|U16811.1|HSU16811 Human Bak mRNA, complete cds 0.0
emb|CR615881.1| full-length cDNA clone CS0DC002YE18 of Neu... 0.0
emb|CR600490.1| full-length cDNA clone CS0DL011YK12 of B c... 0.0
dbj|AK091807.1| Homo sapiens cDNA FLJ34488 fis, clone HLUN... 0.0
emb|CR615932.1| full-length cDNA clone CS0DI010YJ01 of Pla... 0.0
gb|U23765.1|HSU23765 Human Bak protein mRNA, complete cds 0.0
dbj|AK293547.1| Homo sapiens cDNA FLJ55169 complete cds, h... 0.0
emb|X84213.1| H.sapiens BAK mRNA for BCl-2 homologue 0.0
emb|CR457419.1| Homo sapiens full open reading frame cDNA ... 0.0
gb|AF520590.1| Homo sapiens pro-apoptotic protein BAKM var... 0.0
emb|BX640505.3| Human DNA sequence from clone WI2-82760E2 ... 0.0
gb|U16812.1|HSU16812 Human Bak-2 gene, complete cds 0.0
ref|NG_000850.5| Homo sapiens BCL2-antagonist/killer 1 pse... 0.0
ref|XM_002348050.2| PREDICTED: Homo sapiens putative Bcl-2... 0.0
dbj|AK292093.1| Homo sapiens cDNA FLJ75165 complete cds, h... 0.0
gb|BC110337.1| Homo sapiens BCL2-antagonist/killer 1, mRNA... 0.0
emb|CR627020.1| Homo sapiens mRNA; cDNA DKFZp686D0345 (fro... 0.0
gb|AY260471.1| Homo sapiens BCL2-antagonist/killer 1 (BAK1... 0.0
emb|Z93017.6| Human DNA sequence from clone RP1-291J10 on ... 0.0

**Pan troglodytes** [primates] taxid 9598
ref|XM_518407.2| PREDICTED: Pan troglodytes BCL2-antagonis... 0.0
ref|XM_001171325.1| PREDICTED: Pan troglodytes BCL2-antago... 0.0
gb|AC191941.3| Pan troglodytes BAC clone CH251-384C22 from... 0.0
ref|XM_514565.2| PREDICTED: Pan troglodytes BCL2-like 1, t... 0.0

**Macaca mulatta** (rhesus macaque, ...) [primates] taxid 9544
ref|XM_001090570.2| PREDICTED: Macaca mulatta BCL2-antagon... 0.0
ref|XM_002803988.1| PREDICTED: Macaca mulatta BCL2-antagon... 0.0
ref|XM_002803989.1| PREDICTED: Macaca mulatta BCL2-antagon... 0.0
ref|XM_001110113.2| PREDICTED: Macaca mulatta bcl-2-like p... 0.0
ref|XM_001110062.2| PREDICTED: Macaca mulatta bcl-2-like p... 0.0
ref|XM_001110205.2| PREDICTED: Macaca mulatta bcl-2-like p... 0.0
ref|XM_001110326.1| PREDICTED: Macaca mulatta bcl-2-like p... 0.0
ref|XM_001110285.1| PREDICTED: Macaca mulatta bcl-2-like p... 0.0

**synthetic construct** [other sequences] taxid 32630
gb|DQ895739.2| Synthetic construct Homo sapiens clone IMAG... 0.0
gb|DQ892527.2| Synthetic construct clone IMAGE:100005157; ... 0.0
dbj|AB590158.1| Synthetic construct DNA, clone: pFN21AB694... 0.0
gb|AY893853.1| Synthetic construct Homo sapiens clone FLH0... 0.0
gb|AY893403.1| Synthetic construct Homo sapiens clone FLH0... 0.0
gb|AY889585.1| Synthetic construct Homo sapiens clone FLH1... 0.0
dbj|AB528577.1| Synthetic construct DNA, clone: pF1KB6997,... 0.0
emb|CU678721.1| Synthetic construct Homo sapiens gateway c... 0.0
dbj|AB464466.1| Synthetic construct DNA, clone: pF1KB8901,... 0.0
gb|DQ896311.2| Synthetic construct Homo sapiens clone IMAG... 0.0
gb|DQ893461.2| Synthetic construct clone IMAGE:100006091; ... 0.0

**Bos taurus** (cow, ...) [even-toed ungulates] taxid 9913
gb|BT030701.1| Bos taurus BCL2-antagonist/killer 1 (BAK1),... 0.0
gb|BC146049.1| Bos taurus BCL2-antagonist/killer 1, mRNA (... 0.0
ref|NM_001077918.1| Bos taurus BCL2-antagonist/killer 1 (B... 0.0
dbj|AB238939.1| Bos taurus BAK1 mRNA for Bcl-2 homologous ... 0.0
gb|HM630302.1| Bos taurus BCL2-like protein 1 (Bcl2L1) mRN... 0.0
gb|BC147863.1| Bos taurus BCL2-like 1, mRNA (cDNA clone MG... 0.0
ref|NM_001077486.2| Bos taurus BCL2-like 1 (BCL2L1), nucle... 0.0
gb|BC133281.1| Bos taurus BCL2-like 1, mRNA (cDNA clone MG... 0.0
dbj|AB238936.1| Bos taurus BCL2L1 mRNA for BCL2-like 1 tra... 0.0

**Sus scrofa** (wild boar, ...) [even-toed ungulates] taxid 9823
ref|XM_001928147.2| PREDICTED: Sus scrofa Bak protein (BAK... 0.0
emb|CU498854.16| Pig DNA sequence from clone CH242-216B14 ... 0.0

**Patiria miniata** [starfish] taxid 46514
gb|HP125449.1| TSA: Patiria miniata isotig20647.Pminagast ... 0.0

**Equus caballus** (equine, ...) [odd-toed ungulates] taxid 9796
ref|XM_001497824.1| PREDICTED: Equus caballus similar to B... 0.0
ref|XM_001499714.1| PREDICTED: Equus caballus similar to B... 0.0

**Pongo abelii** (Orang-utan, ...) [primates] taxid 9601
ref|XM_002824315.1| PREDICTED: Pongo abelii bcl-2 homologo... 0.0
ref|XM_002816764.1| PREDICTED: Pongo abelii bcl-2 homologo... 0.0
ref|XM_002830190.1| PREDICTED: Pongo abelii bcl-2-like pro... 0.0

**Mus musculus** (mouse) [rodents] taxid 10090
ref|NM_007523.2| Mus musculus BCL2-antagonist/killer 1 (Ba... 0.0
emb|Y13231.1| Mus musculus mRNA for Bak 0.0
dbj|AK089220.1| Mus musculus NOD-derived CD11c +ve dendrit... 0.0
gb|BC057589.1| Mus musculus BCL2-antagonist/killer 1, mRNA... 0.0
gb|AF402617.1|AF402617 Mus musculus N-BAK1 (Bak1) mRNA, co... 0.0
ref|NM_009743.4| Mus musculus BCL2-like 1 (Bcl2l1), nuclea... 0.0

**Rattus norvegicus** (brown rat, ...) [rodents] taxid 10116
ref|NM_053812.1| Rattus norvegicus BCL2-antagonist/killer ... 0.0
gb|AF259504.1| Rattus norvegicus BAK protein (Bak) mRNA, c... 0.0
gb|BC089784.1| Rattus norvegicus BCL2-antagonist/killer 1,... 0.0

**Cricetulus griseus** (Chinese hamsters) [rodents] taxid 10029
gb|EF104644.1| Cricetulus griseus Bak mRNA, complete cds 0.0
gb|EU596590.1| Cricetulus griseus Bcl-xL mRNA, complete cds 0.0

**Xenopus (Silurana) tropicalis** [frogs & toads] taxid 8364
ref|XM_002936222.1| PREDICTED: Xenopus (Silurana) tropical... 0.0

**Xenopus laevis** (common platanna, ...) [frogs & toads] taxid 8355
ref|NM_001096118.1| Xenopus laevis BCL2-antagonist/killer ... 0.0
gb|BC099018.1| Xenopus laevis hypothetical protein MGC1154... 0.0

**Trichoplax adhaerens** [placozoans] taxid 10228
ref|XM_002117760.1| Trichoplax adhaerens expressed hypothe... ____ 0.0

**Saccoglossus kowalevskii** [hemichordates] taxid 10224
ref|XM_002740743.1| PREDICTED: Saccoglossus kowalevskii pr... ____ 0.0

**Saimiri boliviensis** [primates] taxid 27679
gb|HM630305.1| Saimiri boliviensis BCL2-like protein 1 (Bc... ____ 0.0

**Aotus vociferans** (Spix's night monkey) [primates] taxid 57176
gb|HM630303.1| Aotus vociferans BCL2-like protein 1 (Bcl2L... ____ 0.0

**Taeniopygia guttata** (zebra finch) [birds] taxid 59729
ref|XM_002193086.1| PREDICTED: Taeniopygia guttata BCL2-li... ____ 0.0

**Felis catus** (cat, ...) [carnivores] taxid 9685
gb|DQ926873.1| Felis catus BCL2L1 (BCL2L1) mRNA, partial cds ____ 0.0

**Spermophilus tridecemlineatus** [rodents] taxid 43179
gb|DQ503423.1| Spermophilus tridecemlineatus Bcl-XL (Bcl-X... ____ 0.0

---

## Taxonomy Report

```
root ................................................  106 hits   34 orgs
. Metazoa ...........................................   95 hits   33 orgs [cellular organisms; Eukaryota; Fungi/Metazoa group]
. . Eumetazoa .......................................   94 hits   32 orgs
. . . Cnidaria ......................................    6 hits    4 orgs
. . . . Hexacorallia ................................    2 hits    2 orgs [Anthozoa]
. . . . . Acropora millepora ........................    1 hits    1 orgs [Scleractinia; Astrocoeniina; Acroporidae; Acropora]
. . . . . Nematostella vectensis ....................    1 hits    1 orgs [Actiniaria; Edwardsiidae; Nematostella]
. . . . Hydra .......................................    4 hits    2 orgs [Hydrozoa; Hydroida; Anthomedusae; Hydridae]
. . . . . Hydra magnipapillata ......................    2 hits    1 orgs
. . . . . Hydra vulgaris ............................    2 hits    1 orgs
. . . Deuterostomia .................................   88 hits   28 orgs [Bilateria; Coelomata]
. . . . Chordata ....................................   84 hits   25 orgs
. . . . . Branchiostoma floridae ....................    1 hits    1 orgs [Cephalochordata; Branchiostomidae; Branchiostoma]
. . . . . Tetrapoda .................................   83 hits   24 orgs [Craniata; Vertebrata; Gnathostomata; Teleostomi; Euteleostomi; Sarcop
. . . . . . Amniota .................................   80 hits   22 orgs
. . . . . . . Theria ................................   76 hits   20 orgs [Mammalia]
. . . . . . . . Monodelphis domestica ...............    1 hits    1 orgs [Metatheria; Didelphimorphia; Didelphidae; Didelphinae; Monodelphis]
. . . . . . . . Eutheria ............................   75 hits   19 orgs
. . . . . . . . . Laurasiatheria ....................   19 hits    7 orgs
. . . . . . . . . . Carnivora .......................    5 hits    3 orgs
. . . . . . . . . . . Caniformia ....................    4 hits    2 orgs
. . . . . . . . . . . . Ailuropoda melanoleuca .......    2 hits    1 orgs [Ursidae; Ailuropoda]
. . . . . . . . . . . . Canis lupus familiaris .......    2 hits    1 orgs [Canidae; Canis; Canis lupus]
. . . . . . . . . . . Felis catus ...................    1 hits    1 orgs [Feliformia; Felidae; Felinae; Felis]
. . . . . . . . . . Cetartiodactyla .................   12 hits    3 orgs
. . . . . . . . . . . Bovidae .......................   10 hits    2 orgs [Ruminantia; Pecora]
. . . . . . . . . . . . Ovis aries ..................    1 hits    1 orgs [Caprinae; Ovis]
. . . . . . . . . . . . Bos taurus ..................    9 hits    1 orgs [Bovinae; Bos]
. . . . . . . . . . . Sus scrofa ....................    2 hits    1 orgs [Suina; Suidae; Sus]
. . . . . . . . . . Equus caballus ..................    2 hits    1 orgs [Perissodactyla; Equidae; Equus; Equus subg. Equus]
. . . . . . . . . Euarchontoglires ..................   56 hits   12 orgs
. . . . . . . . . . Simiiformes .....................   43 hits    7 orgs [Primates; Haplorrhini]
. . . . . . . . . . . Platyrrhini ...................    6 hits    3 orgs
. . . . . . . . . . . . Cebidae .....................    5 hits    2 orgs
. . . . . . . . . . . . . Callithrix jacchus .........    4 hits    1 orgs [Callitrichinae; Callithrix]
. . . . . . . . . . . . . Saimiri boliviensis ........    1 hits    1 orgs [Saimiriinae; Saimiri]
. . . . . . . . . . . . Aotus vociferans .............    1 hits    1 orgs [Aotidae; Aotus]
. . . . . . . . . . . Catarrhini ....................   37 hits    4 orgs
. . . . . . . . . . . . Hominidae ...................   29 hits    3 orgs [Hominoidea]
. . . . . . . . . . . . . Homininae ..................   26 hits    2 orgs
. . . . . . . . . . . . . . Homo sapiens .............   22 hits    1 orgs [Homo]
. . . . . . . . . . . . . . Pan troglodytes ..........    4 hits    1 orgs [Pan]
. . . . . . . . . . . . . Pongo abelii ...............    3 hits    1 orgs [Ponginae; Pongo]
. . . . . . . . . . . . Macaca mulatta ...............    8 hits    1 orgs [Cercopithecoidea; Cercopithecidae; Cercopithecinae; Macaca]
. . . . . . . . . . Glires ..........................   13 hits    5 orgs
. . . . . . . . . . . Oryctolagus cuniculus ..........    1 hits    1 orgs [Lagomorpha; Leporidae; Oryctolagus]
. . . . . . . . . . . Sciurognathi ..................   12 hits    4 orgs [Rodentia]
. . . . . . . . . . . . Muroidea ....................   11 hits    3 orgs
. . . . . . . . . . . . . Murinae ....................    9 hits    2 orgs [Muridae]
. . . . . . . . . . . . . . Mus musculus .............    6 hits    1 orgs [Mus; Mus]
. . . . . . . . . . . . . . Rattus norvegicus .........    3 hits    1 orgs [Rattus]
. . . . . . . . . . . . . Cricetulus griseus .........    2 hits    1 orgs [Cricetidae; Cricetinae; Cricetulus]
. . . . . . . . . . . . Spermophilus tridecemlineatus .    1 hits    1 orgs [Sciuridae; Xerinae; Marmotini; Spermophilus]
. . . . . . . Neognathae ............................    4 hits    2 orgs [Sauropsida; Sauria; Archosauria; Dinosauria; Saurischia; Theropoda; C
. . . . . . . . Gallus gallus ........................    3 hits    1 orgs [Galliformes; Phasianidae; Phasianinae; Gallus]
. . . . . . . . Taeniopygia guttata ..................    1 hits    1 orgs [Passeriformes; Passeroidea; Estrildidae; Estrildinae; Taeniopygia]
. . . . . . Xenopus .................................    3 hits    2 orgs [Amphibia; Batrachia; Anura; Mesobatrachia; Pipoidea; Pipidae; Xenopod
. . . . . . . Xenopus (Silurana) tropicalis ...........    1 hits    1 orgs [Silurana]
. . . . . . . Xenopus laevis .........................    2 hits    1 orgs [Xenopus]
. . . . Eleutherozoa ................................    3 hits    2 orgs [Echinodermata]
. . . . . Strongylocentrotus purpuratus ..............    2 hits    1 orgs [Echinozoa; Echinoidea; Euechinoidea; Echinacea; Echinoida; Strongyloc
. . . . . Patiria miniata ............................    1 hits    1 orgs [Asterozoa; Asteroidea; Valvatacea; Valvatida; Asterinidae; Patiria]
. . . . Saccoglossus kowalevskii .....................    1 hits    1 orgs [Hemichordata; Enteropneusta; Harrimaniidae; Saccoglossus]
. . Trichoplax adhaerens .............................    1 hits    1 orgs [Placozoa; Trichoplax]
. synthetic construct ................................   11 hits    1 orgs [other sequences; artificial sequences]
```

**BLAST**

## Basic Local Alignment Search Tool

Jump to Page Content

**Job Title: EZ034459**

---

- 

### Tax BLAST Report

**Index**

- Lineage Report
- Organism Report
- Taxonomy Report
- Help

**Lineage Report**

```
root
. Metazoa      [animals]
. . Eumetazoa    [animals]
. . . Cnidaria      [cnidarians]
. . . . Hexacorallia [anthozoans]
. . . . . . Acropora millepora ------------------------       1 hit   [stony corals]        TSA: Acropora millepora SeqIndex6501, mRNA sequence
. . . . . . Nematostella vectensis ....................       1 hit   [sea anemones]        Nematostella vectensis predicted protein (NEMVEDRAFT v1g1
. . . . . Hydra magnipapillata ------------------------       2 hits  [hydrozoans]          PREDICTED: Hydra magnipapillata similar to predicted prot
. . . Ciona intestinalis ----------------------------       2 hits  [tunicates]           PREDICTED: Ciona intestinalis similar to Bcl2-associated
. . . Crepidula fornicata ...........................       1 hit   [gastropods]          TSA: Crepidula fornicata 8158.Cfedg
. . . Monodelphis domestica .........................       1 hit   [marsupials]          PREDICTED: Monodelphis domestica similar to Bax alpha (LO
. . . Strongylocentrotus purpuratus (purple urchin) .       2 hits  [sea urchins]         PREDICTED: Strongylocentrotus purpuratus similar to Bax a
. . . Plakobranchus ocellatus .......................       1 hit   [gastropods]          TSA: Placobranchus ocellatus 26778.Plocadult mRNA sequenc
. . . Mus musculus (mouse) ..........................       8 hits  [rodents]             Mus musculus osteoclast-like cell cDNA, RIKEN full-length
. . . Sus scrofa (wild boar) ........................       1 hit   [even-toed ungulates] Sus scrofa partial mRNA for Bax-alpha protein
. . . Tetraodon nigroviridis ........................       2 hits  [bony fishes]         Tetraodon nigroviridis full-length cDNA
. . . Canis lupus familiaris (dogs) .................       2 hits  [carnivores]          Canis lupus familiaris BCL2-associated X protein (BAX), m
. . . Felis catus (cat) .............................       2 hits  [carnivores]          Felis catus BCL2-associated X protein (BAX), mRNA >gi|255
. . . Bos taurus (cow) ..............................       4 hits  [even-toed ungulates] Bos taurus BCL2-associated X protein (BAX), mRNA >gi|1938
. . . Ovis aries (domestic sheep) ...................       1 hit   [even-toed ungulates] Ovis aries Bcl2-associated protein Bax (Bax) mRNA, partia
. . . Callithrix jacchus (common marmoset) ..........       1 hit   [primates]            PREDICTED: Callithrix jacchus apoptosis regulator BAX-lik
. . . Cervus elaphus (elk) ..........................       1 hit   [even-toed ungulates] Cervus elaphus bax mRNA, partial cds
. . . Pan troglodytes ...............................       1 hit   [primates]            PREDICTED: Pan troglodytes similar to Bax alpha (LOC74709
. . . Homo sapiens (man) ............................      21 hits  [primates]            Homo sapiens BCL2-associated X protein (BAX), transcript
. . . Macaca mulatta (rhesus macaque) ...............       4 hits  [primates]            PREDICTED: Macaca mulatta BCL2-associated X protein, tran
. . . Rattus norvegicus (brown rat) .................       8 hits  [rodents]             Rattus norvegicus Bcl2-associated X protein (Bax), mRNA >
. . . Danio rerio (zebra fish) ......................       4 hits  [bony fishes]         Danio rerio bcl2-associated X protein, a (baxa), mRNA >gi
. . . Xenopus laevis (common platanna) ..............       5 hits  [frogs & toads]       Xenopus laevis BCL2-associated X protein (bax), mRNA >gi|
. . . Oryctolagus cuniculus (domestic rabbit) .......       3 hits  [rabbits & hares]     PREDICTED: Oryctolagus cuniculus BCL2-associated X protei
. . . Xenopus (Silurana) tropicalis .................       3 hits  [frogs & toads]       Xenopus tropicalis finished cDNA, clone TGas091104
. . . Rattus sp. (rats) .............................       1 hit   [rodents]             bax=apoptosis inducer [rats, ovary, mRNA Partial, 402 nt]
. . . Cricetulus griseus (Chinese hamsters) .........       1 hit   [rodents]             Cricetulus griseus Bax mRNA, complete cds
. . . Mesocricetus auratus (Syrian hamster) .........       1 hit   [rodents]             Mesocricetus auratus partial mRNA for Bcl-2 associated pr
. . . Ictalurus punctatus ...........................       1 hit   [bony fishes]         Ictalurus punctatus clone CBZB25609 apoptosis regulator b
. . . Epinephelus coioides (estuary rock cod) .......       1 hit   [bony fishes]         Epinephelus coioides bcl2-associated X (BAX) mRNA, partia
. . . Ailuropoda melanoleuca ........................       1 hit   [carnivores]          PREDICTED: Ailuropoda melanoleuca apoptosis regulator BAX
. . . Taeniopygia guttata (zebra finch) .............       1 hit   [birds]               PREDICTED: Taeniopygia guttata similar to BCL2-associated
. . . Bos grunniens (yak) ...........................       1 hit   [even-toed ungulates] Bos grunniens apoptosis regulator bax-alpha (bax) mRNA, p
. . . Ictalurus furcatus ............................       2 hits  [bony fishes]         TSA: Ictalurus furcatus Contig02971.Icfu mRNA sequence
. . . Oncorhynchus mykiss ...........................       1 hit   [bony fishes]         Oncorhynchus mykiss clone omyk-evo-502-314 Apoptosis regu
. . . Gallus gallus (bantam) ........................       1 hit   [birds]               Gallus gallus myeloid cell leukemia protein MCL-1 mRNA, p
. . . Salmo salar ...................................       5 hits  [bony fishes]         Salmo salar clone ssal-eve-501-321 Apoptosis regulator BA
. . Trichoplax adhaerens ----------------------------       1 hit   [placozoans]          Trichoplax adhaerens hypothetical protein, mRNA
. synthetic construct -------------------------------       7 hits  [other sequences]     Synthetic construct Homo sapiens gateway clone IMAGE:1000
```

---

**Organism Report**

```
 Acropora millepora [stony corals] taxid 45264
gb|EZ034459.1|  TSA: Acropora millepora SeqIndex6501, mRNA ...          0.0

 Nematostella vectensis [sea anemones] taxid 45351
ref|XM_001634806.1| Nematostella vectensis predicted prote...          0.0

 Hydra magnipapillata [hydrozoans] taxid 6085
ref|XM_002157424.1| PREDICTED: Hydra magnipapillata simila...          0.0
ref|XM_002158033.1| PREDICTED: Hydra magnipapillata simila...          0.0

 Trichoplax adhaerens [placozoans] taxid 10228
ref|XM_002107647.1| Trichoplax adhaerens hypothetical prot...          0.0

 Ciona intestinalis [tunicates] taxid 7719
ref|XM_002122967.1| PREDICTED: Ciona intestinalis similar ...          0.0
dbj|AK116369.1|  Ciona intestinalis cDNA, clone:citb036h13,...          0.0

 Crepidula fornicata [gastropods] taxid 176853
gb|EZ548265.1|  TSA: Crepidula fornicata 8158.Cfedg                     0.0

 Monodelphis domestica [marsupials] taxid 13616
ref|XM_001379887.1| PREDICTED: Monodelphis domestica simil...          0.0

 Strongylocentrotus purpuratus (purple urchin, ...) [sea urchins] taxid 7668
ref|XM_001188066.1| PREDICTED: Strongylocentrotus purpurat...          0.0
ref|XM_786025.2|  PREDICTED: Strongylocentrotus purpuratus ...          0.0

 Plakobranchus ocellatus [gastropods] taxid 259542
gb|HP190622.1|  TSA: Placobranchus ocellatus 26778.Plocadul...          0.0

 Mus musculus (mouse) [rodents] taxid 10090
dbj|AK159233.1|  Mus musculus osteoclast-like cell cDNA, RI...          0.0
gb|AY095934.1|  Mus musculus Bcl2-associated X protein kapp...          0.0
ref|NM_007527.3|  Mus musculus BCL2-associated X protein (B...          0.0
gb|BC018228.1|  Mus musculus BCL2-associated X protein, mRN...          0.0
gb|BC053380.1|  Mus musculus BCL2-associated X protein, mRN...          0.0
dbj|AK013298.1|  Mus musculus 10, 11 days embryo whole body...          0.0
dbj|AK149994.1|  Mus musculus bone marrow macrophage cDNA, ...          0.0
gb|L22472.1|MUSBAXA Mouse Bax alpha mRNA, complete cds                 0.0
```

**Sus scrofa** (wild boar, ...) [even-toed ungulates] taxid 9823
emb|AJ606301.1| Sus scrofa partial mRNA for Bax-alpha protein ____ 0.0

**Tetraodon nigroviridis** [bony fishes] taxid 99883
emb|CR696948.1| Tetraodon nigroviridis full-length cDNA ____ 0.0
emb|CR668488.2| Tetraodon nigroviridis full-length cDNA ____ 0.0

**Canis lupus familiaris** (dogs) [carnivores] taxid 9615
ref|NM_001003011.1| Canis lupus familiaris BCL2-associated... ____ 0.0
dbj|AB080230.1| Canis lupus familiaris mRNA for Bax, compl... ____ 0.0

**Felis catus** (cat, ...) [carnivores] taxid 9685
ref|NM_001009282.1| Felis catus BCL2-associated X protein ... ____ 0.0
dbj|AB080724.2| Felis catus mRNA for bax-protein, complete... ____ 0.0

**Bos taurus** (cow, ...) [even-toed ungulates] taxid 9913
ref|NM_173894.1| Bos taurus BCL2-associated X protein (BAX... ____ 0.0
gb|U92569.1|BTU92569 Bos taurus apoptosis regulator bax-al... ____ 0.0
ref|NM_001191220.1| Bos taurus BCL2-associated X protein (... ____ 0.0
ref|XM_002701934.1| PREDICTED: Bos taurus BCL2-associated ... ____ 0.0

**Ovis aries** (domestic sheep, ...) [even-toed ungulates] taxid 9940
gb|AF163774.1|AF163774 Ovis aries Bcl2-associated protein ... ____ 0.0

**Callithrix jacchus** (common marmoset, ...) [primates] taxid 9483
ref|XM_002807856.1| PREDICTED: Callithrix jacchus apoptosi... ____ 0.0

**Cervus elaphus** (elk) [even-toed ungulates] taxid 9860
gb|AF512030.1| Cervus elaphus bax mRNA, partial cds ____ 0.0

**Pan troglodytes** [primates] taxid 9598
ref|XM_001155879.1| PREDICTED: Pan troglodytes similar to ... ____ 0.0

**Homo sapiens** (man) [primates] taxid 9606
ref|NM_138761.3| Homo sapiens BCL2-associated X protein (B... ____ 0.0
gb|BC014175.2| Homo sapiens BCL2-associated X protein, mRN... ____ 0.0
dbj|AK291076.1| Homo sapiens cDNA FLJ77115 complete cds, h... ____ 0.0
emb|CR595189.1| full-length cDNA clone CS0DE013YL22 of Pla... ____ 0.0
gb|L22473.1|HUMBAXA Human Bax alpha mRNA, complete cds ____ 0.0
emb|AJ417988.1| Homo sapiens mRNA for bax isoform psi (BAX... ____ 0.0
ref|NM_138763.3| Homo sapiens BCL2-associated X protein (B... ____ 0.0
gb|U19599.1|HSU19599 Human (BAX delta) mRNA, complete cds ____ 0.0
gb|AF250190.1|AF250190 Homo sapiens Bax zeta mRNA, complet... ____ 0.0
ref|NR_027882.1| Homo sapiens BCL2-associated X protein (B... ____ 0.0
gb|AF007826.1|AF007826 Homo sapiens bax epsilon mRNA, comp... ____ 0.0
ref|NM_004324.3| Homo sapiens BCL2-associated X protein (B... ____ 0.0
gb|L22474.1|HUMBAXB Human Bax beta mRNA, complete cds ____ 0.0
emb|AJ586910.1| Homo sapiens partial mRNA for bax protein ... ____ 0.0
emb|AJ586909.1| Homo sapiens partial mRNA for bax protein ... ____ 0.0
ref|NM_138764.4| Homo sapiens BCL2-associated X protein (B... ____ 0.0
gb|AF247393.1|AF247393 Homo sapiens bax-sigma mRNA, comple... ____ 0.0
gb|AY217036.1| Homo sapiens BCL2-associated X protein (BAX... ____ 0.0
ref|NG_012191.1| Homo sapiens BCL2-associated X protein (B... ____ 0.0
gb|AC015871.7| Homo sapiens chromosome 15, clone RP11-81A1... ____ 0.0
dbj|AK304026.1| Homo sapiens cDNA FLJ60214 complete cds, h... ____ 0.0

**Macaca mulatta** (rhesus macaque, ...) [primates] taxid 9544
ref|XM_002801334.1| PREDICTED: Macaca mulatta BCL2-associa... ____ 0.0
ref|XM_001112353.1| PREDICTED: Macaca mulatta BCL2-associa... ____ 0.0
ref|XM_002801333.1| PREDICTED: Macaca mulatta BCL2-associa... ____ 0.0
ref|XM_002801332.1| PREDICTED: Macaca mulatta BCL2-associa... ____ 0.0

**Rattus norvegicus** (brown rat, ...) [rodents] taxid 10116
ref|NM_017059.1| Rattus norvegicus Bcl2-associated X prote... ____ 0.0
gb|U49729.1|RRU49729 Rattus norvegicus rBax alpha mRNA, co... ____ 0.0
gb|AF235993.1|AF235993 Rattus norvegicus Bax protein splic... ____ 0.0
emb|FQ229844.1| Rattus norvegicus TL0ADA46YH24 mRNA sequence ____ 0.0
gb|U59184.1|RNU59184 Rattus norvegicus Bax-alpha mRNA, par... ____ 0.0
gb|U32098.1|RNU32098 Rattus norvegicus bax mRNA, partial cds ____ 0.0
ref|XM_001060981.2| PREDICTED: Rattus norvegicus similar t... ____ 0.0
ref|XM_001081479.2| PREDICTED: Rattus norvegicus similar t... ____ 0.0

**Danio rerio** (zebra fish, ...) [bony fishes] taxid 7955
ref|NM_131562.2| Danio rerio bcl2-associated X protein, a ... ____ 0.0
gb|BC055592.1| Danio rerio bcl2-associated X protein, a, m... ____ 0.0
gb|BC164990.1| Danio rerio bcl2-associated X protein, a, m... ____ 0.0
gb|AF231015.1|AF231015 Danio rerio Bax (bax) gene, complet... ____ 0.0

**Xenopus laevis** (common platanna, ...) [frogs & toads] taxid 8355
ref|NM_001085635.1| Xenopus laevis BCL2-associated X prote... ____ 0.0
gb|AF288809.1|AF288809 Xenopus laevis bax mRNA, complete cds ____ 0.0
gb|BC169589.1| Xenopus laevis BCL2-associated X protein, m... ____ 0.0
gb|BC169587.1| Xenopus laevis BCL2-associated X protein, m... ____ 0.0
gb|AY437085.1| Xenopus laevis Bax mRNA, complete cds ____ 0.0

**Oryctolagus cuniculus** (domestic rabbit, ...) [rabbits & hares] taxid 9986
ref|XM_002723696.1| PREDICTED: Oryctolagus cuniculus BCL2-... ____ 0.0
ref|XM_002723697.1| PREDICTED: Oryctolagus cuniculus BCL2-... ____ 0.0
ref|XM_002722959.1| PREDICTED: Oryctolagus cuniculus BCL2-... ____ 0.0

**Xenopus (Silurana) tropicalis** [frogs & toads] taxid 8364
emb|CR855728.2| Xenopus tropicalis finished cDNA, clone TG... ____ 0.0
ref|NM_203854.1| Xenopus (Silurana) tropicalis BCL2-associ... ____ 0.0
gb|BC063201.1| Xenopus tropicalis BCL2-associated X protei... ____ 0.0

**Rattus sp.** (rats) [rodents] taxid 10118
gb|S76511.1| bax=apoptosis inducer [rats, ovary, mRNA Part... ____ 0.0

**Cricetulus griseus** (Chinese hamsters) [rodents] taxid 10029
gb|EF104643.1| Cricetulus griseus Bax mRNA, complete cds ____ 0.0

**Mesocricetus auratus** (Syrian hamster, ...) [rodents] taxid 10036
emb|AJ582075.1| Mesocricetus auratus partial mRNA for Bcl-... ____ 0.0

**Ictalurus punctatus** [bony fishes] taxid 7998
gb|GU589276.1| Ictalurus punctatus clone CBZB25609 apoptos... ____ 0.0

```
 synthetic construct [other sequences] taxid 32630
emb|CU680816.1|  Synthetic construct Homo sapiens gateway c...     ____   0.0
gb|EU716633.1|  Synthetic construct N-EGFP/BCL2-associated ...     ____   0.0
dbj|AB528719.1|  Synthetic construct DNA, clone: pF1KB6979,...     ____   0.0
gb|AY891226.1|  Synthetic construct Homo sapiens clone FLH1...     ____   0.0
gb|AY888578.1|  Synthetic construct Homo sapiens clone FLH1...     ____   0.0
gb|AY893763.1|  Synthetic construct Homo sapiens clone FLH0...     ____   0.0
gb|AY893312.1|  Synthetic construct Homo sapiens clone FLH0...     ____   0.0

 Epinephelus coioides (estuary rock cod, ...) [bony fishes] taxid 94232
gb|AY735005.1|  Epinephelus coioides bcl2-associated X (BAX...     ____   0.0

 Ailuropoda melanoleuca [carnivores] taxid 9646
ref|XM_002917916.1|  PREDICTED: Ailuropoda melanoleuca apop...     ____   0.0

 Taeniopygia guttata (zebra finch) [birds] taxid 59729
ref|XM_002199674.1|  PREDICTED: Taeniopygia guttata similar...     ____   0.0

 Bos grunniens (yak) [even-toed ungulates] taxid 30521
gb|AY450350.1|  Bos grunniens apoptosis regulator bax-alpha...     ____   0.0

 Ictalurus furcatus [bony fishes] taxid 66913
gb|HP432380.1|  TSA: Ictalurus furcatus Contig02971.Icfu mR...     ____   0.0
gb|GU588167.1|  Ictalurus furcatus clone CBZF10821 apoptosi...     ____   0.0

 Oncorhynchus mykiss [bony fishes] taxid 8022
gb|BT074328.1|  Oncorhynchus mykiss clone omyk-evo-502-314 ...     ____   0.0

 Gallus gallus (bantam, ...) [birds] taxid 9031
gb|AF120210.1|AF120210 Gallus gallus myeloid cell leukemia...      ____   0.0

 Salmo salar [bony fishes] taxid 8030
gb|BT048648.1|  Salmo salar clone ssal-eve-501-321 Apoptosi...     ____   0.0
gb|BT044708.1|  Salmo salar clone ssal-rgf-501-217 Apoptosi...     ____   0.0
gb|BT050190.1|  Salmo salar clone ssal-evd-538-260 Apoptosi...     ____   0.0
gb|BT125178.1|  Salmo salar clone ssal-eve-507-043 Apoptosi...     ____   0.0
gb|BT047644.2|  Salmo salar clone ssal-eve-576-277 Apoptosi...     ____   0.0
```

---

**Taxonomy Report**

```
root ...............................................  107 hits   39 orgs
. Metazoa ..........................................  100 hits   38 orgs [cellular organisms; Eukaryota; Fungi/Metazoa group]
. . Eumetazoa ......................................   99 hits   37 orgs
. . . Cnidaria .....................................    4 hits    3 orgs
. . . . Hexacorallia ...............................    2 hits    2 orgs [Anthozoa]
. . . . . Acropora millepora .......................    1 hits    1 orgs [Scleractinia; Astrocoeniina; Acroporidae; Acropora]
. . . . . Nematostella vectensis ...................    1 hits    1 orgs [Actiniaria; Edwardsiidae; Nematostella]
. . . . Hydra magnipapillata .......................    2 hits    1 orgs [Hydrozoa; Hydroida; Anthomedusae; Hydridae; Hydra]
. . . Coelomata ....................................   95 hits   34 orgs [Bilateria]
. . . . Deuterostomia ..............................   93 hits   32 orgs
. . . . . Chordata .................................   91 hits   31 orgs
. . . . . . Ciona intestinalis .....................    2 hits    1 orgs [Tunicata; Ascidiacea; Enterogona; Phlebobranchia; Cionidae; Ciona]
. . . . . . Euteleostomi ...........................   89 hits   30 orgs [Craniata; Vertebrata; Gnathostomata; Teleostomi]
. . . . . . . Tetrapoda ............................   73 hits   23 orgs [Sarcopterygii]
. . . . . . . . Amniota ............................   65 hits   21 orgs
. . . . . . . . . Theria ...........................   63 hits   19 orgs [Mammalia]
. . . . . . . . . . Monodelphis domestica ..........    1 hits    1 orgs [Metatheria; Didelphimorphia; Didelphidae; Didelphinae; Monodelphis]
. . . . . . . . . . Eutheria .......................   62 hits   18 orgs
. . . . . . . . . . . Euarchontoglires .............   49 hits   10 orgs
. . . . . . . . . . . . Glires .....................   22 hits    6 orgs
. . . . . . . . . . . . . Muroidea .................   19 hits    5 orgs [Rodentia; Sciurognathi]
. . . . . . . . . . . . . . Murinae ................   17 hits    3 orgs [Muridae]
. . . . . . . . . . . . . . . Mus musculus .........    8 hits    1 orgs [Mus; Mus]
. . . . . . . . . . . . . . . Rattus ...............    9 hits    2 orgs
. . . . . . . . . . . . . . . . Rattus norvegicus ..    8 hits    1 orgs
. . . . . . . . . . . . . . . . Rattus sp. .........    1 hits    1 orgs [unclassified Rattus]
. . . . . . . . . . . . . . Cricetinae .............    2 hits    2 orgs [Cricetidae]
. . . . . . . . . . . . . . . Cricetulus griseus ...    1 hits    1 orgs [Cricetulus]
. . . . . . . . . . . . . . . Mesocricetus auratus .    1 hits    1 orgs [Mesocricetus]
. . . . . . . . . . . . . Oryctolagus cuniculus ....    3 hits    1 orgs [Lagomorpha; Leporidae; Oryctolagus]
. . . . . . . . . . . . Simiiformes ................   27 hits    4 orgs [Primates; Haplorrhini]
. . . . . . . . . . . . . Callithrix jacchus .......    1 hits    1 orgs [Platyrrhini; Cebidae; Callitrichinae; Callithrix]
. . . . . . . . . . . . . Catarrhini ...............   26 hits    3 orgs
. . . . . . . . . . . . . . Homininae ..............   22 hits    2 orgs [Hominoidea; Hominidae]
. . . . . . . . . . . . . . . Pan troglodytes ......    1 hits    1 orgs [Pan]
. . . . . . . . . . . . . . . Homo sapiens .........   21 hits    1 orgs [Homo]
. . . . . . . . . . . . . . Macaca mulatta .........    4 hits    1 orgs [Cercopithecoidea; Cercopithecidae; Cercopithecinae; Macaca]
. . . . . . . . . . . Laurasiatheria ...............   13 hits    8 orgs
. . . . . . . . . . . . Cetartiodactyla ............    8 hits    5 orgs
. . . . . . . . . . . . . Sus scrofa ...............    1 hits    1 orgs [Suina; Suidae; Sus]
. . . . . . . . . . . . . Pecora ...................    7 hits    4 orgs [Ruminantia]
. . . . . . . . . . . . . . Bovidae ................    6 hits    3 orgs
. . . . . . . . . . . . . . . Bos ..................    5 hits    2 orgs [Bovinae]
. . . . . . . . . . . . . . . . Bos taurus .........    4 hits    1 orgs
. . . . . . . . . . . . . . . . Bos grunniens ......    1 hits    1 orgs
. . . . . . . . . . . . . . . Ovis aries ...........    1 hits    1 orgs [Caprinae; Ovis]
. . . . . . . . . . . . . . Cervus elaphus .........    1 hits    1 orgs [Cervidae; Cervinae; Cervus]
. . . . . . . . . . . . Carnivora ..................    5 hits    3 orgs
. . . . . . . . . . . . . Caniformia ...............    3 hits    2 orgs
. . . . . . . . . . . . . . Canis lupus familiaris .    2 hits    1 orgs [Canidae; Canis; Canis lupus]
. . . . . . . . . . . . . . Ailuropoda melanoleuca .    1 hits    1 orgs [Ursidae; Ailuropoda]
. . . . . . . . . . . . . Felis catus ..............    2 hits    1 orgs [Feliformia; Felidae; Felinae; Felis]
. . . . . . . . . Neognathae .......................    2 hits    2 orgs [Sauropsida; Sauria; Archosauria; Dinosauria; Saurischia; Theropoda; Coel
. . . . . . . . . . Taeniopygia guttata ............    1 hits    1 orgs [Passeriformes; Passeroidea; Estrildidae; Estrildinae; Taeniopygia]
. . . . . . . . . . Gallus gallus ..................    1 hits    1 orgs [Galliformes; Phasianidae; Phasianinae; Gallus]
. . . . . . . . Xenopus ............................    8 hits    2 orgs [Amphibia; Batrachia; Anura; Mesobatrachia; Pipoidea; Pipidae; Xenopodina
. . . . . . . . . Xenopus laevis ...................    5 hits    1 orgs [Xenopus]
. . . . . . . . . Xenopus (Silurana) tropicalis ....    3 hits    1 orgs [Silurana]
. . . . . . . Clupeocephala ........................   16 hits    7 orgs [Actinopterygii; Actinopteri; Neopterygii; Teleostei; Elopocephala]
. . . . . . . . Euteleostei ........................    9 hits    4 orgs
. . . . . . . . . Percomorpha ......................    3 hits    2 orgs [Neognathi; Neoteleostei; Eurypterygii; Ctenosquamata; Acanthomorpha; Eua
. . . . . . . . . . Tetraodon nigroviridis .........    2 hits    1 orgs [Tetraodontiformes; Tetraodontoidei; Tetradontoidea; Tetraodontidae; Tetr
. . . . . . . . . . Epinephelus coioides ...........    1 hits    1 orgs [Perciformes; Percoidei; Serranidae; Epinephelinae; Epinephelus]
. . . . . . . . . Salmoninae .......................    6 hits    2 orgs [Protacanthopterygii; Salmoniformes; Salmonoidei; Salmonidae]
. . . . . . . . . . Oncorhynchus mykiss ............    1 hits    1 orgs [Oncorhynchus]
. . . . . . . . . . Salmo salar ....................    5 hits    1 orgs [Salmo]
. . . . . . . . Otophysi ...........................    7 hits    3 orgs [Otocephala; Ostariophysi]
. . . . . . . . . Danio rerio ......................    4 hits    1 orgs [Cypriniphysi; Cypriniformes; Cyprinoidea; Cyprinidae; Danio]
. . . . . . . . . Ictalurus ........................    3 hits    2 orgs [Siluriphysi; Siluriformes; Ictaluridae]
. . . . . . . . . . Ictalurus punctatus ............    1 hits    1 orgs
. . . . . . . . . . Ictalurus furcatus .............    2 hits    1 orgs
. . . . . Strongylocentrotus purpuratus ............    2 hits    1 orgs [Echinodermata; Eleutherozoa; Echinozoa; Echinoidea; Euechinoidea; Echina
. . . . Apogastropoda ..............................    2 hits    2 orgs [Protostomia; Mollusca; Gastropoda; Orthogastropoda]
```

```
. . . . . Crepidula fornicata .....................     1 hits    1 orgs [Caenogastropoda; Sorbeoconcha; Hypsogastropoda; Littorinimorpha; Calyptr
. . . . . Plakobranchus ocellatus .................     1 hits    1 orgs [Heterobranchia; Euthyneura; Opisthobranchia; Sacoglossa; Placobranchoide
. . Trichoplax adhaerens ..........................     1 hits    1 orgs [Placozoa; Trichoplax]
. synthetic construct .............................     7 hits    1 orgs [other sequences; artificial sequences]
```
